# Deterministic-stochastic analysis of fractional differential equations malnutrition model with random perturbations and crossover effects

**DOI:** 10.1038/s41598-023-41861-4

**Published:** 2023-09-08

**Authors:** Yu-Ming Chu, Saima Rashid, Shazia Karim, Aasma Khalid, S. K. Elagan

**Affiliations:** 1 Department of Mathematics, Faculty of Sciences, Huzhou University, Huzhou, China; 2 Department of Mathematics, Government College University, Faisalabad, 38000 Pakistan; 3 Department of Computer Science and Mathematics, Lebanese American University, Beirut, 1401 Lebanon; 4 Department of Basic Sciences and Humanities, UET Lahore, Faisalabad Campus, 54800 Pakistan; 5 Department of Mathematics, Government College for Women University, Faisalabad, Pakistan; 6 Department of Mathematics and Statistics, College of Science, Taif University, P. O. Box 11099, 21944 Taif, Saudi Arabia

**Keywords:** Biophysics, Developmental biology

## Abstract

To boost the handful of nutrient-dense individuals in the societal structure, adequate health care documentation and comprehension are permitted. This will strengthen and optimize the well-being of the community, particularly the girls and women of the community that are welcoming the new generation. In this article, we extensively explored a deterministic-stochastic malnutrition model involving nonlinear perturbation via piecewise fractional operators techniques. This novel concept leads us to analyze and predict the process from the beginning to the end of the well-being growth, as it offers the possibility to observe many behaviors from cross over to stochastic processes. Moreover, the piecewise differential operators, which can be constructed with operators such as classical, Caputo, Caputo-Fabrizio, Atangana-Baleanu and stochastic derivative. The threshold parameter is developed and the role of malnutrition in society is examined. Through a rigorous analysis, we first demonstrated that the stochastic model’s solution is positive and global. Then, using appropriate stochastic Lyapunov candidates, we examined whether the stochastic system acknowledges a unique ergodic stationary distribution. The objective of this investigation is to design a nutritional deficiency in pregnant women using a piecewise fractional differential equation scheme. We examined multiple options and outlined numerical methods of coping with problems. To exemplify the effectiveness of the suggested concept, graphical conclusions, including chaotic and random perturbation patterns, are supplied. Consequently, fractional calculus’ innovative aspects provide more powerful and flexible layouts, enabling us to more effectively adapt to the system dynamics tendencies of real-world representations. This has opened new doors to readers in different disciplines and enabled them to capture different behaviors at different time intervals.

## Introduction

From conception to old age, diet has been a dominant problem in each process of biological progression. The performance of nourishment influences the lifestyle, especially the mental well-being of a pregnant woman^1^. When a lactating mothers are malnourished, the foetus in the womb faces numerous challenging situations from childhood to adulthood. The effectiveness of a pregnant woman’s medical coverage is critical to the well-being of the baby to be born^2^. However, nutritional supplementation or deficiency has an effect on the child’s weight. Birthweight is defined as a newborn infant weighing just under 2.5 kg. Infant mortality, economic growth, intellectual advancement, head trauma, iron deficiency, low body weight and other symptoms that characterize this scenario^3, 4^.

Fetuses are often placed in contemporary ventures for a time frame and then survived. The performance of the life process is determined by the supplements absorbed by the expecting mothers throughout this phase. In underdeveloped nations, including the Sub-Saharan continent, the circumstances are even more severe; numerous communities struggle to provide pregnant women with nutritious meals. So, several pregnant women are refused a balanced meal in certain parts of the country owing to religious and societal beliefs^5–7^.

An underdeveloped infant is characterized by a newborn whose size is significantly less than what he or she should be at a certain age as a result of poor diet throughout pregnancy. Other contributing considerations to this scenario include genetics, food insecurity and unbalanced nutrition^8^. In fact, when a child reaches adolescence, hedgerows seem to become a critical concern^9^. A variety of therapies are available to help alleviate the symptoms of prepubescent hedgerows. For example, UNICEF aims to enhance the nourishment of juvenile girls as they are presumably pregnant women^10^. It has been identified that a female’s initial period of teenage advancement necessitates additional power and vitamins, and UNICEF offers nutrient and folic probiotics via training throughout this phase of growth^11, 12^. The evolution is the foundation of humanity and necessitates investment in the supply of enough nutrient content to living creatures, especially pregnant women^13, 14^.

A wide range of research has now developed that mathematical modelling is an indispensable tool for analyzing socio-cultural issues and delivering cost-effective solutions. There are a number of computational forms on infection trends^15, 16^, whereas there is little documentation on the numerical techniques of an entire lifespan. All analyses on under-nutrition have emphasized local distinctiveness with no compelling rationalization. It is pertinent to mention that the community delineation has dropped beyond expectations due to the nonlocality consequences of mathematical structure. As a result, computational difficulties describing the nonlocality of scientific processes are necessitated. Among previous techniques, fractional calculus (FC) has the distinctive and exclusive property of documenting memory impact, which is also encountered in nearly all biochemical mechanisms.

The FC has played an important role in the simulation of bacterial infections^17–24^. To comprehend the efficacy of environmental factors, multiple fractional formulas have been used, such as Caputo^25^, Caputo-Fabrizio^26^, and Atangana-Balenau operator^27^. However, due to complexities of several real world problems, these classes of differential equations have failed several times to replicate the observed facts. For example, several real world problems displaying some randomness that could not be captured by these differential equations, thus, the concept of stochastic differential equations have been suggested and used intensively in the last decades with some great successes. However, some problems did not follow randomness, instead they follow some trends of non-localities, including fading memory, long range dependence, memory effect, power law process, anomalous process, fractal processes, crossover behaviors meaning a physical problem displays multiple behaviors. To solve these issues, a range of differential operators were suggested, including fractal differential operators, fractional derivatives with singular kernels, fractional derivatives with non-singular kernels, fractal-fractional differential operators and differential operators with respect to other functions^17–19^. These differential operators have given birth to different classes of ordinary and partial differential equations that have been used to solve many problems with great success. Nevertheless, the problem of crossover behaviors has not been clearly solved. In the case of fractional differential and integral operators, the behaviors of their kernels are analyzed, unlike the power law kernel, exponential decay and the generalized Mittag-Leffler functions are found to exhibit crossover behaviors^26, 27^. A physical property that is observed in many real-world problems, including: biological modelling, diffusion, advection, flow of fluid in complex media and many others^28–30^. Nevertheless, although these crossover properties of the Mittag-Leffler function and the exponential function have been recognized as a powerful mathematical tool to depict real world problems, one should note that, only real world problem following the crossover properties of these two functions can be modeled with some limitations as in real-world problems; these two functions will not be able to establish the time at which the crossover took place. Indeed, real-world problems exhibit different processes that are presented by the generalized Mittag-Leffler function and exponential decay function cannot be replicated using the Caputo-Fabrizio and Atangana-Baleanu derivatives. For example, if a real-world problem presents first a power law process, then later a fading memory process, it is clear that neither the general Mittag-Leffler or exponential decay functions will not be able to capture such behavior. In this paper, we will introduce different classes of differential and integral operators called piecewise derivatives and integrals. These operators will be used to deal with problems exhibiting crossover behaviors. For such methodologies, many appropriate analysis estimates for tackling various types of fractional differential equations were additionally developed^17–19^. Nonetheless, a few have made attempts to analyze such methodologies in an attempt to identify the most successful one. Rashid and Jarad^31^ presented the qualitative analysis of a stochastic fractal-fractional Ebola epidemic model combining fear and environmental spreading mechanisms with a Mittag-Leffler kernel. Furthermore, the researchers^32^ expounded the global positive solutions of the dengue infection system pertaining to multi-receptors using general kernels in the Atangana-Baleanu sense. Nosrati et al.^33^ presented the extended fractional singular Kalman filter using stochastic reasoning. Wei et al.^34^ contemplated an improved pseudo-state estimator for a class of commensurate fractional-order linear systems based on fractional modulating functions. Nosrati et al.^35^ expounded the optimal robust filter for uncertain fractional-order systems. This modern understanding, in contradiction to traditional fractional challenges, produces a different conception of nonlocal progression. Also, the performance of memory kernels is examined in the case of fractional derivative/integral formulations; in addition to the index-law kernel, exponential decay and the generalized Mittag-Leffler functions are revealed to illustrate crossover interactions. Atangana and Seda have successfully created new features widely recognized as piecewise differentiation and integration, where a contemporary interpretation is mentioned as a piecewise within a predefined duration^36^. This is a previously developed quantitative instrument for highlighting important challenges with complex cross-over traits. The innovative breed of simulation^37^ will address a wide range of underlying problems. A schematic view has been indicated in a variety of real-world applications, such as medical application modelling, propagation, thermal conduction in artificial media, and numerous others^38, 39^.

As of now, the malnutrition model with nonlinear perturbations has received little consideration. The goal of this research is to create a new deterministic-stochastic mathematical model that will examine fresh insights into pregnant women’s malnutrition conditions via crossover behaviours. The concept of piecewise differential and integral operators is applied in the framework of fractional differential operators and stochastic schemes. It is reported that the generalized Mittag-Leffler kernel and exponential decay functions are able to depict some crossover behaviours, but their abilities to achieve this may be limited due to the complexity of nature. In this implementation, we employ the broader identity of fractional differential equations with singular and nonsingular kernels^36^. Furthermore, the deterministic and stochastic aspects of the model are discussed in a detailed manner. Ergodicity and stationary distribution analyses are carried out. Additionally, simulation analysis of the proposed model exhibits cross-over from deterministic to stochastic or vice versa. We noticed a very peculiar way of expansion exhibited by the malnutrition model, in which the distribution demonstrates an indication of determinism within a specific time period and then reveals a gesture of stochastic unpredictability. As a result, this truly innovative model has the capability to portray important features of the malnutrition mechanism throughout the entire life-cycle better than the classic techniques.

The following describes the article being presented. First, the important mathematical interpretations and representations are introduced. Then, in stochastic randomness, we display the mathematical description of the proposed model. Following that, we evaluate the suggested model’s global positive solutions. Furthermore, the ergodicity and stationary distribution (ESD) of the solution associated with the malnutrition system are discussed in “Qualitative aspects of proposed model” section. “Numerical simulation” section develops a numerical technique for solving the fractional model under deliberation. Eventually, we display our numerical outcomes and contrast them to those acquired using the piecewise fractional differential equations methodologies in “Results and discussion” section. In a nutshell, we explain the accumulated realities of our research results in our conclusion part.

## Model and preliminaries

**Figure 1:**
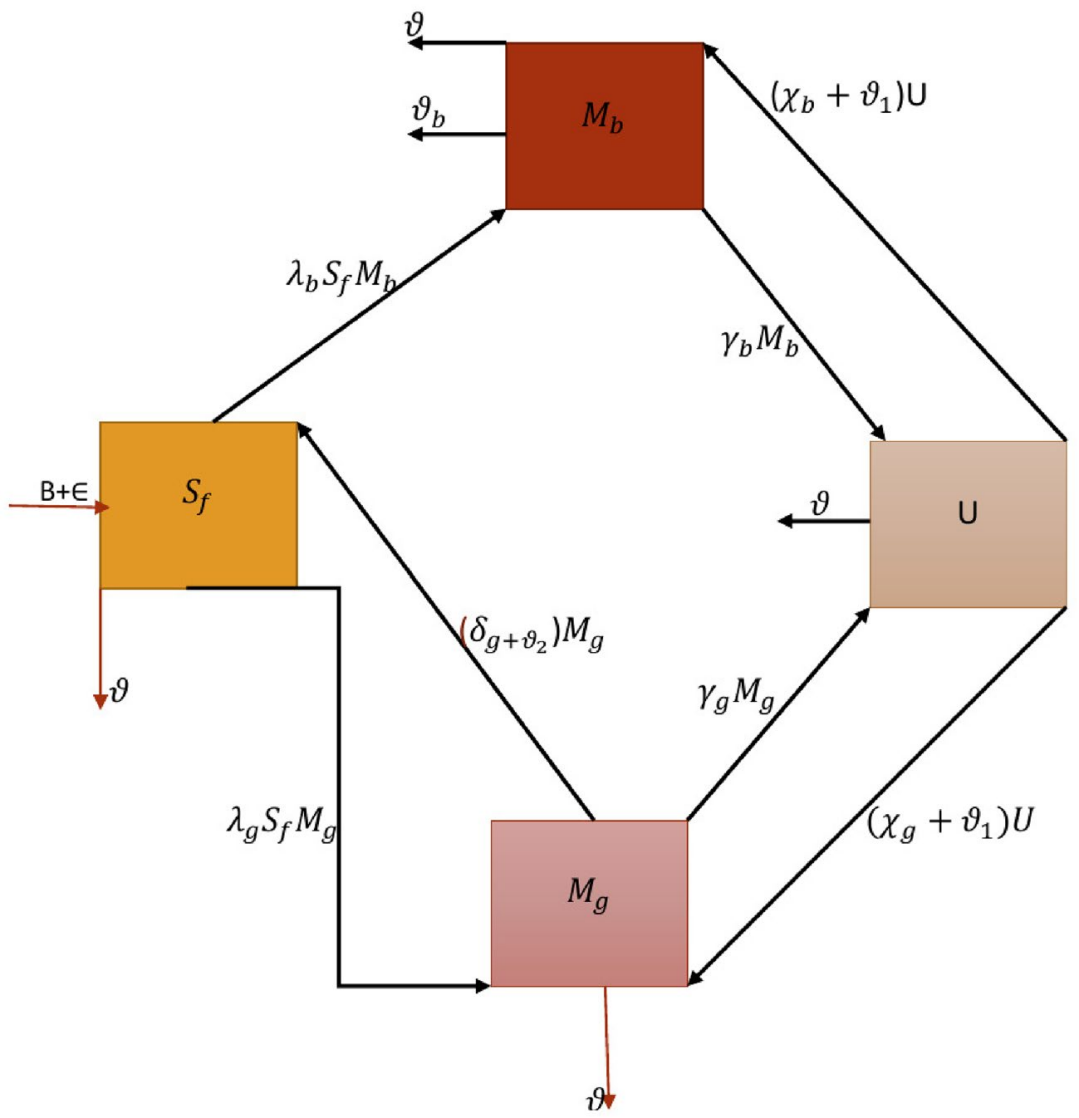
Flow diagram of malnutrition and underweight.

This portion describes a mathematical model of nutritional deficiencies in pregnant women. Figure 1 depicts the evolution of this concept. Table 1 contains all requirements and their understandings.

**Table 1:** Explanation of system’s feature.

Symbols	Explanation	Values
$\mathcal {S}_{\textbf{f}}$	Number of females susceptible to nutrients	30
$\bar{\mathcal {M}}_{\textbf{b}}$	Number of poor nutrient boys	2
$\bar{\mathcal {M}}_{\textbf{g}}$	Number of poor nutrient girls	4
$\bar{\mathcal {U}}$	Percentage of underweight individuals	1
$\mathcal {B}$	Generation of new female	0.01 day$^{-1}$
$\varepsilon$	Vertical spread to a new born community	0.001 day$^{-1}$
$\lambda _{\textbf{b}}$	Emaciated boy’s transfer rate from vulnerable female cohort	0.1 day$^{-1}$
$\lambda _{\textbf{g}}$	Emaciated girl’s transfer rate from vulnerable female cohort	0.2 day$^{-1}$
$\vartheta$	Death rate	0.1 day$^{-1}$
$\vartheta _{\textbf{b}}$	Natural restoration rate	0.3 day$^{-1}$
$\gamma _{\textbf{b}}$	The proportion of boys who progress from malnourishment to underweight	0.01 cell ml$^{-1}$ day$^{-1}$ person$^{-1}$
$\gamma _{\textbf{g}}$	The proportion of girls who progress from malnourishment to underweight	0.1 day$^{-1}$
$\chi _{\textbf{b}}$	Proportion of underweight shifted to $\bar{\mathcal {M}}_{\textbf{b}}$	0.014 day$^{-1}$
$\chi _{\textbf{g}}$	Proportion of underweight shifted to $\bar{\mathcal {M}}_{\textbf{g}}$	0.01 day$^{-1}$
$\vartheta _{1}$	Health promoting candidacy evaluation (per day)	[0,1]
$\vartheta _{2}$	Undernourished girl’s treatment rate (per day)	[0,1]
$\delta _{\textbf{g}}$	Undernourished girl’s recovery rate	0.1 day$^{-1}$

Malnourished pregnant women $\mathcal {S}_{\textbf{f}}$ lead up to malnourished boys $\bar{\mathcal {M}}_{\textbf{b}}$ and girls $\bar{\mathcal {M}}_{\textbf{g}}$. Boys and girls are undernourished as a result of vulnerable females at propagation rates $\lambda _{\textbf{b}}$ and $\lambda _{\textbf{g}}$, respectively. Even before low-weight newborns are not granted immediate healthcare treatment, they develop into underdeveloped kids at rates of $\gamma _{\textbf{b}}$ and $\gamma _{\textbf{g}}$ for both genders, respectively. The fundamental fatality rate is signified by $\vartheta$, and the handful of underweight people is symbolized by $\bar{\mathcal {U}}$. Furthermore, $\textbf{N}_{h}$ determines the the entire community, where $\textbf{N}_{h}=\mathcal {S}_{\textbf{f}}+\bar{\mathcal {M}}_{\textbf{b}}+\bar{\mathcal {M}}_{\textbf{g}}+\bar{\mathcal {U}}.$ The respective complex differential equations framework illustrates malnutrition at various varying phases of an entire evolution^40^:

2.1 $$\begin{aligned} {\left\{ \begin{array}{ll} \frac{d\mathcal {S}_{\textbf{f}}}{d\zeta }=(\mathcal {B}+\varepsilon )-(\lambda _{\textbf{b}}\bar{\mathcal {M}}_{\textbf{b}} +\lambda _{\textbf{g}}\bar{\mathcal {M}}_{\textbf{g}}+\vartheta )\mathcal {S}_{\textbf{f}} +\delta _{\textbf{g}}\bar{\mathcal {M}}_{\textbf{g}},\\ \frac{d\bar{\mathcal {M}}_{\textbf{b}}}{d\zeta }=\lambda _{\textbf{b}}\mathcal {S}_{\textbf{f}}\bar{\mathcal {M}}_{\textbf{b}} -(\vartheta _{\textbf{b}}+\gamma _{\textbf{b}}+\vartheta )\bar{\mathcal {M}}_{\textbf{b}}+\chi _{\textbf{b}}\bar{\mathcal {U}},\\ \frac{d\bar{\mathcal {M}}_{\textbf{g}}}{d\zeta }=\lambda _{\textbf{g}}\mathcal {S}_{\textbf{f}}\bar{\mathcal {M}}_{\textbf{g}} -(\gamma _{\textbf{g}}+\delta _{\textbf{g}}+\vartheta )\bar{\mathcal {M}}_{\textbf{g}}+\chi _{\textbf{g}}\bar{\mathcal {U}},\\ \frac{d\bar{\mathcal {U}}_{\textbf{b}}}{d\zeta }=\gamma _{\textbf{b}}\bar{\mathcal {M}}_{\textbf{b}} +\gamma _{\textbf{g}}\bar{\mathcal {M}}_{\textbf{g}}-(\chi _{\textbf{b}}+\chi _{\textbf{g}}+\vartheta )\bar{\mathcal {U}}, \end{array}\right. } \end{aligned}$$

where $\varepsilon$ indicates the progression of disease to a newly born community and $\mathcal {B}$ signifies the new female recruitment rate. In (2.1), the other specifications $\delta _{\textbf{g}}$, $\chi _{\textbf{b}}$, $\chi _{\textbf{g}}$ depict the restoration proportion of malnutrition girls and the proportions of underweight newborns progressing to $\bar{\mathcal {M}}_{\textbf{b}}$ and $\bar{\mathcal {M}}_{\textbf{g}}$, respectively. Because framework (2.1) is concerned with human population demographics, all specifications and system specifications are assumed to be non-negative, respectively^21, 40^. The mathematical formulation provided by (2.1) has been investigated earlier in^40^ to explore the propagation of food insecurity and underweight participants in a community. This framework, even so, excludes the consequences of memory, which are present in several natural systems. When analyzing the existence of stochastic processes using the Has’minskii concept^41^, the challenges experienced include how to assemble a Lyapunov function and determining an appropriate subset such that the dispersion operator is negative beyond the subset. Encouraged by the monitoring and evaluation process, we contemplate the stochastic theory of underweight four-species cooperative frameworks in this article as follows:

2.2 $$\begin{aligned} {\left\{ \begin{array}{ll} d{\mathcal {S}_{\textbf{f}}}(\zeta )=\big ((\mathcal {B}+\varepsilon )-(\lambda _{\textbf{b}}\bar{\mathcal {M}}_{\textbf{b}} +\lambda _{\textbf{g}}\bar{\mathcal {M}}_{\textbf{g}}+\vartheta )\mathcal {S}_{\textbf{f}} +\delta _{\textbf{g}}\bar{\mathcal {M}}_{\textbf{g}}\big )+\sigma _{1}\mathcal {S}_{\textbf{f}}(\zeta )d\mathcal {W}_{1}(\zeta ),\\ d{\bar{\mathcal {M}}_{\textbf{b}}}(\zeta )=\big (\lambda _{\textbf{b}}\mathcal {S}_{\textbf{f}}\bar{\mathcal {M}}_{\textbf{b}} -(\vartheta _{\textbf{b}}+\gamma _{\textbf{b}}+\vartheta )\bar{\mathcal {M}}_{\textbf{b}}+\chi _{\textbf{b}}\bar{\mathcal {U}}\big ) +\sigma _{2}\bar{\mathcal {M}}_{\textbf{b}}(\zeta )d\mathcal {W}_{2}(\zeta ),\\ d{\bar{\mathcal {M}}_{\textbf{g}}}(\zeta )=\big (\lambda _{\textbf{g}}\mathcal {S}_{\textbf{f}}\bar{\mathcal {M}}_{\textbf{g}} -(\gamma _{\textbf{g}}+\delta _{\textbf{g}}+\vartheta )\bar{\mathcal {M}}_{\textbf{g}}+\chi _{\textbf{g}}\bar{\mathcal {U}}\big ) +\sigma _{3}\bar{\mathcal {M}}_{\textbf{g}}(\zeta )d\mathcal {W}_{3}(\zeta ),\\ d{\bar{\mathcal {U}}}(\zeta )=\big (\gamma _{\textbf{b}}\bar{\mathcal {M}}_{\textbf{b}} +\gamma _{\textbf{g}}\bar{\mathcal {M}}_{\textbf{g}}-(\chi _{\textbf{b}} +\chi _{\textbf{g}}+\vartheta )\bar{\mathcal {U}}\big )+\sigma _{4}\bar{\mathcal {U}}(\zeta )d\mathcal {W}_{r}(\zeta ), \end{array}\right. } \end{aligned}$$

where $\mathcal {W}_{\Bbbk }(\zeta ),~\Bbbk =1,...,4$ denotes the standard one-dimensional Brownian motion described on a complete filtered probability space $\{\tilde{\mho },\mathfrak {P},\{\mathfrak {P}_{\zeta }\}_{\zeta \ge 0},\mathcal {P}\}$ having a $\sigma$-filtration $\{\mathfrak {P}_{\zeta }\}_{\zeta \ge 0}.$ Also, $\sigma _{\Bbbk },~\Bbbk =1,...,4$ is the white noise intensity.

The framework is hypothesized all segmentation based this inquiry (2.1) is acknowledged as a complete probability space $(\tilde{\mho },\mathfrak {P},\{\mathfrak {P}_{\zeta }\}_{\zeta >0},\mathcal {P})$ having a right continuous filtration $\{\mathfrak {P}_{\zeta }\}_{\zeta >0}$ and an $\{\mathfrak {P}_{0}\}$ constituted all the elements with criterion zero.

The stochastic DE in $\mathfrak {d}$-dimensions is presented below:

2.3 $$\begin{aligned} d\textbf{v}(\zeta )=\textbf{u}(\textbf{v}(\zeta ),\zeta )d\zeta +\textbf{q}(\textbf{v}(\zeta ),\zeta )d\mathcal {W}(\zeta ),~\textbf{v}(\zeta _{0}) =\textbf{v}_{0},~\forall ~\zeta _{0}\le \zeta \le \textbf{T}<\infty , \end{aligned}$$

where $\textbf{u}:{\mathbb {R}}^{\mathfrak {d}}\times [\zeta _{0},\textbf{T}]\mapsto {\mathbb {R}}^{\mathfrak {d}}$ and $\textbf{q}:{\mathbb {R}}^{\mathfrak {d}}\times [\zeta _{0},\textbf{T}]\mapsto {\mathbb {R}}^{\mathfrak {d}\times m_{1}}$ are Borel measurable having $\mathcal {W}=\{\mathcal {W}(\zeta )\}_{\zeta \ge \zeta _{0}}$ is an ${\mathbb {R}}^{m_{1}}$-valued Wiener process, and $\textbf{v}_{0}$ is an ${\mathbb {R}}^{\mathfrak {d}}$-valued random variable presented as $\Theta .$

Therefore, ${\mathbb {C}}^{2,1}({\mathbb {R}}^{\mathfrak {d}}\times [\zeta _{0},\infty );{\mathbb {R}}_{+})$ is considered as the family of all non-negative functions $\mathcal {V}(\textbf{v},\zeta )$ on ${\mathbb {R}}^{\mathfrak {d}}\times [\zeta _{0},\infty )$ that are continuously twice differentiable in $\textbf{v}\in {\mathbb {R}}^{\mathfrak {d}}$ and once in $\zeta \in [\zeta _{0},\infty )$. The differential formulation ${\mathbb {L}}$ for the stochastic DE (2.3) is given as

$$\begin{aligned} {\mathbb {L}}=\frac{\partial }{\partial \zeta }+\sum \limits _{\varsigma =1}^{\mathfrak {d}} \textbf{u}_{\varsigma }(\textbf{v},\zeta )\frac{\partial }{\partial \textbf{v}_{\varsigma }} +\frac{1}{2}\sum \limits _{\textbf{i},\varsigma =1}^{\mathfrak {d}}\sum \limits _{\ell =1}^{m_{1}} {\textbf{q}}_{\varsigma \ell }(\textbf{v},\zeta ){\textbf{q}}_{\varsigma \ell }(\textbf{v},\zeta ) \frac{\partial ^{2}}{\partial \textbf{v}_{\varsigma }\partial \textbf{v}_{\textbf{i}}}. \end{aligned}$$

Introducing the functional $\mathcal {V}\in {\mathbb {C}}^{2,1}({\mathbb {R}}^{\mathfrak {d}}\times [\zeta _{0},\infty ),$ then

$$\begin{aligned} {\mathbb {L}}\mathcal {V}(\textbf{v},\zeta )=\mathcal {V}_{\zeta }(\textbf{v},\zeta )+\mathcal {V}_{\textbf{v}} (\textbf{v},\zeta )\textbf{f}(\textbf{v},\zeta )+\frac{1}{2}\sum \limits _{\textbf{i},\varsigma =1}^{\mathfrak {d}} \sum \limits _{\ell =1}^{m_{1}}{\textbf{q}}_{\textbf{i}\ell }(\textbf{v},\zeta ){\textbf{g}}_{\varsigma \ell }(\textbf{v},\zeta ) \mathcal {V}_{\textbf{v}\textbf{v}}(\textbf{v},\zeta ), \end{aligned}$$

where $$\mathcal {V}_{\zeta }:=\frac{\partial \mathcal {V}}{\partial \zeta };~\mathcal {V}_{\textbf{s}_{1}} =(\mathcal {V}_{\textbf{v}_{\varsigma }},...,\mathcal {V}_{\textbf{v}_{\mathfrak {d}}}), ~~\mathcal {V}_{\textbf{v}\textbf{v}}=(\mathcal {V}_{\textbf{v}_{\varsigma }}, \mathcal {V}_{\textbf{v}_{\varsigma }})_{\mathfrak {d}\times \mathfrak {d}}.$$

For $\textbf{v}(\zeta )\in {\mathbb {R}}^{\mathfrak {d}},$ then Itô’s method can be described as:

$$\begin{aligned} d\mathcal {V}(\textbf{v}(\zeta ),\zeta )={\mathbb {L}}\mathcal {V}(\textbf{v}(\zeta ),\zeta ) d\zeta +\mathcal {V}_{\textbf{v}}(\textbf{v}(\zeta ),\zeta ){\textbf{q}}(\textbf{v}(\zeta ),\zeta )d\mathcal {W}(\zeta ). \end{aligned}$$

Here, we furnish the associated overview here to assist viewers who are familiar with FC (see;^25–27^).

### **Definition 2.1**

(^25^) The Caputo fractional derivative of order $\Lambda$ for a continuous function $\mathcal {G}$ is defined by

$$\begin{aligned} {}\,^{C}\textbf{D}^{\Lambda } \mathcal {G}(\zeta )=\frac{1}{\Gamma (n-\Lambda )}\int \limits _{0}^{\zeta }\mathcal {G}^{\prime } (\textbf{w})(\zeta -\textbf{w})^{n-\Lambda -1}d\textbf{w},~~\big (n=\big [\Lambda \big ]+1\big ). \end{aligned}$$

Our second notion is a fractional derivative without singular kernel introduced by Caputo and Fabrizio^26^.

### **Definition 2.2**

(^26^) Let $b_{1}>0,~u_{1}\in H_{1}(a_{1},b_{1}),~and~\Lambda \in (0,1).$ The Caputo-Fabrizio derivative of order $\Lambda$ for a function $\mathcal {G}$ is defined by

$$\begin{aligned} \,^{CF}\textbf{D}^{\Lambda } \mathcal {G}(\zeta )=\frac{(2-\Lambda )\bar{\mathcal {M}}(\Lambda )}{2(1-\Lambda )}\int \limits _{0}^{\zeta } \mathcal {G}^{\prime }(\textbf{w})\exp \bigg [-\frac{\Lambda }{1-\Lambda }(\zeta -\textbf{w})\bigg ]d\textbf{w},~~\Lambda \in (0,1], \end{aligned}$$

where $\bar{\mathcal {M}}(\Lambda )=\frac{2}{2-\Lambda }$ is stated to be normalized mapping with $\bar{\mathcal {M}}(0)=\bar{\mathcal {M}}(1)=1.$

### **Definition 2.3**

(^27^) Let $b_{1}>0,~\mathcal {G}\in H_{1}(a_{1},b_{1}),~and~\Lambda \in (0,1).$ The ABC derivative of order $\Lambda$ for a function $\mathcal {G}$ is defined by

$$\begin{aligned} \,^{ABC}\textbf{D}_{\zeta }^{\Lambda } \mathcal {G}(\zeta )=\frac{ABC(\Lambda )}{1-\Lambda }\int \limits _{0}^{\zeta } \mathcal {G}^{\prime }(\textbf{w})E_{\Lambda }\bigg [-\frac{\Lambda }{1-\Lambda }(\zeta -\textbf{w})^{\Lambda }\bigg ] d\textbf{w},~~\Lambda \in (0,1], \end{aligned}$$

where $ABC(\Lambda )=1-\Lambda +\frac{\Lambda }{\Gamma (\Lambda )}$ is the normalization function satisfying $ABC(0)=ABC(1)=1$ and $E_{\Lambda }$ represents the one-parameter Mittag-Leffler function.

## Qualitative aspects of proposed model

Shah et al.^40^ researched the global features of the nonlinear malnutrition model, explaining how boys and girls relocate from one cohort to another as described in (2.1). For the sake of simplicity, we denote $\bar{X}=(\mathcal {S}_{\textbf{f}},\mathcal {M}_{\textbf{b}},\mathcal {M}_{\textbf{g}},\mathcal {U}).$

For framework (2.1), there is always a feasible region as follows:

3.1 $$\begin{aligned} \Theta :=\Big \{\bar{X}=(\mathcal {S}_{\textbf{f}},\mathcal {M}_{\textbf{b}},\mathcal {M}_{\textbf{g}}, \mathcal {U})\in {\mathbb {R}}_{+}^{4}:0\le \bar{X}\le \textbf{N}\le \frac{\mathcal {B}+\varepsilon }{\vartheta }\Big \}. \end{aligned}$$

Thus, the malnutrition steady state $\mathcal {E}_{0}=\Big (\frac{\mathcal {B}+\varepsilon }{\vartheta },0,0,0\Big ).$

Now, using the next-generation matrix approach^42^, compute the basic reproduction number ${\mathbb {R}}_{0}$. The next-generation matrix can be described as $\mathcal {F}\mathcal {V}^{-1}$, where $\mathcal {F}$ and $\mathcal {V}$ are both Jacobian matrices of individuals in an experimental setting is presented as follows:

$$\begin{aligned} \mathcal {F}_{\mathcal {E}_{0}}= \begin{pmatrix} \frac{\lambda _{\textbf{b}}(\mathcal {B}+\epsilon )}{\vartheta }&{}0&{}0&{}0\\ 0&{}\frac{\lambda _{\textbf{g}}(\mathcal {B}+\epsilon )}{\vartheta }&{}0&{}0\\ 0&{}0&{}0&{}0\\ 0&{}0&{}0&{}0 \end{pmatrix}~~~and~~~\mathcal {V}_{\mathcal {E}_{0}}=\begin{pmatrix} \gamma _{\textbf{b}}+\vartheta _{\textbf{b}}+\vartheta &{}0&{}-\chi _{\textbf{b}}&{}0\\ 0&{}\gamma _{\textbf{g}}+\vartheta +\delta _{\textbf{g}}&{}-\chi _{\textbf{g}}&{}0\\ -\gamma _{\textbf{b}}&{}-\gamma _{\textbf{g}}&{}\chi _{\textbf{b}}+\chi _{\textbf{g}}+\vartheta &{}0\\ \frac{\lambda _{\textbf{b}}}{\vartheta }(\mathcal {B}+\epsilon ) &{}\frac{\lambda _{\textbf{g}}}{\vartheta }(\mathcal {B}+\epsilon )-\delta _{\textbf{g}}&{}0&{}\vartheta \end{pmatrix}. \end{aligned}$$

Therefore, the basic reproduction number ${\mathbb {R}}_{0}$ is the spectral radius of matrix $\mathcal {F}\mathcal {V}^{-1}$ which is presented by

$$\begin{aligned} {\mathbb {R}}_{0}=\frac{\lambda _{\textbf{g}}(\mathcal {B}+\varepsilon )((\vartheta +\vartheta _{\textbf{b}})(\vartheta +\chi _{\textbf{b}}+\chi _{\textbf{g}}) +\gamma _{\textbf{b}}(\vartheta +\chi _{\textbf{g}}))}{\vartheta (\vartheta +\vartheta _{\textbf{b}})(\vartheta (\vartheta +\gamma _{\textbf{g}} +\delta _{\textbf{g}}+\chi _{\textbf{b}}+\chi _{\textbf{g}}) +\chi _{\textbf{b}}(\gamma _{\textbf{g}}+\delta _{\textbf{g}}) +\chi _{\textbf{g}}\delta _{\textbf{g}})}. \end{aligned}$$

Furthermore, the global behaviour of approach (2.1) is essentially depend on the fundamental reproduction number ${\mathbb {R}}_{0}.$

- If ${\mathbb {R}}_{0}\le 1$, then $\mathcal {E}_{0}=\Big (\frac{\mathcal {B}+\varepsilon }{\vartheta },0,0,0\Big )$ is globally asymptotically stable (GAS) in $\Theta$.
- If ${\mathbb {R}}_{0}>1$, then $\mathcal {E}_{1}=\Big ({\mathcal {S}_{\textbf{f}}}_{0}^{*},\bar{\mathcal {M}}_{\textbf{b}}^{*}, \bar{\mathcal {M}}_{\textbf{g}}^{*},\bar{\mathcal {U}}^{*}\Big )$ is GAS in $\Theta$.

### Stochastic analysis

Initially, we formulate the respective underlying formalism in terms of a unique global non-negative stochastic system solution (2.2).

#### **Theorem 3.1**

*Assume there is initial setting* $\bar{X}(0)\in {\mathbb {R}}_{+}^{4},$ *there exists a unique solution* $\bar{X}(\zeta )\in {\mathbb {R}}_{+}^{4}$ *of system (*2.2*) on* $\zeta \ge 0$ *and the outcome will stay in* ${\mathbb {R}}_{+}^{4}$ *with unit probability.*

#### *Proof*

It should be remarked that such framework (2.2) parameters are locally Lipschitz continuous, which means that for any specified initial settings $(\bar{X}(0))\in {\mathbb {R}}_{+}^{4},$ there is a unique maximal solution $\bar{X}(\zeta )$ on $\zeta \in [0,\phi _{\varepsilon }),$ where $\phi _{\varepsilon }$ is the explosion time. Assume that $\ell _{0}$ be sufficiently large such that $\bar{X}(0)$ lies in $[1/\ell _{0},\ell _{0}].$ For every integer $\ell \ge \ell _{0},$ specify the stopping time

$$\begin{aligned} \phi _{\ell }=\inf \Big \{\zeta \in [0,\phi _{\varepsilon }):\mathcal {S}_{\textbf{f}}(\zeta ) \ne \Big (\frac{1}{\ell },\ell \Big ),~\bar{\mathcal {M}}_{\textbf{b}}(\zeta )\ne \Big (\frac{1}{\ell },\ell \Big ), \bar{\mathcal {M}}_{\textbf{g}}(\zeta )\ne \Big (\frac{1}{\ell },\ell \Big ),\bar{\mathcal {U}}(\zeta ) \ne \Big (\frac{1}{\ell },\ell \Big )\Big \}. \end{aligned}$$

It is obvious that $\phi _{\ell }$ is increasing as $\ell \mapsto \infty .$ We acquire $\phi _{\infty }=\lim \limits _{\ell \mapsto \infty }\phi _{\ell },$ whenever $\phi _{\infty }\le \phi _{\varepsilon }~(a.s).$ To demonstrate a local global solution $\bar{X}(\zeta ),$ we just require to confirm $\phi _{\infty }=\infty ~(a.s).$ Therefore, two positive constants values $\varepsilon$ from (0, 1) and $\textbf{T}$ must exist, such that

3.2 $$\begin{aligned} \mathcal {P}\big \{\textbf{T}\ge \phi _{\infty }\big \}>\varepsilon . \end{aligned}$$

Consequently, the integer $\ell _{1}\ge \ell _{0}$ exists in the subsequent way

$$\begin{aligned} \mathcal {P}\big \{\textbf{T}\ge \phi _{\ell }\big \}\ge \varepsilon ,~~\forall ~\ell _{1}\le \ell . \end{aligned}$$

Introducing the non-negative ${\mathbb {C}}^{2}$-Lyapunov mapping as follows:

3.3 $$\begin{aligned} \mathcal {V}_{1}(\bar{X}) & = \big (\mathcal {S}_{\textbf{f}}-1-\ln {\mathcal {S}_{\textbf{f}}}\big ) +(\bar{\mathcal {M}}_{\textbf{b}}-1-\ln \bar{\mathcal {M}}_{\textbf{b}}) +\big (\bar{\mathcal {M}}_{\textbf{g}}-1-\ln {\bar{\mathcal {M}}_{\textbf{g}}}\big )\nonumber \\ & \quad +(\bar{\mathcal {U}}-1-\ln \bar{\mathcal {U}}). \end{aligned}$$

The inequality $\varkappa -1-\ln {\varkappa }\ge 0$ for $\varkappa >0$ can be used to calculate $\mathcal {V}_{1}$’s positivity.

Utilizing the Itô’s technique^43^ to $\mathcal {V}_{1},$ we have

$$\begin{aligned} d\mathcal {V}_{1}(\bar{X}) & = \mathcal {L}\mathcal {V}_{1}(\bar{X})d\zeta +\sigma _{1}(\mathcal {S}_{\textbf{f}}-1) d\mathcal {W}_{1}(\zeta )+\sigma _{2}(\bar{\mathcal {M}}_{\textbf{b}}-1)d\mathcal {W}_{2}(\zeta )\nonumber \\ & \quad +\sigma _{3}(\bar{\mathcal {M}}_{\textbf{g}}-1)d\mathcal {W}_{3}(\zeta ) +\sigma _{4}(\bar{\mathcal {U}}-1)d\mathcal {W}_{4}(\zeta ), \end{aligned}$$

In (3.3), $\mathcal {L}\mathcal {V}_{1}:{\mathbb {R}}_{+}^{4}\mapsto {\mathbb {R}}_{+}$ is described as

$$\begin{aligned} \mathcal {L}\mathcal {V}_{1} & = \Big (1-\frac{1}{\mathcal {S}_{\textbf{f}}}\Big )\Big \{(\mathcal {B}+\varepsilon ) -(\lambda _{\textbf{b}}\bar{\mathcal {M}}_{\textbf{b}}+\lambda _{\textbf{g}}\bar{\mathcal {M}}_{\textbf{g}} +\vartheta )\mathcal {S}_{\textbf{f}}+\delta _{\textbf{g}}\bar{\mathcal {M}}_{\textbf{g}}\Big \}\nonumber \\ & \quad + \Big (1-\frac{1}{\bar{\mathcal {M}}_{\textbf{g}}}\Big )\Big \{\lambda _{\textbf{g}}\mathcal {S}_{\textbf{f}} \bar{\mathcal {M}}_{\textbf{g}}-(\gamma _{\textbf{g}}+\delta _{\textbf{g}}+\vartheta )\bar{\mathcal {M}}_{\textbf{g}} +\chi _{\textbf{g}}\bar{\mathcal {U}}\Big \}\nonumber \\ & \quad + \Big (1-\frac{1}{\bar{\mathcal {M}}_{\textbf{b}}}\Big )\Big \{\lambda _{\textbf{b}}\mathcal {S}_{\textbf{f}} \bar{\mathcal {M}}_{\textbf{b}}-(\vartheta _{\textbf{b}}+\gamma _{\textbf{b}}+\vartheta )\bar{\mathcal {M}}_{\textbf{b}} +\chi _{\textbf{b}}\bar{\mathcal {U}}\Big \}\nonumber \\ & \quad +\Big (1-\frac{1}{\bar{\mathcal {U}}}\Big ) \Big \{\gamma _{\textbf{b}}\bar{\mathcal {M}}_{\textbf{b}}+\gamma _{\textbf{g}}\bar{\mathcal {M}}_{\textbf{g}} -(\chi _{\textbf{b}}+\chi _{\textbf{g}}+\vartheta )\bar{\mathcal {U}}\Big \}+\frac{\sigma _{1}^{2}+\sigma _{2}^{2} +\sigma _{3}^{2}+\sigma _{4}^{2}}{2}\nonumber \\ & \le \mathcal {B}+\varepsilon +\gamma _{\textbf{g}}+\delta _{\textbf{g}}-\vartheta _{\textbf{b}} -\gamma _{\textbf{b}}+\chi _{\textbf{b}}+\chi _{\textbf{g}}+\vartheta +\frac{\sigma _{1}^{2}+\sigma _{2}^{2} +\sigma _{3}^{2}+\sigma _{4}^{2}}{2}=:\mathcal {K}_{1}. \end{aligned}$$

Therefore, we have

$$\begin{aligned} & {\mathbb {U}}\Big [\mathcal {V}_{1}\big (\mathcal {S}_{\textbf{f}}(\phi _{\ell }\wedge \textbf{T})\big ), \big (\bar{\mathcal {M}}_{\textbf{b}}(\phi _{\ell }\wedge \textbf{T})\big ), \big (\bar{\mathcal {M}}_{\textbf{g}}(\phi _{\ell }\wedge \textbf{T})\big ), \big (\bar{\mathcal {U}}(\phi _{\ell }\wedge \textbf{T})\big )\Big ]\nonumber \\ & \quad \le \mathcal {V}_{1}(\bar{X}(0))+{\mathbb {U}}\int _{0}^{\phi _{\ell }\wedge \textbf{T}}\mathcal {K}d\zeta \nonumber \\ & \quad \le \mathcal {V}_{1}(\bar{X}(0))+\mathcal {K}\textbf{T}. \end{aligned}$$

inserting $\tilde{\mho }_{\ell }=\{\phi _{\ell }\le \textbf{T}\}$ for $\ell \ge \ell _{1}$ and utilizing (3.2), $\mathcal {P}(\tilde{\mho }_{\ell })\ge \varepsilon$. Observe that for every $\omega$ from $\tilde{\mho }_{\ell }$ there exists at least one $\bar{X}(\phi _{\ell },\omega )$ which yields $\frac{1}{\ell }~or~\ell .$

 Finally, $\mathcal {V}_{1}\big (\bar{X}(\phi _{\ell })\big )$ is no less than $\frac{1}{\ell }-1+\log \ell ~or~\ell -1-\log \ell .$ Thus

3.4 $$\begin{aligned} \mathcal {V}_{1}\big (\bar{X}(\phi _{\ell })\big ) \ge \Big (\frac{1}{\ell }-1+\log \ell \Big )\wedge {\mathbb {U}}(\ell -1-\log \ell ). \end{aligned}$$

In view of (3.2) and (3.4), we express

3.5 $$\begin{aligned} \mathcal {V}_{1}(\bar{X}(0))+\mathcal {K}\textbf{T}\ge & {} {\mathbb {U}} \Big \{\mathcal {I}_{\tilde{\mho }(\omega )}\mathcal {V}_{1}\big (\bar{X}(\phi _{\ell })\big )\Big \}\nonumber \\\ge & {} \varepsilon \Big \{\Big (\frac{1}{\ell }-1+\log \ell \Big )\wedge {\mathbb {U}}(\ell -1-\log \ell )\Big \}. \end{aligned}$$

where $\mathcal {I}_{\tilde{\mho }(\omega )}$ is the indicator mapping of $\tilde{\mho }$. Applying $\ell \mapsto \infty$ leads to the contradiction

3.6 $$\begin{aligned} \infty >\mathcal {V}_{1}(\bar{X}(0))+\mathcal {K}\textbf{T}=\infty , \end{aligned}$$

which implies that $\phi _{\infty }=\infty ,~(a.s)$ and this concludes the evidence. $\square$

### Existence of ergodic stationary distribution

When an ailment arrives in a community and begins to grow rapidly, health authorities are notably interested in its protracted behaviour, which can be efficaciously dealt with mathematically by incorporating stability techniques. In terms of deterministic modelling techniques, it is possible to demonstrate that in specific settings, the accompanying framework has an endemic equilibrium that is globally asymptotically stable. However, there is no endemic equilibrium in stochastic structures such as model (2.1), making it difficult to predict when the disorder will persist in communities. Depending on Has’minskii’s^41^ concept, we aim to demonstrate in this portion that framework (2.1) has an ESD, indicating that the ailment will endure. If we assume that $\sigma _{1}=\sigma _{2}=\sigma _{3}=\sigma _{4}=0,$ we can conveniently procure a deterministic preview of scheme (2.1); nevertheless, the stochastic model is remarkably distinct from its corresponding deterministic one. It is also understood that there is no endemic disorder state in the stochastic framework. As a result, the linear stability explanation cannot be used to investigate the disease’s perseverance. As a result, we focused on the envisaged system’s stationary distribution (2.2), which assumes that the concern will persist. Assume that the function $\textbf{X} (\zeta )$ is a regular time-homogeneous Markov process ${\mathbb {R}}_{+}^{n_{1}}$ with the mathematical version

3.7 $$\begin{aligned} d \textbf{X}(\zeta )=\textbf{b}(\textbf{X})d\zeta +\sum \limits _{\textbf{w}=1}^{\kappa } \delta _{\textbf{w}}d\mathcal {W}_{\textbf{w}}(\zeta ). \end{aligned}$$

The diffusion matrix is as shown in:

3.8 $$\begin{aligned} \mathcal {A}(\textbf{X})=[a_{\Bbbk \jmath }(\varkappa )],~~a_{\Bbbk \jmath }(\varkappa ) =\sum \limits _{\textbf{w}=1}^{\kappa }\delta _{\textbf{w}}^{\Bbbk }(\varkappa )\delta _{\textbf{w}}^{\jmath }(\varkappa ). \end{aligned}$$

#### **Lemma 3.2**

(^41^) *Suppose there is a Markov technique* $\textbf{X}(\zeta )$ *admits a unique stationary distribution* $\pi (.)$ *if there is a bounded region* $\bar{\mathcal {U}}\in {\mathbb {R}}^{d}$ *having a regular boundary such that its closure* $\bar{\mathcal {U}}\in {\mathbb {R}}^{d}$ *has the subsequent criterion:*

($$M_{1}$$)
*The smallest eigenvalue of the diffusion matrix* $\mathcal {A}(\zeta )$ *is very close to zero in the open region* $\bar{\mathcal {U}}$ *and some of its neighbours.*
($$M_{2}$$)
*For* $\varkappa \in {\mathbb {R}}^{d}\bar{\mathcal {U}},$ *the mean time it takes for a path emanating from* $\varkappa$ *to approach the set* $\bar{\mathcal {U}}$ *is finite, and* $\sup _{\varkappa \in \kappa }{\mathbb {E}}\phi _{\varkappa }<\infty$ *for each compact subset. Moreover, if* $\textbf{f}(.)$ *is an integrable mapping with regard to the measure* $\pi (.)$*, then*
  3.9 $$\begin{aligned} \mathcal {P}\Big \{\lim \limits _{\textbf{T}\mapsto \infty }\frac{1}{\textbf{T}} \int \limits _{0}^{\textbf{T}}\textbf{f}({\textbf{X}}_{\varkappa }(\zeta ))d\zeta =\int \limits _{{\mathbb {R}}^{d_{1}}}\textbf{f}(\varkappa )\pi (d\varkappa )\Big \}=1. \end{aligned}$$

Now let’s classify some other threshold significance for future needs:

3.10 $$\begin{aligned} {\mathbb {R}}_{0}^{s}=\frac{\lambda _{\textbf{g}}\lambda _{\textbf{b}}}{\big (\lambda _{\textbf{b}} +\vartheta -\frac{\sigma _{1}^{2}}{2}\big ){\big (\vartheta _{\textbf{b}}+\gamma _{\textbf{b}} -\frac{\sigma _{2}^{2}}{2}\big )\big (\gamma _{\textbf{g}}+\delta _{\textbf{g}}+\vartheta -\frac{\sigma _{3}^{2}}{2}\big )}}. \end{aligned}$$

#### **Theorem 3.3**

*For* ${\mathbb {R}}_{0}^{s}>1,$ *then the model (*2.2*)* $\bar{X}(\zeta )$ *is ergodic. Moreover, there is a unique stationary distribution* $\pi (.).$

#### *Proof*

First, we should indeed illustrate the design specifications $M_{1}$ of Lemma 3.2 to validate the Theorem, we assert a positive ${\mathbb {C}}^{2}$-mapping $H_{1}:{\mathbb {R}}_{+}^{4}\mapsto {\mathbb {R}}_{+}$ in the frame of

3.11 $$\begin{aligned} H_{1}=\mathcal {S}_{\textbf{f}}+\bar{\mathcal {M}}_{\textbf{b}}+\bar{\mathcal {M}}_{\textbf{g}} +\bar{\mathcal {U}}-\psi _{1}\ln \mathcal {S}_{\textbf{f}}-\psi _{2}\ln \bar{\mathcal {M}}_{\textbf{b}} -\psi _{3}\ln \bar{\mathcal {M}}_{\textbf{g}}. \end{aligned}$$

The positive components must be determined later in this case. These specifications must be found out later on. To communicate directly with (3.11), we first should apply Itô’s approach to the design process (2.2) as

3.12 $$\begin{aligned} \mathcal {L}(\mathcal {S}_{\textbf{f}}+\bar{\mathcal {M}}_{\textbf{b}} +\bar{\mathcal {M}}_{\textbf{g}}+\bar{\mathcal {U}})=(\mathcal {B}+\varepsilon ) -\vartheta \textbf{N}-\vartheta _{\textbf{b}}\bar{\mathcal {M}}_{\textbf{b}}. \end{aligned}$$

As a result of this,

3.13 $$\begin{aligned} \mathcal {L}(-\ln \mathcal {S}_{\textbf{f}}) & =-\frac{\mathcal {B}+\varepsilon }{\mathcal {S}_{\textbf{f}}} +(\lambda _{\textbf{b}}+\lambda _{\textbf{g}}\bar{\mathcal {M}}_{\textbf{g}}+\vartheta ) -\frac{\delta _{\textbf{g}}\bar{\mathcal {M}}_{\textbf{g}}}{\mathcal {S}_{\textbf{f}}} -\frac{\sigma _{1}^{2}}{2},\nonumber \\ \mathcal {L}(-\ln \bar{\mathcal {M}}_{\textbf{b}}) & =-\lambda _{\textbf{b}}{\mathcal {S}_{\textbf{f}}} +(\vartheta _{\textbf{b}}+\gamma _{\textbf{b}}+\vartheta )\bar{\mathcal {M}}_{\textbf{b}} -\frac{\chi _{\textbf{b}}}{\bar{\mathcal {U}}}-\frac{\sigma _{2}^{2}}{2},\nonumber \\ \mathcal {L}(-\ln \bar{\mathcal {M}}_{\textbf{g}}) & =-\lambda _{\textbf{g}}{\mathcal {S}_{\textbf{f}}} +(\gamma _{\textbf{g}}+\delta _{\textbf{g}}+\vartheta )\bar{\mathcal {M}}_{\textbf{g}} -\frac{\chi _{\textbf{g}}}{\bar{\mathcal {U}}}-\frac{\sigma _{3}^{2}}{2},\nonumber \\ \mathcal {L}(-\ln \bar{\mathcal {U}}) &=-\frac{\gamma _{\textbf{b}}{\bar{\mathcal {M}}_{\textbf{b}}}}{\bar{\mathcal {U}}}-\frac{\gamma _{\textbf{g}}\bar{\mathcal {M}}_{\textbf{g}}}{\bar{\mathcal {U}}} -(\chi _{\textbf{b}}+\chi _{\textbf{g}}+\vartheta )-\frac{\sigma _{4}^{2}}{2}. \end{aligned}$$

Further, we express

3.14 $$\begin{aligned} \mathcal {L}(H_{1})=\mathcal {L}(\mathcal {S}_{\textbf{f}}+\bar{\mathcal {M}}_{\textbf{b}} +\bar{\mathcal {M}}_{\textbf{g}}+\bar{\mathcal {U}})-\psi _{1}\mathcal {L}(\ln \mathcal {S}_{\textbf{f}}) -\psi _{2}\mathcal {L}(\ln \bar{\mathcal {M}}_{\textbf{b}})-\psi _{3}\mathcal {L}(\ln \bar{\mathcal {M}}_{\textbf{g}}). \end{aligned}$$

After plugging the values into the upcoming equation, produces

$$\begin{aligned} \mathcal {L}(H_{1}) & = (\mathcal {B}+\varepsilon )-\vartheta \textbf{N}-\vartheta _{\textbf{b}} \bar{\mathcal {M}}_{\textbf{b}}+\psi _{1}\frac{\mathcal {B}+\varepsilon }{\mathcal {S}_{\textbf{f}}} -\psi _{1}(\lambda _{\textbf{b}}+\lambda _{\textbf{g}}\bar{\mathcal {M}}_{\textbf{g}}+\vartheta ) +\psi _{1}\frac{\delta _{\textbf{g}}\bar{\mathcal {M}}_{\textbf{g}}}{\mathcal {S}_{\textbf{f}}} +\psi _{1}\frac{\sigma _{1}^{2}}{2}+\psi _{2}\lambda _{\textbf{b}}{\mathcal {S}_{\textbf{f}}}\\ & \quad -\psi _{2}(\vartheta _{\textbf{b}}+\gamma _{\textbf{b}}+\vartheta ) \bar{\mathcal {M}}_{\textbf{b}}+\psi _{2}\frac{\chi _{\textbf{b}}}{\bar{\mathcal {U}}} +\psi _{2}\frac{\sigma _{2}^{2}}{2}+\psi _{3}\lambda _{\textbf{g}}{\mathcal {S}_{\textbf{f}}} -\psi _{3}(\gamma _{\textbf{g}}+\delta _{\textbf{g}}+\vartheta )\bar{\mathcal {M}}_{\textbf{g}} +\psi _{3}\frac{\chi _{\textbf{g}}}{\bar{\mathcal {U}}}+\psi _{3}\frac{\sigma _{3}^{2}}{2}\\ & \le -4\Big (\psi _{1}\frac{\mathcal {B}+\varepsilon }{\mathcal {S}_{\textbf{f}}}\psi _{3} \lambda _{\textbf{g}}\mathcal {S}_{\textbf{f}}\psi _{2}\lambda _{\textbf{b}}\Big )^{1/4}-\psi _{2} (\vartheta _{\textbf{b}}+\gamma _{\textbf{b}})-\psi _{1}(\lambda _{\textbf{b}}+\vartheta ) -\psi _{3}(\gamma _{\textbf{g}}+\delta _{\textbf{g}}+\vartheta )+(\mathcal {B}+\varepsilon )\\ & \quad +\psi _{1}\frac{\delta _{\textbf{g}}\bar{\mathcal {M}}_{\textbf{g}}}{\mathcal {S}_{\textbf{f}}} +\psi _{2}\Big (\frac{\chi _{\textbf{b}}}{\bar{\mathcal {U}}}-(\vartheta _{\textbf{b}}+\gamma _{\textbf{b}+\vartheta })\Big ) +\psi _{3}\Big (\frac{\chi _{\textbf{g}}}{\bar{\mathcal {U}}}-(\gamma _{\textbf{g}}+\delta _{\textbf{g}}+\vartheta )\Big ) +\psi _{1}\frac{\sigma _{1}^{2}}{2}+\psi _{2}\frac{\sigma _{2}^{2}}{2}+\psi _{3}\frac{\sigma _{3}^{2}}{2}\\ & =-4\Big (\psi _{1}{(\mathcal {B}+\varepsilon )}\psi _{3}\lambda _{\textbf{g}}\psi _{2}\lambda _{\textbf{b}}\Big )^{1/4} -\psi _{2}(\vartheta _{\textbf{b}}+\gamma _{\textbf{b}})-\psi _{1}(\lambda _{\textbf{b}}+\vartheta ) -\psi _{3}(\gamma _{\textbf{g}}+\delta _{\textbf{g}}+\vartheta )+(\mathcal {B}+\varepsilon )\\ & \quad +\psi _{1}\frac{\delta _{\textbf{g}}\bar{\mathcal {M}}_{\textbf{g}}}{\mathcal {S}_{\textbf{f}}} +\psi _{2}\Big (\frac{\chi _{\textbf{b}}}{\bar{\mathcal {U}}}-(\vartheta _{\textbf{b}}+\gamma _{\textbf{b}+\vartheta })\Big ) +\psi _{3}\Big (\frac{\chi _{\textbf{g}}}{\bar{\mathcal {U}}}-(\gamma _{\textbf{g}}+\delta _{\textbf{g}} +\vartheta )\Big )+\psi _{1}\frac{\sigma _{1}^{2}}{2}+\psi _{2}\frac{\sigma _{2}^{2}}{2}+\psi _{3}\frac{\sigma _{3}^{2}}{2}. \end{aligned}$$

Now, we suppose that

$$\begin{aligned} \mathcal {B}+\varepsilon =\psi _{1}(\lambda _{\textbf{b}}+\vartheta -\frac{\sigma _{1}^{2}}{2}) =\psi _{2}(\vartheta _{\textbf{b}}+\gamma _{\textbf{b}}-\frac{\sigma _{2}^{2}}{2}) =\psi _{3}(\gamma _{\textbf{g}}+\delta _{\textbf{g}}+\vartheta -\frac{\sigma _{3}^{2}}{2}), \end{aligned}$$

where

$$\begin{aligned} \psi _{1}=\frac{\mathcal {B}+\varepsilon }{\big (\lambda _{\textbf{b}}+\vartheta -\frac{\sigma _{1}^{2}}{2}\big )},~\psi _{2}=\frac{\mathcal {B}+\varepsilon }{\big (\vartheta _{\textbf{b}}+\gamma _{\textbf{b}}-\frac{\sigma _{2}^{2}}{2}\big )}, ~~\psi _{3}=\frac{\mathcal {B}+\varepsilon }{\big (\gamma _{\textbf{g}}+\delta _{\textbf{g}} +\vartheta -\frac{\sigma _{3}^{2}}{2}\big )}. \end{aligned}$$

Consequently, we have

3.15 $$\begin{aligned} \mathcal {L}(H_{1}) & \le -4\Big (\frac{(\mathcal {B}+\varepsilon )^{4}\lambda _{\textbf{g}} \lambda _{\textbf{b}}}{\big (\lambda _{\textbf{b}}+\vartheta -\frac{\sigma _{1}^{2}}{2}\big ) {\big (\vartheta _{\textbf{b}}+\gamma _{\textbf{b}}-\frac{\sigma _{2}^{2}}{2}\big )\big (\gamma _{\textbf{g}} +\delta _{\textbf{g}}+\vartheta -\frac{\sigma _{3}^{2}}{2}\big )}} -4(\mathcal {B}+\varepsilon )^{4}\Big )^{1/4}\nonumber \\ & \quad +\psi _{1}\frac{\delta _{\textbf{g}}\bar{\mathcal {M}}_{\textbf{g}}}{\mathcal {S}_{\textbf{f}}} +\psi _{2}\Big (\frac{\chi _{\textbf{b}}}{\bar{\mathcal {U}}}-(\vartheta _{\textbf{b}} +\gamma _{\textbf{b}+\vartheta })\Big )+\psi _{3}\Big (\frac{\chi _{\textbf{g}}}{\bar{\mathcal {U}}} -(\gamma _{\textbf{g}}+\delta _{\textbf{g}}+\vartheta )\Big )\nonumber \\ & \le -4(\mathcal {B}+\varepsilon )\big [\big (\textbf{R}_{0}^{s}\big )^{1/4}-1\big ]+\psi _{1} \frac{\delta _{\textbf{g}}\bar{\mathcal {M}}_{\textbf{g}}}{\mathcal {S}_{\textbf{f}}}. \end{aligned}$$

Furthermore, one can achieve that

$$\begin{aligned} H_{2} & =\psi _{4}(\mathcal {S}_{\textbf{f}}+\bar{\mathcal {M}}_{\textbf{b}}+\bar{\mathcal {M}}_{\textbf{g}} +\bar{\mathcal {U}}-\psi _{1}\ln \mathcal {S}_{\textbf{f}}-\psi _{2}\ln \bar{\mathcal {M}}_{\textbf{b}}-\psi _{3} \ln \bar{\mathcal {M}}_{\textbf{g}})-\ln \mathcal {S}_{\textbf{f}}-\ln \bar{\mathcal {U}} -\ln \bar{\mathcal {M}}_{\textbf{g}}\\ & \quad +\mathcal {S}_{\textbf{f}} +\bar{\mathcal {M}}_{\textbf{b}}+\bar{\mathcal {M}}_{\textbf{g}}+\bar{\mathcal {U}}\\ & =(\psi _{4}+1)(\mathcal {S}_{\textbf{f}}+\bar{\mathcal {M}}_{\textbf{b}}+\bar{\mathcal {M}}_{\textbf{g}} +\bar{\mathcal {U}})-(\psi _{1}\psi _{4}+1)\ln \mathcal {S}_{\textbf{f}}-\psi _{4}\psi _{2}\ln \bar{\mathcal {M}}_{\textbf{b}} -\ln \bar{\mathcal {U}}-\psi _{4}\psi _{3}\ln \bar{\mathcal {M}}_{\textbf{g}}, \end{aligned}$$

here, $\psi _{4}>0$ is a fixed value that will be discovered later. It is critical to illustrate that

$$\begin{aligned} \lim \inf \limits _{(\bar{X})\in {\mathbb {R}}_{+}^{4}\setminus \bar{\mathcal {U}}_{\kappa }} H_{2}(\bar{X})=+\infty ,~as~\kappa \mapsto \infty , \end{aligned}$$

here $\bar{\mathcal {U}}_{\kappa }=(\frac{1}{\kappa },\kappa )\times (\frac{1}{\kappa },\kappa ) \times (\frac{1}{\kappa },\kappa )\times (\frac{1}{\kappa },\kappa ).$ The following procedure will demonstrate that $H_{2}(\bar{X})$ has a least value $H_{2}(\bar{X}(0))$.

The partial derivative of $H_{2}(\bar{X})$ regarding to $\bar{X}$ is as follows

3.16 $$\begin{aligned} \frac{\partial H_{2}(\bar{X})}{\partial \mathcal {S}_{\textbf{f}}} &=1+\psi _{4} -\frac{1+\psi _{1}\psi _{4}}{\mathcal {S}_{\textbf{f}}},\nonumber \\ \frac{\partial H_{2}(\bar{X})}{\partial \bar{\mathcal {M}}_{\textbf{b}}} &=1+\psi _{4} -\frac{\psi _{2}\psi _{4}}{\bar{\mathcal {M}}_{\textbf{b}}},\nonumber \\ \frac{\partial H_{2}(\bar{X})}{\partial \bar{\mathcal {M}}_{\textbf{g}}}& =1+\psi _{4}-\frac{\psi _{3}\psi _{4}}{\bar{\mathcal {M}}_{\textbf{g}}},\nonumber \\ \frac{\partial H_{2}(\bar{X})}{\partial \bar{\mathcal {U}}} &=1+\psi _{4}-\frac{1}{\bar{\mathcal {U}}}. \end{aligned}$$

It is straightforward to show that $H_{2}$ has a distinctive stagnation point, which seems ascertained by the aforementioned computation:

3.17 $$\begin{aligned} \big (\bar{X}\big )=\Big (\frac{1+\psi _{1}\psi _{4}}{1+C_{4}},\frac{\psi _{2}\psi _{4}}{1+\psi _{4}}, \frac{\psi _{2}\psi _{4}}{1+\psi _{4}},\frac{1}{1+\psi _{4}}\Big ). \end{aligned}$$

Also, the Hessian matrix of $H_{2}\big (\bar{X}\big )$ at $\big (\bar{X}(0)\big )$ is presented by the following

3.18 $$\begin{aligned} \mathcal {B}=\begin{bmatrix} \frac{1+\psi _{1}\psi _{4}}{\mathcal {S}_{\textbf{f}}^{2}}&{}0&{}0&{}0\\ 0&{}\frac{\psi _{2}\psi _{4}}{\bar{\mathcal {M}}_{\textbf{b}}^{2}}&{}0&{}0\\ 0&{}0&{}\frac{\psi _{3}\psi _{4}}{\bar{\mathcal {M}}_{\textbf{g}}^{2}}&{}0\\ 0&{}0&{}0&{}\frac{1}{\bar{\mathcal {U}}^{2}} \end{bmatrix}. \end{aligned}$$

The preceding link demonstrates unequivocally that $\mathcal {B}$ is a non-negative definite matrix. Thus, $H_{2}(\bar{X})$ has minimum value $(\bar{X}(0)).$ Finally, Lemma 3.2 concludes and the continuity of $H_{2}(\bar{X})$ that it has a distinct lowest value of about $(\bar{X}(0))$ in the interior of ${\mathbb {R}}_{+}^{4}.$ Further, we define a positive ${\mathbb {C}}^{2}:{\mathbb {R}}_{+}^{4}\mapsto {\mathbb {R}}_{+}$ as follow

3.19 $$\begin{aligned} H_{1}(\bar{X})=H_{2}(\bar{X})-H_{2}(\bar{X}(0)). \end{aligned}$$

The application of Itô’s strategy and the structure (2.2) will give us

3.20 $$\begin{aligned} \mathcal {L}H_{1} & \le \psi _{4}\Big \{-4(\mathcal {B}+\varepsilon )\big [({\mathbb {R}}_{0}^{s})^{1/4}-1\big ] +\psi _{1}\frac{\delta _{\textbf{g}}\bar{\mathcal {M}}_{\textbf{g}}}{\mathcal {S}_{\textbf{f}}}\Big \} -\frac{\mathcal {B}+\varepsilon }{\mathcal {S}_{\textbf{f}}}+(\lambda _{\textbf{b}} +\lambda _{\textbf{g}}\bar{\mathcal {M}}_{\textbf{g}}+\vartheta )-\frac{\delta _{\textbf{g}} \bar{\mathcal {M}}_{\textbf{g}}}{\mathcal {S}_{\textbf{f}}}-\frac{\sigma _{1}^{2}}{2}\nonumber \\ & \quad -\lambda _{\textbf{b}}{\mathcal {S}_{\textbf{f}}}+(\vartheta _{\textbf{b}}+\gamma _{\textbf{b}}+\vartheta ) \bar{\mathcal {M}}_{\textbf{b}}-\frac{\chi _{\textbf{b}}}{\bar{\mathcal {U}}}-\frac{\sigma _{2}^{2}}{2} -\lambda _{\textbf{g}}{\mathcal {S}_{\textbf{f}}}+(\gamma _{\textbf{g}}+\delta _{\textbf{g}}+\vartheta ) \bar{\mathcal {M}}_{\textbf{g}}-\frac{\chi _{\textbf{g}}}{\bar{\mathcal {U}}}-\frac{\sigma _{3}^{2}}{2}\nonumber \\ & \quad -\frac{\gamma _{\textbf{b}}{\bar{\mathcal {M}}_{\textbf{b}}}}{\bar{\mathcal {U}}}-\frac{\gamma _{\textbf{g}} \bar{\mathcal {M}}_{\textbf{g}}}{\bar{\mathcal {U}}}-(\chi _{\textbf{b}}+\chi _{\textbf{g}}+\vartheta )-\frac{\sigma _{4}^{2}}{2}, \end{aligned}$$

or finally we can express

3.21 $$\begin{aligned} \mathcal {L}H_{1} & \le -\psi _{4}\psi _{5}+(\psi _{1}\psi _{4}-1)\frac{\delta _{\textbf{g}} \bar{\mathcal {M}}_{\textbf{g}}}{\mathcal {S}_{\textbf{f}}} -\frac{\mathcal {B}+\varepsilon }{\mathcal {S}_{\textbf{f}}}+(\lambda _{\textbf{b}} -\chi _{\textbf{b}}-\chi _{\textbf{g}})\nonumber \\ & \quad -(\lambda _{\textbf{b}} +\lambda _{\textbf{g}}){\mathcal {S}_{\textbf{f}}}+(\vartheta _{\textbf{b}} +\gamma _{\textbf{b}}+\vartheta )\bar{\mathcal {M}}_{\textbf{b}}-\frac{\chi _{\textbf{b}} +\chi _{\textbf{g}}}{\bar{\mathcal {U}}}+(\gamma _{\textbf{g}}+\delta _{\textbf{g}}+\vartheta +\lambda _{\textbf{g}}) \bar{\mathcal {M}}_{\textbf{g}}\nonumber \\ & \quad -\frac{\gamma _{\textbf{b}}{\bar{\mathcal {M}}_{\textbf{b}}} +\gamma _{\textbf{g}}\bar{\mathcal {M}}_{\textbf{g}}}{\bar{\mathcal {U}}}-\frac{\sigma _{1}^{2}\vee \sigma _{2}^{2} \vee \sigma _{3}^{2}\vee \sigma _{4}^{2}}{2}, \end{aligned}$$

where $$\psi _{5}=4(\mathcal {B}+\varepsilon )\big [({\mathbb {R}}_{0}^{s})^{1/4}-1\big ]>0.$$

The representation of a collection is supplied by

3.22 $$\begin{aligned} \mathcal {Y}=\big \{\mathcal {S}_{\textbf{f}}\in \big [\varepsilon _{1},\frac{1}{\varepsilon _{2}}\big ], \bar{\mathcal {M}}_{\textbf{b}}\in \big [\varepsilon _{1},\frac{1}{\varepsilon _{2}}\big ],\bar{\mathcal {M}}_{\textbf{g}} \in \big [\varepsilon _{1},\frac{1}{\varepsilon _{2}}\big ],\bar{\mathcal {U}} \in \big [\varepsilon _{1},\frac{1}{\varepsilon _{2}}\big ]\Big \}, \end{aligned}$$

where $\varepsilon _{\Bbbk },~\Bbbk =1,2$, are fixed which are extremely small and will have to be revealed afterward. The domain ${\mathbb {R}}_{+}^{4}\setminus \mathcal {Y}$ is separated into ten zones, which are as follows:

3.23 $$\begin{aligned} \mathcal {Y}_{1} &=\Big \{\bar{X}\in {\mathbb {R}}_{+}^{4},0<\mathcal {S}_{\textbf{f}}\le \varepsilon _{1}\Big \},\nonumber \\ \mathcal {Y}_{2} &=\Big \{\bar{X}\in {\mathbb {R}}_{+}^{4},0<\bar{\mathcal {M}}_{\textbf{b}} \le \varepsilon _{2},~\mathcal {S}_{\textbf{f}}>\varepsilon _{2}\Big \},\nonumber \\ \mathcal {Y}_{3}& =\Big \{\bar{X}\in {\mathbb {R}}_{+}^{4},0<\bar{\mathcal {M}}_{\textbf{g}} \le \varepsilon _{1},~\bar{\mathcal {M}}_{\textbf{b}}>\varepsilon _{2}\Big \},\nonumber \\ \mathcal {Y}_{4} &=\Big \{\bar{X}\in {\mathbb {R}}_{+}^{4},0<\bar{\mathcal {U}} \le \varepsilon _{1},~\bar{\mathcal {M}}_{\textbf{g}}>\varepsilon _{2}\Big \},\nonumber \\ \mathcal {Y}_{5} &=\Big \{\bar{X}\in {\mathbb {R}}_{+}^{4},\mathcal {S}_{\textbf{f}}\ge \frac{1}{\varepsilon _{2}}\Big \},\nonumber \\ \mathcal {Y}_{6} &=\Big \{\bar{X}\in {\mathbb {R}}_{+}^{4},\bar{\mathcal {M}}_{\textbf{b}}\ge \frac{1}{\varepsilon _{2}}\Big \},\nonumber \\ \mathcal {Y}_{7} &=\Big \{\bar{X}\in {\mathbb {R}}_{+}^{4},\bar{\mathcal {M}}_{\textbf{g}}\ge \frac{1}{\varepsilon _{2}}\Big \},\nonumber \\ \mathcal {Y}_{8} &=\Big \{\bar{X}\in {\mathbb {R}}_{+}^{4},\bar{\mathcal {U}}\ge \frac{1}{\varepsilon _{2}}\Big \}. \end{aligned}$$

Clearly, ${\mathbb {R}}_{+}^{4}\setminus \mathcal {Y}=\bigcup _{\Bbbk =1}^{8}\mathcal {Y}_{\Bbbk },~\Bbbk =1,...,8.$ Finally, we will investigate $H_{1}(\bar{X})$ for each $\bar{X}\in {\mathbb {R}}_{+}^{4}\setminus \mathcal {Y}$. As a result of (3.21), it is not difficult to figure out that

$$\begin{aligned} \mathcal {L}H_{1}(\bar{X}), ~for~ \bar{X}\in {\mathbb {R}}_{+}^{4}\setminus \mathcal {Y} =\bigcup _{\Bbbk =1}^{8}\mathcal {Y}_{\Bbbk },~\Bbbk =1,...,8. \end{aligned}$$

**Case I.** If $\bar{X}\in \mathcal {Y}_{1},$ then by (3.21), we have

$$\begin{aligned} \mathcal {L}H_{1} & \le -\psi _{4}\psi _{5}+(\psi _{1}\psi _{4}-1)\frac{\delta _{\textbf{g}} \bar{\mathcal {M}}_{\textbf{g}}}{\mathcal {S}_{\textbf{f}}}-\frac{\mathcal {B}+\varepsilon }{\mathcal {S}_{\textbf{f}}} +(\lambda _{\textbf{b}}-\chi _{\textbf{b}}-\chi _{\textbf{g}})\nonumber \\ & \quad -(\lambda _{\textbf{b}}+\lambda _{\textbf{g}}){\mathcal {S}_{\textbf{f}}} +(\vartheta _{\textbf{b}}+\gamma _{\textbf{b}}+\vartheta ) \bar{\mathcal {M}}_{\textbf{b}}-\frac{\chi _{\textbf{b}}+\chi _{\textbf{g}}}{\bar{\mathcal {U}}} +(\gamma _{\textbf{g}}+\delta _{\textbf{g}}+\vartheta +\lambda _{\textbf{g}})\bar{\mathcal {M}}_{\textbf{g}}\nonumber \\ & \quad -\frac{\gamma _{\textbf{b}}{\bar{\mathcal {M}}_{\textbf{b}}} +\gamma _{\textbf{g}}\bar{\mathcal {M}}_{\textbf{g}}}{\bar{\mathcal {U}}} -\frac{\sigma _{1}^{2}\vee \sigma _{2}^{2}\vee \sigma _{3}^{2}\vee \sigma _{4}^{2}}{2}\nonumber \\ & \le -\psi _{4}\psi _{5}+(\psi _{2}\psi _{4}+1)\frac{\delta _{\textbf{g}}}{\varepsilon _{1}} -\frac{\mathcal {B}+\varepsilon }{\varepsilon _{1}}\le -1. \end{aligned}$$

**Case II.** If $\bar{X}\in \mathcal {Y}_{2},$ then by (3.21), we have

$$\begin{aligned} \mathcal {L}H_{1} & \le -\psi _{4}\psi _{5}+(\psi _{1}\psi _{4}-1)\frac{\delta _{\textbf{g}}\bar{\mathcal {M}}_{\textbf{g}}}{\mathcal {S}_{\textbf{f}}}-\frac{\mathcal {B}+\varepsilon }{\mathcal {S}_{\textbf{f}}}+(\lambda _{\textbf{b}} -\chi _{\textbf{b}}-\chi _{\textbf{g}})\nonumber \\ & \quad -(\lambda _{\textbf{b}}+\lambda _{\textbf{g}}) {\mathcal {S}_{\textbf{f}}}+(\vartheta _{\textbf{b}}+\gamma _{\textbf{b}}+\vartheta ) \bar{\mathcal {M}}_{\textbf{b}}-\frac{\chi _{\textbf{b}}+\chi _{\textbf{g}}}{\bar{\mathcal {U}}} +(\gamma _{\textbf{g}}+\delta _{\textbf{g}}+\vartheta +\lambda _{\textbf{g}})\bar{\mathcal {M}}_{\textbf{g}}\nonumber \\ & \quad -\frac{\gamma _{\textbf{b}}{\bar{\mathcal {M}}_{\textbf{b}}}+\gamma _{\textbf{g}} \bar{\mathcal {M}}_{\textbf{g}}}{\bar{\mathcal {U}}} -\frac{\sigma _{1}^{2}\vee \sigma _{2}^{2}\vee \sigma _{3}^{2}\vee \sigma _{4}^{2}}{2}\nonumber \\ &\le -\psi _{4}\psi _{5}+(\psi _{2}\psi _{4}+1)\frac{\delta _{\textbf{g}}}{\varepsilon _{1}} -{\gamma _{\textbf{b}}}{\varepsilon _{2}}\le -1. \end{aligned}$$

**Case III.** If $\bar{X}\in \mathcal {Y}_{3},$ then by (3.21), we have

$$\begin{aligned} \mathcal {L}H_{1} & \le -\psi _{4}\psi _{5}+(\psi _{1}\psi _{4}-1)\frac{\delta _{\textbf{g}} \bar{\mathcal {M}}_{\textbf{g}}}{\mathcal {S}_{\textbf{f}}}-\frac{\mathcal {B}+\varepsilon }{\mathcal {S}_{\textbf{f}}} +(\lambda _{\textbf{b}}-\chi _{\textbf{b}}-\chi _{\textbf{g}})\nonumber \\ & \quad -(\lambda _{\textbf{b}} +\lambda _{\textbf{g}}){\mathcal {S}_{\textbf{f}}}+(\vartheta _{\textbf{b}}+\gamma _{\textbf{b}}+\vartheta ) \bar{\mathcal {M}}_{\textbf{b}}-\frac{\chi _{\textbf{b}}+\chi _{\textbf{g}}}{\bar{\mathcal {U}}}+(\gamma _{\textbf{g}} +\delta _{\textbf{g}}+\vartheta +\lambda _{\textbf{g}})\bar{\mathcal {M}}_{\textbf{g}}\nonumber \\ & \quad -\frac{\gamma _{\textbf{b}}{\bar{\mathcal {M}}_{\textbf{b}}}+\gamma _{\textbf{g}} \bar{\mathcal {M}}_{\textbf{g}}}{\bar{\mathcal {U}}}-\frac{\sigma _{1}^{2}\vee \sigma _{2}^{2} \vee \sigma _{3}^{2}\vee \sigma _{4}^{2}}{2}\nonumber \\ & \le -\psi _{4}\psi _{5}-(\gamma _{\textbf{b}}\varepsilon _{2}+\gamma _{\textbf{g}}\varepsilon _{1})\le -1. \end{aligned}$$

**Case IV.** If $\bar{X}\in \mathcal {Y}_{4},$ then by (3.21), we have

$$\begin{aligned} \mathcal {L}H_{1} & \le -\psi _{4}\psi _{5}+(\psi _{1}\psi _{4}-1) \frac{\delta _{\textbf{g}}\bar{\mathcal {M}}_{\textbf{g}}}{\mathcal {S}_{\textbf{f}}} -\frac{\mathcal {B}+\varepsilon }{\mathcal {S}_{\textbf{f}}}+(\lambda _{\textbf{b}} -\chi _{\textbf{b}}-\chi _{\textbf{g}})\nonumber \\ & \quad -(\lambda _{\textbf{b}}+\lambda _{\textbf{g}}){\mathcal {S}_{\textbf{f}}}+(\vartheta _{\textbf{b}} +\gamma _{\textbf{b}}+\vartheta )\bar{\mathcal {M}}_{\textbf{b}}-\frac{\chi _{\textbf{b}} +\chi _{\textbf{g}}}{\bar{\mathcal {U}}}+(\gamma _{\textbf{g}}+\delta _{\textbf{g}}+\vartheta +\lambda _{\textbf{g}})\bar{\mathcal {M}}_{\textbf{g}}\nonumber \\ & \quad -\frac{\gamma _{\textbf{b}} {\bar{\mathcal {M}}_{\textbf{b}}}+\gamma _{\textbf{g}}\bar{\mathcal {M}}_{\textbf{g}}}{\bar{\mathcal {U}}} -\frac{\sigma _{1}^{2}\vee \sigma _{2}^{2}\vee \sigma _{3}^{2}\vee \sigma _{4}^{2}}{2}\nonumber \\ & \le -\psi _{4}\psi _{5}-\frac{\gamma _{\textbf{g}}\varepsilon _{2}}{\varepsilon _{1}}\le -1. \end{aligned}$$

**Case V.** If $\bar{X}\in \mathcal {Y}_{5},$ then by (3.21), we have

$$\begin{aligned} \mathcal {L}H_{1} & \le -\psi _{4}\psi _{5}+(\psi _{1}\psi _{4}-1)\frac{\delta _{\textbf{g}} \bar{\mathcal {M}}_{\textbf{g}}}{\mathcal {S}_{\textbf{f}}}-\frac{\mathcal {B}+\varepsilon }{\mathcal {S}_{\textbf{f}}} +(\lambda _{\textbf{b}}-\chi _{\textbf{b}}-\chi _{\textbf{g}})\nonumber \\ & \quad -(\lambda _{\textbf{b}}+\lambda _{\textbf{g}}){\mathcal {S}_{\textbf{f}}} +(\vartheta _{\textbf{b}}+\gamma _{\textbf{b}}+\vartheta )\bar{\mathcal {M}}_{\textbf{b}} -\frac{\chi _{\textbf{b}}+\chi _{\textbf{g}}}{\bar{\mathcal {U}}}+(\gamma _{\textbf{g}}+\delta _{\textbf{g}} +\vartheta +\lambda _{\textbf{g}})\bar{\mathcal {M}}_{\textbf{g}}\nonumber \\ & \quad -\frac{\gamma _{\textbf{b}}{\bar{\mathcal {M}}_{\textbf{b}}}+\gamma _{\textbf{g}} \bar{\mathcal {M}}_{\textbf{g}}}{\bar{\mathcal {U}}}-\frac{\sigma _{1}^{2}\vee \sigma _{2}^{2} \vee \sigma _{3}^{2}\vee \sigma _{4}^{2}}{2}\nonumber \\ & \le -\psi _{4}\psi _{5}-\frac{\delta _{\textbf{g}}}{\varepsilon _{2}}\le -1. \end{aligned}$$

**Case VI.** If $\bar{X}\in \mathcal {Y}_{6},$ then by (3.21), we have

$$\begin{aligned} \mathcal {L}H_{1} & \le -\psi _{4}\psi _{5}+(\psi _{1}\psi _{4}-1)\frac{\delta _{\textbf{g}} \bar{\mathcal {M}}_{\textbf{g}}}{\mathcal {S}_{\textbf{f}}}-\frac{\mathcal {B}+\varepsilon }{\mathcal {S}_{\textbf{f}}} +(\lambda _{\textbf{b}}-\chi _{\textbf{b}}-\chi _{\textbf{g}})\nonumber \\ & \quad -(\lambda _{\textbf{b}} +\lambda _{\textbf{g}}){\mathcal {S}_{\textbf{f}}}+(\vartheta _{\textbf{b}} +\gamma _{\textbf{b}}+\vartheta ) \bar{\mathcal {M}}_{\textbf{b}}-\frac{\chi _{\textbf{b}}+\chi _{\textbf{g}}}{\bar{\mathcal {U}}} +(\gamma _{\textbf{g}}+\delta _{\textbf{g}}+\vartheta +\lambda _{\textbf{g}})\bar{\mathcal {M}}_{\textbf{g}}\nonumber \\ & \quad -\frac{\gamma _{\textbf{b}}{\bar{\mathcal {M}}_{\textbf{b}}}+\gamma _{\textbf{g}}\bar{\mathcal {M}}_{\textbf{g}}}{\bar{\mathcal {U}}}-\frac{\sigma _{1}^{2}\vee \sigma _{2}^{2}\vee \sigma _{3}^{2}\vee \sigma _{4}^{2}}{2}\nonumber \\ & \le -\psi _{4}\psi _{5}+\frac{(\vartheta _{\textbf{b}}+\gamma _{\textbf{b}}+\vartheta )}{\varepsilon _{2}} -\frac{\gamma _{\textbf{b}}}{\varepsilon _{2}}\le -1. \end{aligned}$$

**Case VII.** If $\bar{X}\in \mathcal {Y}_{7},$ then by (3.21), we have

$$\begin{aligned} \mathcal {L}H_{1} & \le -\psi _{4}\psi _{5}+(\psi _{1}\psi _{4}-1)\frac{\delta _{\textbf{g}}\bar{\mathcal {M}}_{\textbf{g}}}{\mathcal {S}_{\textbf{f}}}-\frac{\mathcal {B}+\varepsilon }{\mathcal {S}_{\textbf{f}}}+(\lambda _{\textbf{b}} -\chi _{\textbf{b}}-\chi _{\textbf{g}})\nonumber \\ & \quad -(\lambda _{\textbf{b}}+\lambda _{\textbf{g}}){\mathcal {S}_{\textbf{f}}}+(\vartheta _{\textbf{b}} +\gamma _{\textbf{b}}+\vartheta )\bar{\mathcal {M}}_{\textbf{b}}-\frac{\chi _{\textbf{b}} +\chi _{\textbf{g}}}{\bar{\mathcal {U}}}+(\gamma _{\textbf{g}}+\delta _{\textbf{g}}+\vartheta +\lambda _{\textbf{g}})\bar{\mathcal {M}}_{\textbf{g}}\nonumber \\ & \quad -\frac{\gamma _{\textbf{b}}{\bar{\mathcal {M}}_{\textbf{b}}}+\gamma _{\textbf{g}}\bar{\mathcal {M}}_{\textbf{g}}}{\bar{\mathcal {U}}}-\frac{\sigma _{1}^{2}\vee \sigma _{2}^{2}\vee \sigma _{3}^{2}\vee \sigma _{4}^{2}}{2}\nonumber \\ & \le -\psi _{4}\psi _{5}+\frac{(\gamma _{\textbf{g}}+\delta _{\textbf{g}}+\vartheta +\lambda _{\textbf{g}}+\vartheta )}{\varepsilon _{2}}-\frac{\gamma _{\textbf{g}}}{\varepsilon _{2}}\le -1. \end{aligned}$$

**Case VIII.** If $\bar{X}\in \mathcal {Y}_{8},$ then by (3.21), we have

$$\begin{aligned} \mathcal {L}H_{1} & \le -\psi _{4}\psi _{5}+(\psi _{1}\psi _{4}-1)\frac{\delta _{\textbf{g}} \bar{\mathcal {M}}_{\textbf{g}}}{\mathcal {S}_{\textbf{f}}}-\frac{\mathcal {B}+\varepsilon }{\mathcal {S}_{\textbf{f}}} +(\lambda _{\textbf{b}}-\chi _{\textbf{b}}-\chi _{\textbf{g}})\nonumber \\ & \quad -(\lambda _{\textbf{b}}+\lambda _{\textbf{g}}){\mathcal {S}_{\textbf{f}}}+(\vartheta _{\textbf{b}} +\gamma _{\textbf{b}}+\vartheta )\bar{\mathcal {M}}_{\textbf{b}}-\frac{\chi _{\textbf{b}} +\chi _{\textbf{g}}}{\bar{\mathcal {U}}}+(\gamma _{\textbf{g}}+\delta _{\textbf{g}}+\vartheta +\lambda _{\textbf{g}})\bar{\mathcal {M}}_{\textbf{g}}\nonumber \\ & \quad -\frac{\gamma _{\textbf{b}}{\bar{\mathcal {M}}_{\textbf{b}}} +\gamma _{\textbf{g}}\bar{\mathcal {M}}_{\textbf{g}}}{\bar{\mathcal {U}}} -\frac{\sigma _{1}^{2}\vee \sigma _{2}^{2}\vee \sigma _{3}^{2}\vee \sigma _{4}^{2}}{2}\nonumber \\ & \le -\psi _{4}\psi _{5}-\frac{(\chi _{\textbf{g}}+\chi _{\textbf{b}})}{\varepsilon _{2}}\le -1. \end{aligned}$$

Finally, all of the previous contexts demonstrate that a non-negative $\mathcal {B}$ exists, so $\mathcal {L}H_{1}(\bar{X})<-\mathcal {B}<0~\forall ~(\bar{X})\in {\mathbb {R}}_{+}^{4}\setminus \mathcal {Y}.$ Hence

3.24 $$\begin{aligned} dH_{1}(\bar{X})< & {} -\mathcal {B}d\zeta +\big [(\psi _{4}+1)\mathcal {S}_{\textbf{f}}-(\psi _{1}\psi _{4}+1) \sigma _{1}\big ]d\mathcal {W}_{1}(\zeta )\nonumber \\ {}{} & {} \quad + \big [(\psi _{4}+1)\bar{\mathcal {M}}_{\textbf{b}}-\psi _{1}\psi _{4}\sigma _{2}\big ]d\mathcal {W}_{2}(\zeta ) +\big [(\psi _{4}+1)\bar{\mathcal {M}}_{\textbf{g}}-\psi _{3}\psi _{4}\sigma _{3}\big ]d\mathcal {W}_{3}(\zeta )\nonumber \\{} & {} \quad +\big [(\psi _{4}+1)\bar{\mathcal {U}}-\sigma _{4}\big ]d\mathcal {W}_{4}(\zeta ). \end{aligned}$$

Suppose $(\bar{X})=(u_{1},u_{2},u_{3},u_{4},u_{5})=\bar{u}\in {\mathbb {R}}_{+}^{4}\setminus \mathcal {Y},$ the time $\phi ^{\bar{u}},$ where a path starting with $\bar{u}$ led directly to the collection $\mathcal {Y},$ $\phi ^{n_{1}}=\inf \{\zeta :\vert \textbf{X}(\zeta )\vert =n_{1}\}$ and $\phi ^{n}(\zeta )=\min \{\phi _{\bar{u}},\zeta ,\phi ^{n_{1}}\}.$ Performing integration on (3.24) over 0 to $\phi ^{(n_{1})}(\zeta ),$ applying expectation and Dynkins process, we conclude that

3.25 $$\begin{aligned}{} & {} {\mathbb {E}}H_{1}\big (\mathcal {S}_{\textbf{f}}(\phi ^{(n_{1})(\zeta )}), \bar{\mathcal {M}}_{\mathcal {B}}(\phi ^{(n_{1})(\zeta )}),\bar{\mathcal {M}}_{G_{1}} (\phi ^{(n_{1})(\zeta )}),\bar{\mathcal {U}}(\phi ^{(n_{1})(\zeta )})\big )-H_{1}(\bar{u})\nonumber \\ & = {\mathbb {E}}\int \limits _{0}^{\phi ^{(n_{1})}(\zeta )}H_{1}\big (\mathcal {S}_{\textbf{f}}(u_{1}), \bar{\mathcal {M}}_{\mathcal {B}}(u_{1}),\bar{\mathcal {M}}_{G_{1}}(u_{1}),\bar{\mathcal {U}}(u_{1})\big )du_{1}\nonumber \\ & \le {\mathbb {E}}\int \limits _{0}^{\phi ^{(n_{1})}(\zeta )}-\mathcal {B}du_{1} =-\mathcal {B}{\mathbb {E}}\phi ^{(n_{1})}(\zeta ). \end{aligned}$$

As $H(\bar{u})$ is positive, thus

3.26 $$\begin{aligned} {\mathbb {E}}\phi ^{(n_{1})}(\zeta )\le \frac{H_{1}(\bar{u})}{\mathcal {B}}. \end{aligned}$$

So, $\mathcal {P}\{\phi _{\varepsilon }=\infty \}=1$ and We can affirm that the proposed methodology (2.2) is correct. We require to apply Fatou’s well-known lemma as

3.27 $$\begin{aligned} {\mathbb {E}}\phi ^{(n_{1})}(\zeta )\le \frac{H_{1}(\bar{u})}{\mathcal {B}}<\infty . \end{aligned}$$

Evidently, $\sup _{\bar{u}\in \mathcal {K}}{\mathbb {E}}\phi ^{\bar{u}}<\infty ,$ where $\mathcal {K}$ is a compact subset from ${\mathbb {R}}_{+}^{4}.$ As a direct consequence, Lemma 3.2’s second requirement is satisfied Also, the diffusion matrix of the framework (2.2) is

3.28 $$\begin{aligned} \mathcal {B}=\begin{bmatrix} {\sigma _{1}^{2}}{\mathcal {S}_{\textbf{f}}^{2}}&{}0&{}0&{}0\\ 0&{}{\sigma _{2}^{2}}{\bar{\mathcal {M}}_{\textbf{b}}^{2}}&{}0&{}0\\ 0&{}0&{}{\sigma _{3}}^{2}{\bar{\mathcal {M}}_{\textbf{g}}^{2}}&{}0\\ 0&{}0&{}0&{}{\sigma _{4}^{2}}{\bar{\mathcal {U}}^{2}} \end{bmatrix}. \end{aligned}$$

Selecting $M_{1}=\min \limits _{(\bar{X})}\in \mathcal {Y}\in {\mathbb {R}}_{+}^{4}\big \{\sigma _{1}^{2} \mathcal {S}_{\textbf{f}}^{2},\sigma _{2}^{2}\bar{\mathcal {M}}_{\textbf{b}}^{2},\sigma _{3}^{2} \bar{\mathcal {M}}_{\textbf{g}}^{2},\sigma _{4}^{2}\bar{\mathcal {U}}^{2}\big \},$ we illustrate

$$\begin{aligned} \sum \limits _{\Bbbk ,\jmath =1}^{4}a_{\Bbbk \jmath }(\bar{X})\zeta _{\Bbbk }\zeta _{\jmath } =\sigma _{1}^{2}\mathcal {S}_{\textbf{f}}^{2}\zeta ^{2} +\sigma _{2}^{2}\bar{\mathcal {M}}_{\textbf{b}}^{2}\zeta ^{2} +\sigma _{3}^{2}\bar{\mathcal {M}}_{\textbf{g}}^{2}\zeta ^{2}+\sigma _{4}^{2}\bar{\mathcal {U}}^{2}\zeta ^{2}\ge M_{1}\vert \zeta \vert 
^{2},~~\bar{X}\in \bar{\mathcal {Y}}, \end{aligned}$$

where $$\zeta =(\zeta _{1},\zeta _{2},\zeta _{3},\zeta _{4})\in {\mathbb {R}}_{+}^{4}.$$

Thus, the $\mathcal {M_{1}}$ of Lemma 3.2 is fulfilled. The proposed stochastic structure has a unique ESD as an outcome of Lemma 3.2. $\square$

## Numerical simulation

In what follows, we will contemplate the numerical modelling using the power-law kernel, the exponential decay kernel and generalized Mittag-Leffler kernel, respectively.

### Power-law kernel

Here, we will examine at the nonlinear dynamics of poor nutrition systems (2.1) and (2.2) that incorporate malnutrition and underweight, using conventional, index-law and subsequently stochastic treatments. If we consider ${\mathbb {T}}$ to be the final time of dissemination, then the computational structure will be constructed during the initial process utilizing the classical derivative implementation, followed by the power-law kernel in the other approach and eventually the random perturbations in the later stages. The computational framework that accounts for this occurrence is then given as follows:

4.1 $$\begin{aligned} {\left\{ \begin{array}{ll} \frac{d\mathcal {S}_{\textbf{f}}}{d\zeta }=(\mathcal {B}+\varepsilon )-(\lambda _{\textbf{b}}\bar{\mathcal {M}}_{\textbf{b}}+\lambda _{\textbf{g}}\bar{\mathcal {M}}_{\textbf{g}}+\vartheta )\mathcal {S}_{\textbf{f}}+\delta _{\textbf{g}}\bar{\mathcal {M}}_{\textbf{g}},\\ \frac{d\bar{\mathcal {M}}_{\textbf{b}}}{d\zeta }=\lambda _{\textbf{b}}\mathcal {S}_{\textbf{f}}\bar{\mathcal {M}}_{\textbf{b}}-(\vartheta _{\textbf{b}}+\gamma _{\textbf{b}}+\vartheta )\bar{\mathcal {M}}_{\textbf{b}}+\chi _{\textbf{b}}\bar{\mathcal {U}},~if~0\le \zeta \le {\mathbb {T}}_{1},\\ \frac{d\bar{\mathcal {M}}_{\textbf{g}}}{d\zeta }=\lambda _{\textbf{g}}\mathcal {S}_{\textbf{f}}\bar{\mathcal {M}}_{\textbf{g}}-(\gamma _{\textbf{g}}+\delta _{\textbf{g}}+\vartheta )\bar{\mathcal {M}}_{\textbf{g}}+\chi _{\textbf{g}}\bar{\mathcal {U}},\\ \frac{d\bar{\mathcal {U}}}{d\zeta }=\gamma _{\textbf{b}}\bar{\mathcal {M}}_{\textbf{b}}+\gamma _{\textbf{g}}\bar{\mathcal {M}}_{\textbf{g}}-(\chi _{\textbf{b}}+\chi _{\textbf{g}}+\vartheta )\bar{\mathcal {U}},\end{array}\right. } \end{aligned}$$

4.2 $$\begin{aligned} {\left\{ \begin{array}{ll} \,_{0}^{c}\textbf{D}_{\zeta }^{\Lambda }{\mathcal {S}_{\textbf{f}}}=(\mathcal {B}+\varepsilon )-(\lambda _{\textbf{b}}\bar{\mathcal {M}}_{\textbf{b}}+\lambda _{\textbf{g}}\bar{\mathcal {M}}_{\textbf{g}}+\vartheta )\mathcal {S}_{\textbf{f}}+\delta _{\textbf{g}}\bar{\mathcal {M}}_{\textbf{g}},\\ \,_{0}^{c}\textbf{D}_{\zeta }^{\Lambda }{\bar{\mathcal {M}}_{\textbf{b}}}=\lambda _{\textbf{b}}\mathcal {S}_{\textbf{f}}\bar{\mathcal {M}}_{\textbf{b}}-(\vartheta _{\textbf{b}}+\gamma _{\textbf{b}}+\vartheta )\bar{\mathcal {M}}_{\textbf{b}}+\chi _{\textbf{b}}\bar{\mathcal {U}},~if~{\mathbb {T}}_{1}\le \zeta \le {\mathbb {T}}_{2},\\ \,_{0}^{c}\textbf{D}_{\zeta }^{\Lambda }{\bar{\mathcal {M}}_{\textbf{g}}}=\lambda _{\textbf{g}}\mathcal {S}_{\textbf{f}}\bar{\mathcal {M}}_{\textbf{g}}-(\gamma _{\textbf{g}}+\delta _{\textbf{g}}+\vartheta )\bar{\mathcal {M}}_{\textbf{g}}+\chi _{\textbf{g}}\bar{\mathcal {U}},\\ \,_{0}^{c}\textbf{D}_{\zeta }^{\Lambda }{\bar{\mathcal {U}}}=\gamma _{\textbf{b}}\bar{\mathcal {M}}_{\textbf{b}}+\gamma _{\textbf{g}}\bar{\mathcal {M}}_{\textbf{g}}-(\chi _{\textbf{b}}+\chi _{\textbf{g}}+\vartheta )\bar{\mathcal {U}},\end{array}\right. } \end{aligned}$$

4.3 $$\begin{aligned} {\left\{ \begin{array}{ll} d{\mathcal {S}_{\textbf{f}}}(\zeta )=\big ((\mathcal {B}+\varepsilon )-(\lambda _{\textbf{b}}\bar{\mathcal {M}}_{\textbf{b}}+\lambda _{\textbf{g}}\bar{\mathcal {M}}_{\textbf{g}}+\vartheta )\mathcal {S}_{\textbf{f}}+\delta _{\textbf{g}}\bar{\mathcal {M}}_{\textbf{g}}\big )+\sigma _{1}\mathcal {S}_{\textbf{f}}(\zeta )d\mathcal {W}_{1}(\zeta ),\\ d{\bar{\mathcal {M}}_{\textbf{b}}}(\zeta )=\big (\lambda _{\textbf{b}}\mathcal {S}_{\textbf{f}}\bar{\mathcal {M}}_{\textbf{b}}-(\vartheta _{\textbf{b}}+\gamma _{\textbf{b}}+\vartheta )\bar{\mathcal {M}}_{\textbf{b}}+\chi _{\textbf{b}}\bar{\mathcal {U}}\big )+\sigma _{2}\bar{\mathcal {M}}_{\textbf{b}}(\zeta )d\mathcal {W}_{2}(\zeta ),~if~{\mathbb {T}}_{2}\le \zeta \le {\mathbb {T}},\\ d{\bar{\mathcal {M}}_{\textbf{g}}}(\zeta )=\big (\lambda _{\textbf{g}}\mathcal {S}_{\textbf{f}}\bar{\mathcal {M}}_{\textbf{g}}-(\gamma _{\textbf{g}}+\delta _{\textbf{g}}+\vartheta )\bar{\mathcal {M}}_{\textbf{g}}+\chi _{\textbf{g}}\bar{\mathcal {U}}\big )+\sigma _{3}\bar{\mathcal {M}}_{\textbf{g}}(\zeta )d\mathcal {W}_{3}(\zeta ),\\ d{\bar{\mathcal {U}}}(\zeta )=\big (\gamma _{\textbf{b}}\bar{\mathcal {M}}_{\textbf{b}}+\gamma _{\textbf{g}}\bar{\mathcal {M}}_{\textbf{g}}-(\chi _{\textbf{b}}+\chi _{\textbf{g}}+\vartheta )\bar{\mathcal {U}}\big )+\sigma _{4}\bar{\mathcal {U}}(\zeta )d\mathcal {W}_{r}(\zeta ). \end{array}\right. } \end{aligned}$$

Here, we employ the method reported in^36^ for the situation of Caputo’s derivative to calculate and investigate the piecewise configuration (4.1)–(4.3). We begin the methodology by doing the following:

$$\begin{aligned} {\left\{ \begin{array}{ll} \frac{d\tilde{\mho }_{\ell }(\zeta )}{d\zeta }=\Phi (\zeta ,\tilde{\mho }_{\ell }).~\tilde{\mho }_{\ell }(0)=\tilde{\mho }_{\ell ,0},~\ell =1,2,...,\mathfrak {n}~if~\zeta \in [0,{\mathbb {T}}_{1}],\\ \,_{{\mathbb {T}}_{1}}^{c}\textbf{D}_{\zeta }^{\Lambda }\tilde{\mho }_{\ell }(\zeta )=\Phi (\zeta ,\tilde{\mho }_{\ell }),~\tilde{\mho }_{\ell }({\mathbb {T}}_{1})=\tilde{\mho }_{\ell ,1},~if~\zeta \in [{\mathbb {T}}_{1},{\mathbb {T}}_{2}],\\ d\tilde{\mho }_{\ell }(\zeta )=\Phi (\zeta ,\tilde{\mho }_{\ell })d\zeta +\wp _{\ell }\tilde{\mho }_{\ell }d\mathcal {W}_{\ell }(\zeta ),~\tilde{\mho }_{\ell }({\mathbb {T}}_{2})=\tilde{\mho }_{\ell ,2},~if~\zeta \in [{\mathbb {T}}_{2},{\mathbb {T}}]. \end{array}\right. } \end{aligned}$$

Accordingly, we have

$$\begin{aligned} \tilde{\mho }_{\ell }^{\textbf{w}}={\left\{ \begin{array}{ll}\tilde{\mho }_{\ell }(0)+\sum \limits _{\varsigma =2}^{\textbf{w}}\Big \{\frac{23}{12}\Phi ({\zeta }_{\varsigma },\tilde{\mho }^{\varsigma })\Delta \zeta -\frac{4}{3}\Phi ({\zeta }_{\varsigma -1},\tilde{\mho }^{\varsigma -1})\Delta \zeta +\frac{5}{12}\Phi ({\zeta }_{\varsigma -2},\tilde{\mho }^{\varsigma -2})\Delta \zeta \Big \},~~\zeta \in [0,\mathbb {T_{1}}].\\ \tilde{\mho }_{\ell }({\mathbb {T}}_{1})+\frac{(\Delta \zeta )^{\Lambda -1}}{\Gamma (\Lambda +1)}\sum \limits _{\varsigma =2}^{\textbf{w}}\Phi ({\zeta }_{\varsigma -2},\tilde{\mho }^{\varsigma -2})\Im _{1}\\ \quad + \frac{(\Delta \zeta )^{\Lambda -1}}{\Gamma (\Lambda +2)}\sum \limits _{\varsigma =2}^{\textbf{w}}\Big \{\Phi ({\zeta }_{\varsigma -1},\tilde{\mho }^{\varsigma -1})-\Phi ({\zeta }_{\varsigma -2},\tilde{\mho }^{\varsigma -2})\Big \}\Im _{2}\\ \quad +\frac{\Lambda (\Delta \zeta )^{\Lambda -1}}{2\Gamma (\Lambda +3)}\sum \limits _{\varsigma =2}^{\textbf{w}}\Big \{\Phi ({\zeta }_{\varsigma },\tilde{\mho }^{\varsigma })-2\Phi ({\zeta }_{\varsigma -1},\tilde{\mho }^{\varsigma -1})+\Phi ({\zeta }_{\varsigma -2},\tilde{\mho }^{\varsigma -2})\Big \}\Im _{3},~~\zeta \in [{\mathbb {T}}_{1},{\mathbb {T}}_{2}],\\ \tilde{\mho }_{\ell }({\mathbb {T}}_{2})+\sum \limits _{\varsigma =\textbf{w}+3}^{\mathfrak {n}}\Big \{\frac{5}{12}\Phi ({\zeta }_{\varsigma -2},\tilde{\mho }^{\varsigma -2})\Delta \zeta -\frac{4}{3}\Phi ({\zeta }_{\varsigma -1},\tilde{\mho }^{\varsigma -1})\Delta \zeta +\frac{23}{12}\Phi ({\zeta }_{\varsigma },\tilde{\mho }^{\varsigma })\Delta \zeta \Big \} \\ \quad +\sum \limits _{\varsigma =\textbf{w}+3}^{\mathfrak {n}}\Big \{\frac{5}{12}\big (\mathcal {W}({\zeta }_{\varsigma -1})-\mathcal {W}({\zeta }_{\varsigma -2})\big )\wp \tilde{\mho }^{\varsigma -2}- \frac{4}{3}\big (\mathcal {W}({\zeta }_{\varsigma })-\mathcal {W}({\zeta }_{\varsigma -1})\big )\wp \tilde{\mho }^{\varsigma -1}\\ \quad +\frac{23}{12}\big (\mathcal {W}({\zeta }_{\varsigma +1})-\mathcal {W}({\zeta }_{\varsigma })\big )\wp \tilde{\mho }^{\varsigma }\Big \},~~\zeta \in [{\mathbb {T}}_{2},{\mathbb {T}}],\end{array}\right. } \end{aligned}$$

where

4.4 $$\begin{aligned} \Im _{1}:=(\textbf{w}-\varsigma -1)^{\Lambda }-(\textbf{w}-\varsigma )^{\Lambda }, \end{aligned}$$

4.5 $$\begin{aligned} \Im _{2}:=(\textbf{w}-\varsigma +1)^{\Lambda }(\textbf{w}-\varsigma +2\Lambda +3) -(\textbf{w}-\varsigma )^{\Lambda }(\textbf{w}-\varsigma +3\Lambda +3) \end{aligned}$$

and

4.6 $$\begin{aligned} \Im _{3}:={\left\{ \begin{array}{ll}(\textbf{w}-\varsigma +1)^{\Lambda }\Big (2(\textbf{w}-\varsigma )^{2}+ (3\Lambda +10)(\textbf{w}-\varsigma )+2\Lambda ^{2}+9\Lambda +12\Big )\\ \quad + (\textbf{w}-\varsigma )^{\Lambda }\Big (2(\textbf{w}-\varsigma )^{2} +(5\Lambda +10)(\textbf{w}-\varsigma )+6\Lambda ^{2}+18\Lambda +12\Big ). \end{array}\right. } \end{aligned}$$

### Exponential decay kernel

In this segment, we will take a glance at the simulation framework of a poor nutrition framework that involves malnutrition and underweight congregation members, as well as conventional, exponential decay and random perturbations. If we define ${\mathbb {T}}$ as the ultimate dissemination duration, then the computational formation will be established during the initial phase that uses the integer-order derivative implementation, then comes the exponentially decaying kernel in the other phase, and finally the Gaussian noise in the future period. In this reference, the scientific model we are using to exemplify this incidence is as follows:

4.7 $$\begin{aligned} {\left\{ \begin{array}{ll} \frac{d\mathcal {S}_{\textbf{f}}}{d\zeta }=(\mathcal {B}+\varepsilon )-(\lambda _{\textbf{b}}\bar{\mathcal {M}}_{\textbf{b}}+\lambda _{\textbf{g}}\bar{\mathcal {M}}_{\textbf{g}}+\vartheta )\mathcal {S}_{\textbf{f}}+\delta _{\textbf{g}}\bar{\mathcal {M}}_{\textbf{g}},\\ \frac{d\bar{\mathcal {M}}_{\textbf{b}}}{d\zeta }=\lambda _{\textbf{b}}\mathcal {S}_{\textbf{f}}\bar{\mathcal {M}}_{\textbf{b}}-(\vartheta _{\textbf{b}}+\gamma _{\textbf{b}}+\vartheta )\bar{\mathcal {M}}_{\textbf{b}}+\chi _{\textbf{b}}\bar{\mathcal {U}},~if~0\le \zeta \le {\mathbb {T}}_{1},\\ \frac{d\bar{\mathcal {M}}_{\textbf{g}}}{d\zeta }=\lambda _{\textbf{g}}\mathcal {S}_{\textbf{f}}\bar{\mathcal {M}}_{\textbf{g}}-(\gamma _{\textbf{g}}+\delta _{\textbf{g}}+\vartheta )\bar{\mathcal {M}}_{\textbf{g}}+\chi _{\textbf{g}}\bar{\mathcal {U}},\\ \frac{d\bar{\mathcal {U}}}{d\zeta }=\gamma _{\textbf{b}}\bar{\mathcal {M}}_{\textbf{b}}+\gamma _{\textbf{g}}\bar{\mathcal {M}}_{\textbf{g}}-(\chi _{\textbf{b}}+\chi _{\textbf{g}}+\vartheta )\bar{\mathcal {U}},\end{array}\right. } \end{aligned}$$

4.8 $$\begin{aligned} {\left\{ \begin{array}{ll} \,_{0}^{CF}\textbf{D}_{\zeta }^{\Lambda }{\mathcal {S}_{\textbf{f}}}=(\mathcal {B}+\varepsilon )-(\lambda _{\textbf{b}}\bar{\mathcal {M}}_{\textbf{b}}+\lambda _{\textbf{g}}\bar{\mathcal {M}}_{\textbf{g}}+\vartheta )\mathcal {S}_{\textbf{f}}+\delta _{\textbf{g}}\bar{\mathcal {M}}_{\textbf{g}},\\ \,_{0}^{CF}\textbf{D}_{\zeta }^{\Lambda }{\bar{\mathcal {M}}_{\textbf{b}}}=\lambda _{\textbf{b}}\mathcal {S}_{\textbf{f}}\bar{\mathcal {M}}_{\textbf{b}}-(\vartheta _{\textbf{b}}+\gamma _{\textbf{b}}+\vartheta )\bar{\mathcal {M}}_{\textbf{b}}+\chi _{\textbf{b}}\bar{\mathcal {U}},~if~{\mathbb {T}}_{1}\le \zeta \le {\mathbb {T}}_{2},\\ \,_{0}^{CF}\textbf{D}_{\zeta }^{\Lambda }{\bar{\mathcal {M}}_{\textbf{g}}}=\lambda _{\textbf{g}}\mathcal {S}_{\textbf{f}}\bar{\mathcal {M}}_{\textbf{g}}-(\gamma _{\textbf{g}}+\delta _{\textbf{g}}+\vartheta )\bar{\mathcal {M}}_{\textbf{g}}+\chi _{\textbf{g}}\bar{\mathcal {U}},\\ \,_{0}^{CF}\textbf{D}_{\zeta }^{\Lambda }{\bar{\mathcal {U}}}=\gamma _{\textbf{b}}\bar{\mathcal {M}}_{\textbf{b}}+\gamma _{\textbf{g}}\bar{\mathcal {M}}_{\textbf{g}}-(\chi _{\textbf{b}}+\chi _{\textbf{g}}+\vartheta )\bar{\mathcal {U}},\end{array}\right. } \end{aligned}$$

4.9 $$\begin{aligned} {\left\{ \begin{array}{ll} d{\mathcal {S}_{\textbf{f}}}(\zeta )=\big ((\mathcal {B}+\varepsilon )-(\lambda _{\textbf{b}}\bar{\mathcal {M}}_{\textbf{b}}+\lambda _{\textbf{g}}\bar{\mathcal {M}}_{\textbf{g}}+\vartheta )\mathcal {S}_{\textbf{f}}+\delta _{\textbf{g}}\bar{\mathcal {M}}_{\textbf{g}}\big )+\sigma _{1}\mathcal {S}_{\textbf{f}}(\zeta )d\mathcal {W}_{1}(\zeta ),\\ d{\bar{\mathcal {M}}_{\textbf{b}}}(\zeta )=\big (\lambda _{\textbf{b}}\mathcal {S}_{\textbf{f}}\bar{\mathcal {M}}_{\textbf{b}}-(\vartheta _{\textbf{b}}+\gamma _{\textbf{b}}+\vartheta )\bar{\mathcal {M}}_{\textbf{b}}+\chi _{\textbf{b}}\bar{\mathcal {U}}\big )+\sigma _{2}\bar{\mathcal {M}}_{\textbf{b}}(\zeta )d\mathcal {W}_{2}(\zeta ),~if~{\mathbb {T}}_{2}\le \zeta \le {\mathbb {T}},\\ d{\bar{\mathcal {M}}_{\textbf{g}}}(\zeta )=\big (\lambda _{\textbf{g}}\mathcal {S}_{\textbf{f}}\bar{\mathcal {M}}_{\textbf{g}}-(\gamma _{\textbf{g}}+\delta _{\textbf{g}}+\vartheta )\bar{\mathcal {M}}_{\textbf{g}}+\chi _{\textbf{g}}\bar{\mathcal {U}}\big )+\sigma _{3}\bar{\mathcal {M}}_{\textbf{g}}(\zeta )d\mathcal {W}_{3}(\zeta ),\\ d{\bar{\mathcal {U}}}(\zeta )=\big (\gamma _{\textbf{b}}\bar{\mathcal {M}}_{\textbf{b}}+\gamma _{\textbf{g}}\bar{\mathcal {M}}_{\textbf{g}}-(\chi _{\textbf{b}}+\chi _{\textbf{g}}+\vartheta )\bar{\mathcal {U}}\big )+\sigma _{4}\bar{\mathcal {U}}(\zeta )d\mathcal {W}_{r}(\zeta ). \end{array}\right. } \end{aligned}$$

Here, we employ the method reported in^36^ for the situation of Caputo-Fabrizio derivative to calculate and investigate the piecewise configuration (4.7)–(4.9). We begin the methodology by doing the following:

4.10 $$\begin{aligned} {\left\{ \begin{array}{ll} \frac{d\tilde{\mho }_{\ell }(\zeta )}{d\zeta }=\Phi (\zeta ,\tilde{\mho }_{\ell }).~\tilde{\mho }_{\ell }(0)=\tilde{\mho }_{\ell ,0},~\ell =1,2,...,\mathfrak {n}~if~\zeta \in [0,{\mathbb {T}}_{1}],\\ \,_{{\mathbb {T}}_{1}}^{CF}\textbf{D}_{\zeta }^{\Lambda }\tilde{\mho }_{\ell }(\zeta )=\Phi (\zeta ,\tilde{\mho }_{\ell }),~\tilde{\mho }_{\ell }({\mathbb {T}}_{1})=\tilde{\mho }_{\ell ,1},~if~\zeta \in [{\mathbb {T}}_{1},{\mathbb {T}}_{2}],\\ d\tilde{\mho }_{\ell }(\zeta )=\Phi (\zeta ,\tilde{\mho }_{\ell })d\zeta +\wp _{\ell }\tilde{\mho }_{\ell }d\mathcal {W}_{\ell }(\zeta ),~\tilde{\mho }_{\ell }({\mathbb {T}}_{2})=\tilde{\mho }_{\ell ,2},~if~\zeta \in [{\mathbb {T}}_{2},{\mathbb {T}}]. \end{array}\right. } \end{aligned}$$

It is worth noting that

4.11 $$\begin{aligned} \tilde{\mho }_{\ell }^{\textbf{w}}={\left\{ \begin{array}{ll}\tilde{\mho }_{\ell }(0)+\sum \limits _{\varsigma =2}^{\textbf{w}}\Big \{\frac{23}{12}\Phi ({\zeta }_{\varsigma },\tilde{\mho }^{\varsigma })\Delta \zeta -\frac{4}{3}\Phi ({\zeta }_{\varsigma -1},\tilde{\mho }^{\varsigma -1})\Delta \zeta +\frac{5}{12}\Phi ({\zeta }_{\varsigma -2},\tilde{\mho }^{\varsigma -2})\Delta \zeta \Big \},~~\zeta \in [0,\mathbb {T_{1}}].\\ \tilde{\mho }_{\ell }({\mathbb {T}}_{1})+\frac{1-\Lambda }{{\mathbb {M}}(\Lambda )}\Phi ({\zeta }_{\mathfrak {n}},\tilde{\mho }^{\mathfrak {n}})+\frac{\Lambda }{{\mathbb {M}}(\Lambda )}\sum \limits _{\varsigma =2}^{\textbf{w}}\Big \{\frac{5}{12}\Phi ({\zeta }_{\varsigma -2},\tilde{\mho }^{\varsigma -2})\Delta \zeta -\frac{4}{3}\Phi ({\zeta }_{\varsigma -1},\tilde{\mho }^{\varsigma -1})\Delta \zeta \\ \quad +\frac{23}{12}\Phi ({\zeta }_{\varsigma },\tilde{\mho }^{\varsigma })\Delta \zeta \Big \},~~\zeta \in [{\mathbb {T}}_{1},{\mathbb {T}}_{2}],\\ \tilde{\mho }_{\ell }({\mathbb {T}}_{2})+\sum \limits _{\varsigma =\textbf{w}+3}^{\mathfrak {n}}\Big \{\frac{5}{12}\Phi ({\zeta }_{\varsigma -2},\tilde{\mho }^{\varsigma -2})\Delta \zeta -\frac{4}{3}\Phi ({\zeta }_{\varsigma -1},\tilde{\mho }^{\varsigma -1})\Delta \zeta +\frac{23}{12}\Phi ({\zeta }_{\varsigma },\tilde{\mho }^{\varsigma })\Delta \zeta \Big \} \\ \quad +\sum \limits _{\varsigma =\textbf{w}+3}^{\mathfrak {n}}\Big \{\frac{5}{12}\big (\mathcal {W}({\zeta }_{\varsigma -1})-\mathcal {W}({\zeta }_{\varsigma -2})\big )\wp \tilde{\mho }^{\varsigma -2}- \frac{4}{3}\big (\mathcal {W}({\zeta }_{\varsigma })-\mathcal {W}({\zeta }_{\varsigma -1})\big )\wp \tilde{\mho }^{\varsigma -1}\\ \quad +\frac{23}{12}\big (\mathcal {W}({\zeta }_{\varsigma +1})-\mathcal {W}({\zeta }_{\varsigma })\big )\wp \tilde{\mho }^{\varsigma }\Big \},~~\zeta \in [{\mathbb {T}}_{2},{\mathbb {T}}].\end{array}\right. } \end{aligned}$$

### Generalized Mittag-Leffler kernel

In this section, we will focus on the nonlinear behaviour of malnutrition that also illustrates multiple phases for the transport of malnutrition and underweight individuals in the community, such as integer-order, generalized Mittag-Leffler law, and dynamical provokes. If ${\mathbb {T}}$ is defined as the final time, the computational framework will be established during the initial stage using the classical derivative implementation, followed by the Mittag-Leffler kernel in the other approach, and consequently the Gaussian noise in future periods. The scientific formula used to explain the manifestation in this context is as follows:

4.12 $$\begin{aligned} {\left\{ \begin{array}{ll} \frac{d\mathcal {S}_{\textbf{f}}}{d\zeta }=(\mathcal {B}+\varepsilon )-(\lambda _{\textbf{b}}\bar{\mathcal {M}}_{\textbf{b}}+\lambda _{\textbf{g}}\bar{\mathcal {M}}_{\textbf{g}}+\vartheta )\mathcal {S}_{\textbf{f}}+\delta _{\textbf{g}}\bar{\mathcal {M}}_{\textbf{g}},\\ \frac{d\bar{\mathcal {M}}_{\textbf{b}}}{d\zeta }=\lambda _{\textbf{b}}\mathcal {S}_{\textbf{f}}\bar{\mathcal {M}}_{\textbf{b}}-(\vartheta _{\textbf{b}}+\gamma _{\textbf{b}}+\vartheta )\bar{\mathcal {M}}_{\textbf{b}}+\chi _{\textbf{b}}\bar{\mathcal {U}},~if~0\le \zeta \le {\mathbb {T}}_{1},\\ \frac{d\bar{\mathcal {M}}_{\textbf{g}}}{d\zeta }=\lambda _{\textbf{g}}\mathcal {S}_{\textbf{f}}\bar{\mathcal {M}}_{\textbf{g}}-(\gamma _{\textbf{g}}+\delta _{\textbf{g}}+\vartheta )\bar{\mathcal {M}}_{\textbf{g}}+\chi _{\textbf{g}}\bar{\mathcal {U}},\\ \frac{d\bar{\mathcal {U}}}{d\zeta }=\gamma _{\textbf{b}}\bar{\mathcal {M}}_{\textbf{b}}+\gamma _{\textbf{g}}\bar{\mathcal {M}}_{\textbf{g}}-(\chi _{\textbf{b}}+\chi _{\textbf{g}}+\vartheta )\bar{\mathcal {U}},\end{array}\right. } \end{aligned}$$

4.13 $$\begin{aligned} {\left\{ \begin{array}{ll} \,_{0}^{ABC}\textbf{D}_{\zeta }^{\Lambda }{\mathcal {S}_{\textbf{f}}}=(\mathcal {B}+\varepsilon )-(\lambda _{\textbf{b}}\bar{\mathcal {M}}_{\textbf{b}}+\lambda _{\textbf{g}}\bar{\mathcal {M}}_{\textbf{g}}+\vartheta )\mathcal {S}_{\textbf{f}}+\delta _{\textbf{g}}\bar{\mathcal {M}}_{\textbf{g}},\\ \,_{0}^{ABC}\textbf{D}_{\zeta }^{\Lambda }{\bar{\mathcal {M}}_{\textbf{b}}}=\lambda _{\textbf{b}}\mathcal {S}_{\textbf{f}}\bar{\mathcal {M}}_{\textbf{b}}-(\vartheta _{\textbf{b}}+\gamma _{\textbf{b}}+\vartheta )\bar{\mathcal {M}}_{\textbf{b}}+\chi _{\textbf{b}}\bar{\mathcal {U}},~if~{\mathbb {T}}_{1}\le \zeta \le {\mathbb {T}}_{2},\\ \,_{0}^{ABC}\textbf{D}_{\zeta }^{\Lambda }{\bar{\mathcal {M}}_{\textbf{g}}}=\lambda _{\textbf{g}}\mathcal {S}_{\textbf{f}}\bar{\mathcal {M}}_{\textbf{g}}-(\gamma _{\textbf{g}}+\delta _{\textbf{g}}+\vartheta )\bar{\mathcal {M}}_{\textbf{g}}+\chi _{\textbf{g}}\bar{\mathcal {U}},\\ \,_{0}^{ABC}\textbf{D}_{\zeta }^{\Lambda }{\bar{\mathcal {U}}}=\gamma _{\textbf{b}}\bar{\mathcal {M}}_{\textbf{b}}+\gamma _{\textbf{g}}\bar{\mathcal {M}}_{\textbf{g}}-(\chi _{\textbf{b}}+\chi _{\textbf{g}}+\vartheta )\bar{\mathcal {U}},\end{array}\right. } \end{aligned}$$

4.14 $$\begin{aligned} {\left\{ \begin{array}{ll} d{\mathcal {S}_{\textbf{f}}}(\zeta )=\big ((\mathcal {B}+\varepsilon )-(\lambda _{\textbf{b}}\bar{\mathcal {M}}_{\textbf{b}}+\lambda _{\textbf{g}}\bar{\mathcal {M}}_{\textbf{g}}+\vartheta )\mathcal {S}_{\textbf{f}}+\delta _{\textbf{g}}\bar{\mathcal {M}}_{\textbf{g}}\big )+\sigma _{1}\mathcal {S}_{\textbf{f}}(\zeta )d\mathcal {W}_{1}(\zeta ),\\ d{\bar{\mathcal {M}}_{\textbf{b}}}(\zeta )=\big (\lambda _{\textbf{b}}\mathcal {S}_{\textbf{f}}\bar{\mathcal {M}}_{\textbf{b}}-(\vartheta _{\textbf{b}}+\gamma _{\textbf{b}}+\vartheta )\bar{\mathcal {M}}_{\textbf{b}}+\chi _{\textbf{b}}\bar{\mathcal {U}}\big )+\sigma _{2}\bar{\mathcal {M}}_{\textbf{b}}(\zeta )d\mathcal {W}_{2}(\zeta ),~if~{\mathbb {T}}_{2}\le \zeta \le {\mathbb {T}},\\ d{\bar{\mathcal {M}}_{\textbf{g}}}(\zeta )=\big (\lambda _{\textbf{g}}\mathcal {S}_{\textbf{f}}\bar{\mathcal {M}}_{\textbf{g}}-(\gamma _{\textbf{g}}+\delta _{\textbf{g}}+\vartheta )\bar{\mathcal {M}}_{\textbf{g}}+\chi _{\textbf{g}}\bar{\mathcal {U}}\big )+\sigma _{3}\bar{\mathcal {M}}_{\textbf{g}}(\zeta )d\mathcal {W}_{3}(\zeta ),\\ d{\bar{\mathcal {U}}}(\zeta )=\big (\gamma _{\textbf{b}}\bar{\mathcal {M}}_{\textbf{b}}+\gamma _{\textbf{g}}\bar{\mathcal {M}}_{\textbf{g}}-(\chi _{\textbf{b}}+\chi _{\textbf{g}}+\vartheta )\bar{\mathcal {U}}\big )+\sigma _{4}\bar{\mathcal {U}}(\zeta )d\mathcal {W}_{r}(\zeta ). \end{array}\right. } \end{aligned}$$

Here, we employ the method reported in^36^ for the case of Atangana-Baleanu derivative to calculate and investigate the piecewise configuration (4.12)–(4.14). We begin the methodology by doing the following:

$$\begin{aligned} {\left\{ \begin{array}{ll} \frac{d\tilde{\mho }_{\ell }(\zeta )}{d\zeta }=\Phi (\zeta ,\tilde{\mho }_{\ell }).~\tilde{\mho }_{\ell }(0)=\tilde{\mho }_{\ell ,0},~\ell =1,2,...,\mathfrak {n}~if~\zeta \in [0,{\mathbb {T}}_{1}],\\ \,_{{\mathbb {T}}_{1}}^{ABC}\textbf{D}_{\zeta }^{\Lambda }\tilde{\mho }_{\ell }(\zeta )=\Phi (\zeta ,\tilde{\mho }_{\ell }),~\tilde{\mho }_{\ell }({\mathbb {T}}_{1})=\tilde{\mho }_{\ell ,1},~if~\zeta \in [{\mathbb {T}}_{1},{\mathbb {T}}_{2}],\\ d\tilde{\mho }_{\ell }(\zeta )=\Phi (\zeta ,\tilde{\mho }_{\ell })d\zeta +\wp _{\ell }\tilde{\mho }_{\ell }d\mathcal {W}_{\ell }(\zeta ),~\tilde{\mho }_{\ell }({\mathbb {T}}_{2})=\tilde{\mho }_{\ell ,2},~if~\zeta \in [{\mathbb {T}}_{2},{\mathbb {T}}]. \end{array}\right. } \end{aligned}$$

It is worth noting that

$$\begin{aligned} \tilde{\mho }_{\ell }^{\textbf{w}}={\left\{ \begin{array}{ll}\tilde{\mho }_{\ell }(0)+\sum \limits _{\varsigma =2}^{\textbf{w}}\Big \{\frac{23}{12}\Phi ({\zeta }_{\varsigma },\tilde{\mho }^{\varsigma })\Delta \zeta -\frac{4}{3}\Phi ({\zeta }_{\varsigma -1},\tilde{\mho }^{\varsigma -1})\Delta \zeta +\frac{5}{12}\Phi ({\zeta }_{\varsigma -2},\tilde{\mho }^{\varsigma -2})\Delta \zeta \Big \},~~\zeta \in [0,\mathbb {T_{1}}].\\ \tilde{\mho }_{\ell }({\mathbb {T}}_{1})+\frac{\Lambda }{ABC(\Lambda )}\Phi ({\zeta }_{\mathfrak {n}},\tilde{\mho }^{\mathfrak {n}})+\frac{\Lambda (\Delta \zeta )^{\Lambda -1}}{ABC(\Lambda )\Gamma (\Lambda +1)}\sum \limits _{\varsigma =2}^{\textbf{w}}\Phi ({\zeta }_{\varsigma -2},\tilde{\mho }^{\varsigma -2})\Im _{1}\\ \quad + \frac{\Lambda (\Delta \zeta )^{\Lambda -1}}{ABC(\Lambda )\Gamma (\Lambda +2)}\sum \limits _{\varsigma =2}^{\textbf{w}}\Big \{\Phi ({\zeta }_{\varsigma -1},\tilde{\mho }^{\varsigma -1})-\Phi ({\zeta }_{\varsigma -2},\tilde{\mho }^{\varsigma -2})\Big \}\Im _{2}\\ \quad +\frac{\Lambda (\Delta \zeta )^{\Lambda -1}}{2ABC(\Lambda )\Gamma (\Lambda +3)}\sum \limits _{\varsigma =2}^{\textbf{w}}\Big \{\Phi ({\zeta }_{\varsigma },\tilde{\mho }^{\varsigma })-2\Phi ({\zeta }_{\varsigma -1},\tilde{\mho }^{\varsigma -1})+\Phi ({\zeta }_{\varsigma -2},\tilde{\mho }^{\varsigma -2})\Big \}\Im _{3},~~\zeta \in [{\mathbb {T}}_{1},{\mathbb {T}}_{2}],\\ \tilde{\mho }_{\ell }({\mathbb {T}}_{2})+\sum \limits _{\varsigma =\textbf{w}+3}^{\mathfrak {n}}\Big \{\frac{5}{12}\Phi ({\zeta }_{\varsigma -2},\tilde{\mho }^{\varsigma -2})\Delta \zeta -\frac{4}{3}\Phi ({\zeta }_{\varsigma -1},\tilde{\mho }^{\varsigma -1})\Delta \zeta +\frac{23}{12}\Phi ({\zeta }_{\varsigma },\tilde{\mho }^{\varsigma })\Delta \zeta \Big \} \\ \quad +\sum \limits _{\varsigma =\textbf{w}+3}^{\mathfrak {n}}\Big \{\frac{5}{12}\big (\mathcal {W}({\zeta }_{\varsigma -1})-\mathcal {W}({\zeta }_{\varsigma -2})\big )\wp \tilde{\mho }^{\varsigma -2}- \frac{4}{3}\big (\mathcal {W}({\zeta }_{\varsigma })-\mathcal {W}({\zeta }_{\varsigma -1})\big )\wp \tilde{\mho }^{\varsigma -1}\\ \quad +\frac{23}{12}\big (\mathcal {W}({\zeta }_{\varsigma +1})-\mathcal {W}({\zeta }_{\varsigma })\big )\wp \tilde{\mho }^{\varsigma }\Big \},~~\zeta \in [{\mathbb {T}}_{2},{\mathbb {T}}], \end{array}\right. } \end{aligned}$$

where $\Im _{1},\Im _{2}$ and $\Im _{3}$ are defined in (4.4)–(4.6).

## Results and discussion

The simulation results of the framework (2.2) for all four sets of data reveal that malnutrition and body immunization have a massive effect on undernourished pregnant females $\mathcal {S}_{\textbf{f}}$, conceive famished boys $\bar{\mathcal {M}}_{\textbf{b}}$, girls $\bar{\mathcal {M}}_{\textbf{g}}$, and underweight individuals $\bar{\mathcal {U}}$ via the crossover effects. Various dietary prestige and distinct immune defence stages have various sorts of effects, as shown by the graphs of differential equations for malnourished individuals utilizing the numerical scheme proposed by Atangana and Araz^36^. To overcome the malnutrition problem, an initial value and random intensities are required. It determines the variation in attributes over time depending on that initial value. To test the modifications in all three scenarios, we employ one initial value and steadily observe how initial values affect the modification. Also, ${\mathbb {R}}_{0}^{s}=1.245>1,$ where ${\mathbb {R}}_{0}^{s}$ is described in Section 3. We can verify that system (2.2) will persist for a long time using the findings of Theorem 3.3 and a distribution of $\pi (.).$ The numerical simulations below confirm this. Let us now examine the consequences for each individual.

**Figure 2:**
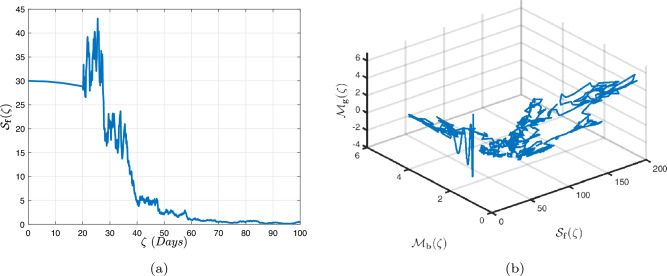
Two-dimensional view and phase portrait of dynamic pattern of malnutrition system (4.1)–(4.3) for undernourished pregnant women $\mathcal {S}_{\textbf{f}}$ using Caputo fractional derivative of order $\Lambda =0.95$ with lowest random perturbations.

**Figure 3:**
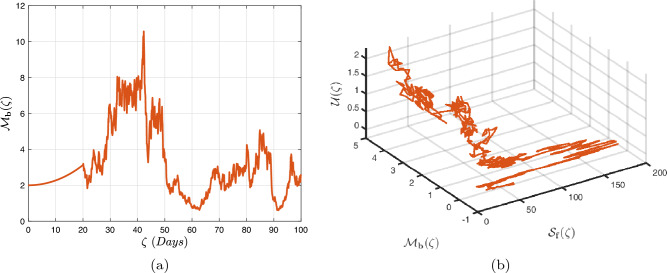
Two-dimensional view and phase portrait of dynamic pattern of malnutrition system (4.1)–(4.3) for birth to malnourished boys $\bar{\mathcal {M}}_{\textbf{b}}$ using Caputo fractional derivative of order $\Lambda =0.95$ with lowest random perturbations.

**Figure 4:**
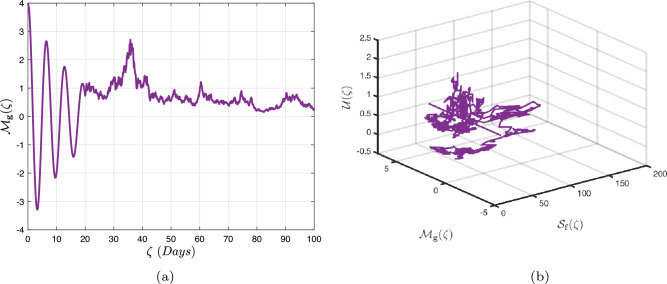
Two-dimensional view and phase portrait of dynamic pattern of malnutrition system (4.1)–(4.3) for birth to malnourished girls $\bar{\mathcal {M}}_{\textbf{g}}$ using Caputo fractional derivative of order $\Lambda =0.95$ with lowest random perturbations.

**Figure 5:**
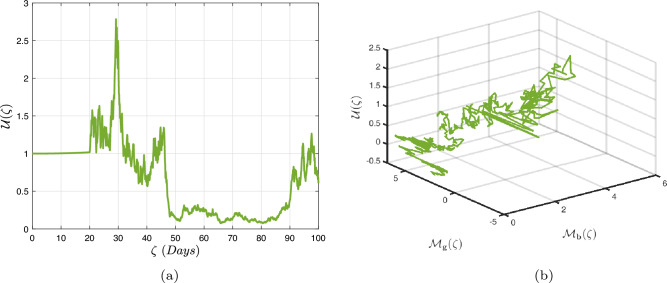
Two-dimensional view and phase portrait of dynamic pattern of malnutrition system (4.1)–(4.3) for underweight $\bar{\mathcal {U}}$ using Caputo fractional derivative of order $\Lambda =0.95$ with lowest random perturbations.

Figures 2a and b depict the modifications in undernourished pregnant females’ cases for normal nutrient intake, Figure 3a and b represents the view of birth to malnourished boys, Figure 4a and b denotes birth of malnourished girls and Figure 5a and b represents the under weight individuals with immune function in the sets of parameters under various random intensities $\sigma _{1}=0.08,~\sigma _{2}=0.09,~\sigma _{3}=0.1,~\sigma _{4}=0.12$ and initial conditions $\mathcal {S}_{\textbf{f}}(0)=30,~\bar{\mathcal {M}}_{\textbf{b}}=2,~\bar{\mathcal {M}}_{\textbf{g}}=4$ and $\bar{\mathcal {U}}=1,$ respectively via the piecewise fractional differential equations techniques. For the first set of values, we explore that a starting value of 30 results in linear decay, whereas values 2,  4,  and 1 result in logarithmic growth. It shows logarithmic and wave growth for all random intensities in the second, third, and fourth sets of values when the Caputo fractional derivative is convoluted with the deterministic-stochastic case. The significance of immune function and nourishment is evident from the research, and it is interesting to note that maintaining strong immunity and appropriate nourishment in the bloodstream will substantially decrease hypersensitivity, decrease the risk of infestation, and improve the mental health process.

**Figure 6:**
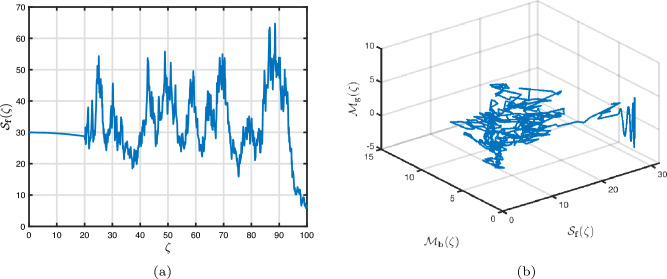
Two-dimensional view and phase portrait of dynamic pattern of malnutrition system (4.7)–(4.9) for undernourished pregnant women $\mathcal {S}_{\textbf{f}}$ using Caputo-Fabrizio fractional derivative of order $\Lambda =0.95$ with lowest random perturbations.

**Figure 7:**
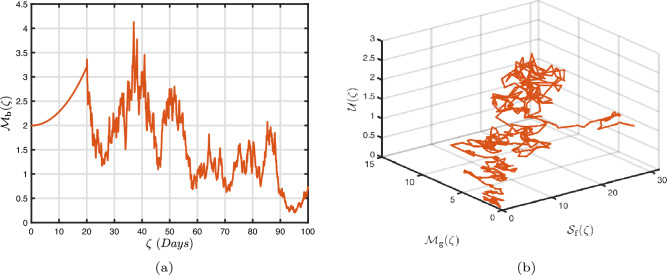
Two-dimensional view and phase portrait of dynamic pattern of malnutrition system (4.7)–(4.9) for birth to malnourished boys $\bar{\mathcal {M}}_{\textbf{b}}$ using Caputo-Fabrizio fractional derivative of order $\Lambda =0.95$ with lowest random perturbations.

**Figure 8:**
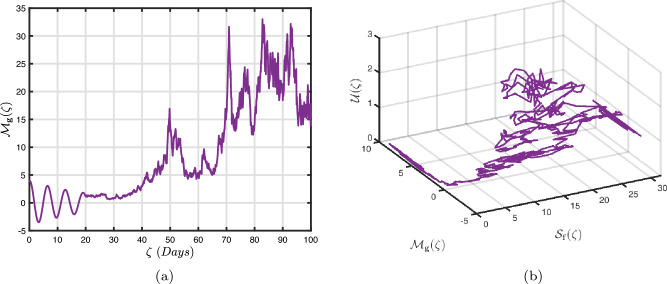
Two-dimensional view and phase portrait of dynamic pattern of malnutrition system (4.7)–(4.9) for birth to malnourished girls $\bar{\mathcal {M}}_{\textbf{g}}$ using Caputo-Fabrizio fractional derivative of order $\Lambda =0.95$ with lowest random perturbations.

**Figure 9:**
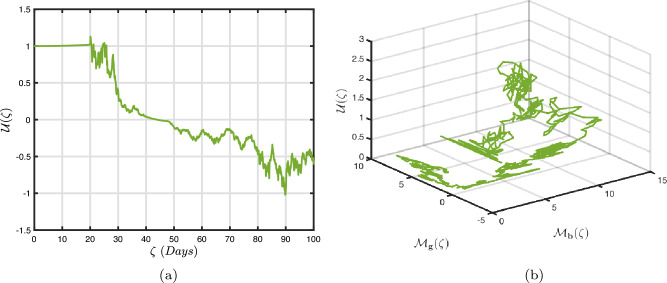
Two-dimensional view and phase portrait of dynamic pattern of malnutrition system (4.7)–(4.9) for underweight individuals $\bar{\mathcal {U}}$ using Caputo-Fabrizio fractional derivative of order $\Lambda =0.95$ with lowest random perturbations.

Figure 6a and b depict the modifications in undernourished pregnant females’ cases for normal nutrient intake, Fig. 7a and b represents the view of birth to malnourished boys, Fig. 8a and b denotes birth of malnourished girls and Fig, 9a and b represents the under weight individuals of variables with varying random intensities, $\sigma _{1}=0.08,~\sigma _{2}=0.09,~\sigma _{3}=0.1,~\sigma _{4}=0.12$ and initial conditions $\mathcal {S}_{\textbf{f}}(0)=30,~\bar{\mathcal {M}}_{\textbf{b}}=2,~\bar{\mathcal {M}}_{\textbf{g}}=4$ and $\bar{\mathcal {U}}=1,$ respectively via the piecewise fractional differential equations approaches. For the first set of values, we notice that a starting value of 30 results in linear decay, whereas values 2,  4,  and 1 result in logarithmic growth. It shows logarithmic and wave growth for all random intensities in the second, third, and fourth sets of values when the Caputo-Fabrizo fractional derivative is merged with the deterministic-stochastic scenario. The significance of immune function and nourishment is evident from the research, and it is interesting to note that maintaining strong immunity and appropriate nourishment in the bloodstream will substantially decrease hypersensitivity, decrease the risk of infestation, and improve the mental health process. It is indeed clear from simulation analysis that the consequences of dietary patterns and immune function tend to vary with changes in other attributes connected to the model’s conceptualization. This system will assist those responsible for attempting to make decisions to ameliorate losses incurred by complexities in pregnancy.

**Figure 10:**
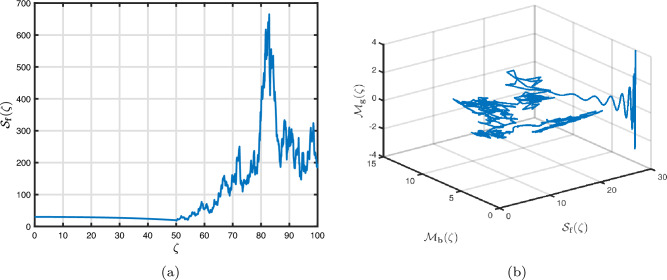
Two-dimensional view and phase portrait of dynamic pattern of malnutrition system (4.12)–(4.14) for undernourished pregnant women $\mathcal {S}_{\textbf{b}}$ using Atangana-Baleanu- Caputo fractional derivative of order $\Lambda =0.95$ with lowest random perturbations.

**Figure 11:**
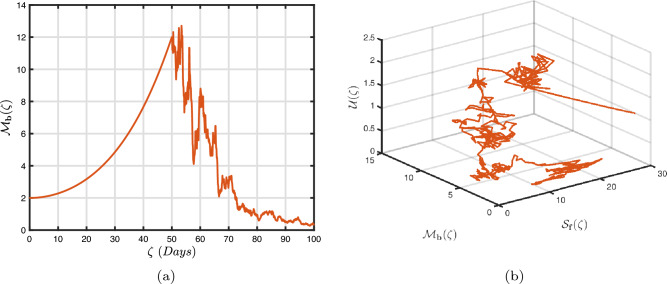
Two-dimensional view and phase portrait of dynamic pattern of malnutrition system (4.12)–(4.14) for birth to malnourished boys $\bar{\mathcal {M}}_{\textbf{b}}$ using Atangana-Baleanu fractional derivative of order $\Lambda =0.95$ with lowest random perturbations.

**Figure 12:**
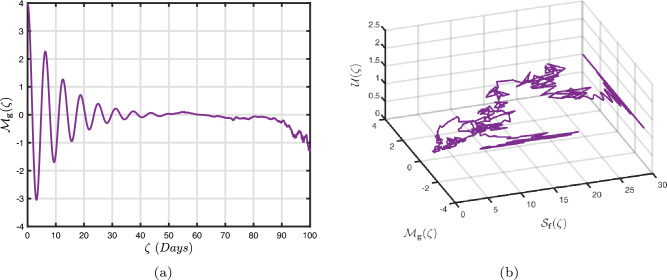
Two-dimensional view and phase portrait of dynamic pattern of malnutrition system (4.12)–(4.14) for birth to malnourished girls $\bar{\mathcal {M}}_{\textbf{g}}$ using Atangana-Baleanu- Caputo fractional derivative of order $\Lambda =0.95$ with lowest random perturbations.

**Figure 13:**
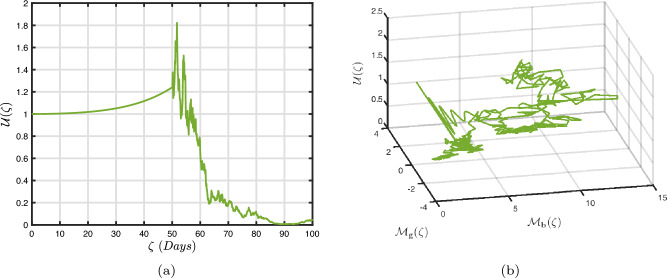
Two-dimensional view and phase portrait of dynamic pattern of malnutrition system (4.12)–(4.14) for underweight individuals $\bar{\mathcal {U}}$ using Atangana-Baleanu- Caputo fractional derivative of order $\Lambda =0.95$ with lowest random perturbations.

Figure 10a and b depict the modifications in undernourished pregnant females’ cases for normal nutrient intake, Fig. 11a and b represents the view of birth to malnourished boys, Fig. 12a and b denotes birth of malnourished girls and Fig. 13a and b represents the under weight individuals utilization and rational immune function were ascertained in a variety of variables with various different random intensities, $\sigma _{1}=0.08,~\sigma _{2}=0.09,~\sigma _{3}=0.1,~\sigma _{4}=0.12$ and initial conditions $\mathcal {S}_{\textbf{f}}(0)=30,~\bar{\mathcal {M}}_{\textbf{b}}=2,~\bar{\mathcal {M}}_{\textbf{g}}=4$ and $\bar{\mathcal {U}}=1,$ respectively via the piecewise fractional differential equations approaches. For the first set of values, we notice that a starting value of 30 results in linear decay, whereas values 2,  4,  and 1 result in logarithmic growth. When the Atangana-Baleanu fractional derivative is combined with the deterministic-stochastic case, it exhibits logarithmic and wave expansion for all random intensities in the second, third, and fourth value systems. Individual’s undernutrition has been assessed to quantify their resistance to destabilization during pregnancy. The immune system is impacted by an effective diet and nutritional requirements. As a result, the only long-term strategy for surviving in the current environment is to boost the immune system, develop diet and exercise plans. This article examines the relevance of nourishment in boosting resistance and provides some skilful and truthful nutritional recommendations for coping with the intricacies of pregnancy.

**Figure 14:**
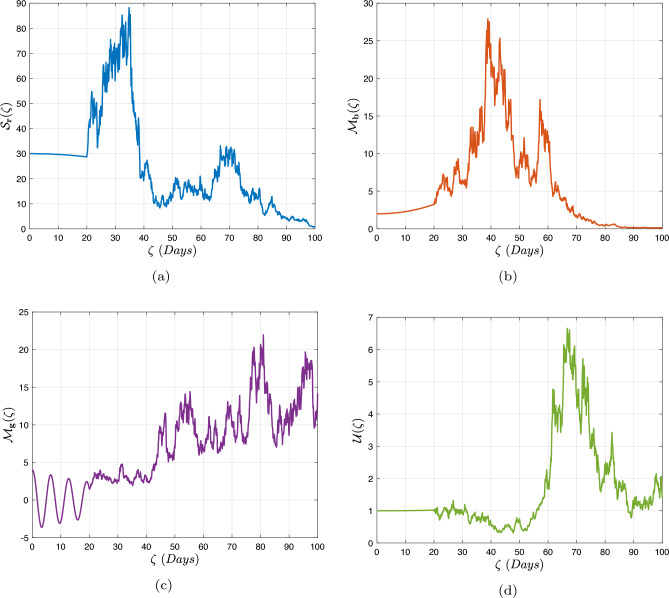
Two-dimensional view of malnutrition system (4.1)–(4.3) using Caputo fractional derivative of order $\Lambda =1$ with lowest random perturbations.

**Figure 15:**
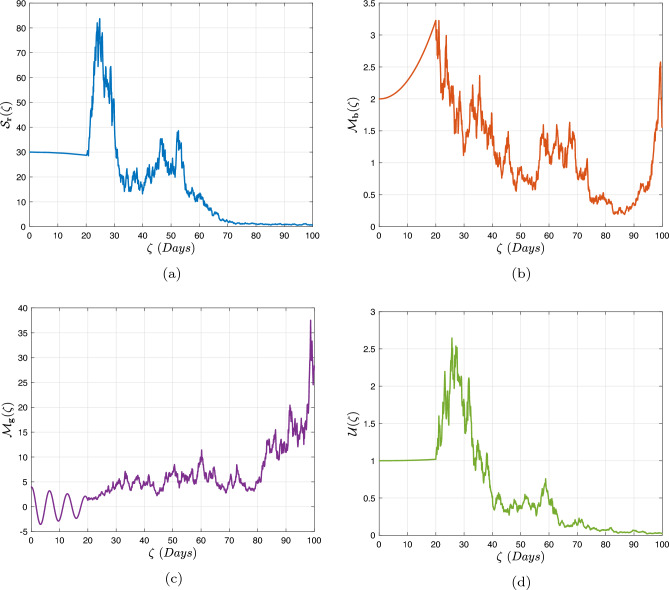
Two-dimensional view of malnutrition system (4.7)–(4.9) using Caputo-Fabrizio fractional derivative of order $\Lambda =1$ with lowest random perturbations.

**Figure 16:**
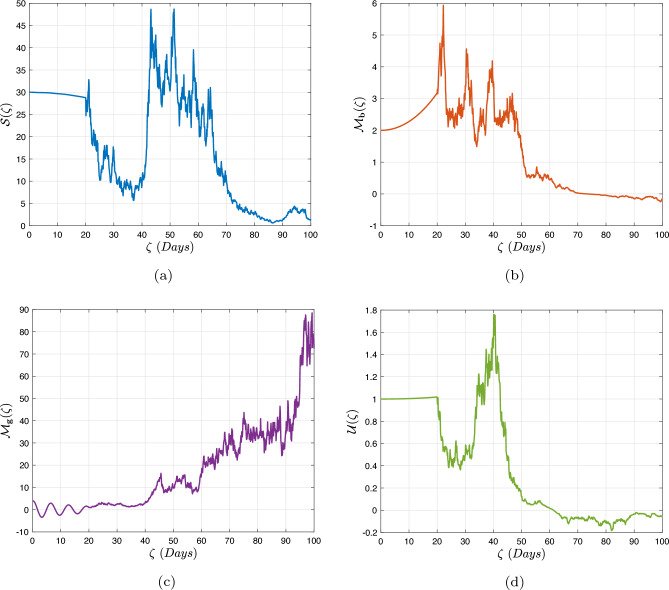
Two-dimensional view of malnutrition system (4.12)–(4.14) using Atangana-Baleanu-Caputo fractional derivative of order $\Lambda =1$ with lowest random perturbations.

The quality of the graphs is very high, with a numerical scheme with respect to the fractional-order $\Lambda =1$ in Figs. 14, 15, 16, close to that of the identified nutritional Caputo-derivative fractional model (4.1)–(4.2), Caputo-Fabrizio fractional derivative model (4.7)–(4.8) and Atangana-Baleanu fractional derivative model (4.12)–(4.14), respectively. This fact shows that an integer-order model can approximate, within a given random perturbation, data generated by a fractional-order one with very high precision without the need for excessively high orders of derivation or computational resources.

**Figure 17:**
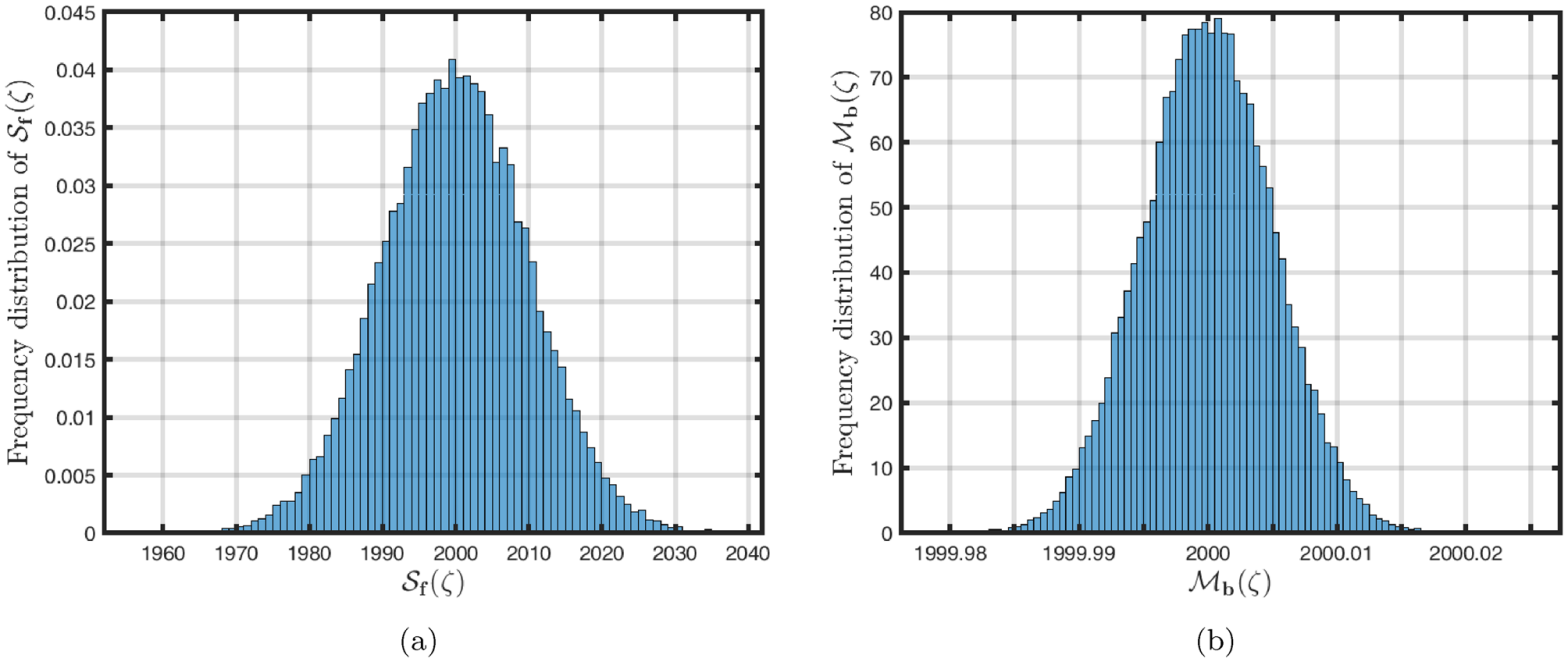
Frequency plots of malnutrition model (2.2) for malnourished pregnant women $\mathcal {S}_{\textbf{f}}$ and birth to malnourished boys $\bar{\mathcal {M}}_{\textbf{b}}$ having probability density function of normal distribution ${\mathbb {N}}(mean,~variance).$.

**Figure 18:**
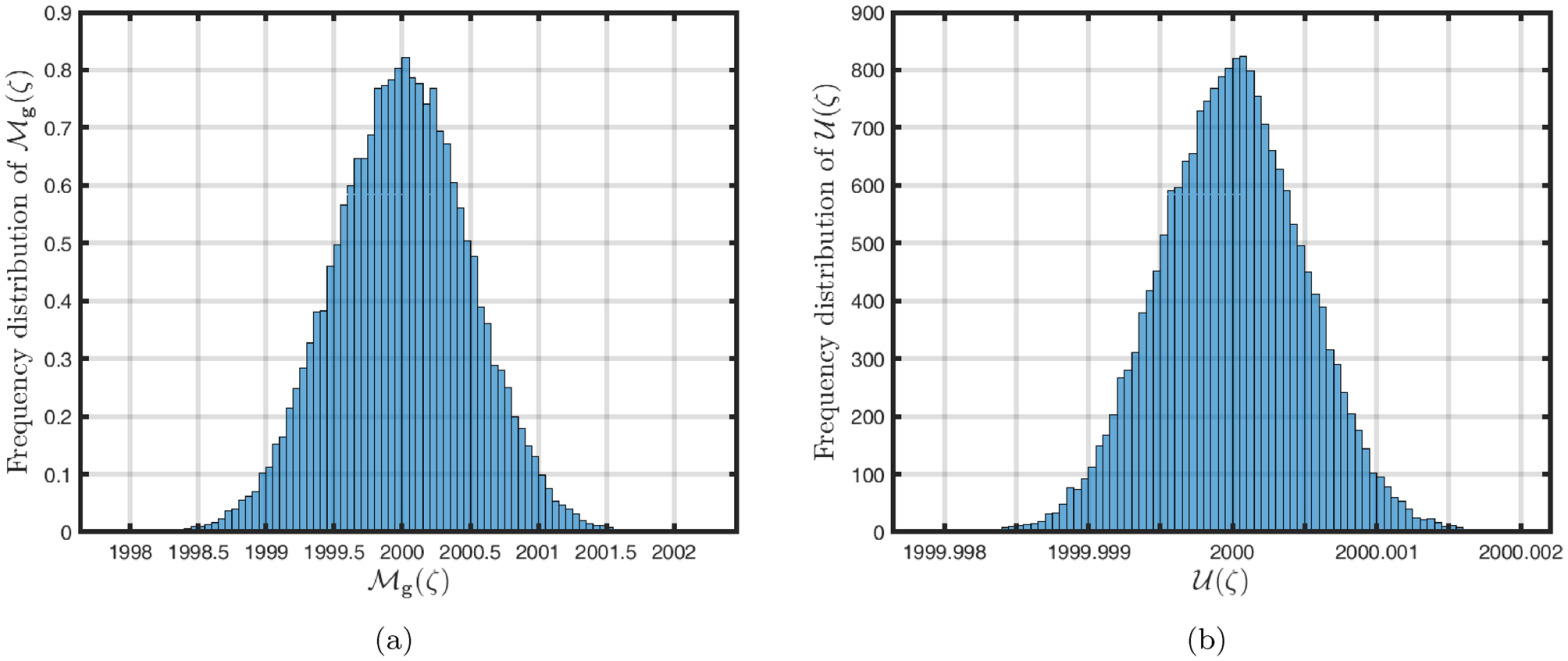
Frequency plots of malnutrition model (2.2) for birth to malnourished girls $\bar{\mathcal {M}}_{\textbf{g}}$ and underweight individuals $\bar{\mathcal {U}}$ having probability density function of normal distribution ${\mathbb {N}}(mean,~variance).$.

Figures 17, 18, represents the histogram plots for the proposed system (2.2). In reality, controlling poor nutrition will not affect the disruption of health issues or the spread of various infections. Simultaneously, when other considerations hinder development of resistance to a newborn child population, such as constant treatment and a healthy life campaign, the number of deaths decreases along with the number of malnourished and underweight. This is essentially consistent with the system (2.2) research findings in this paper.

Finally, these findings indicate that fractional-order techniques are instinctively superior to classical ones when dealing with phenomena such as memory effects and non-local behaviour in general.

## Conclusion

Numerical modelling is useful for analysing societal problems and following up with cost-effective remedies. Fractional calculus and stochastic perturbation, among existing schemes, have a phenomenal capacity for recording, eventually afflicted by noise sources and memory effects, which have been revealed to include almost all biomedical functions. This research represents a deterministic-stochastic framework that employs crossover consequences to predict the intricacies of undernutrition in pregnant women. Initially, we use an inventive interconnection of Lyapunov candidates to determine the existence and uniqueness of the global non-negative outcome corresponding to the unit likelihood of occurrence. The necessary prerequisites for the stationary distribution of poor nutrition are therefore calculated. Whereas the generalized Mittag-Leffler kernel, exponential decay and index law have been shown to be competent at portraying numerous crossover tendencies, we assert that their abilities to achieve this might be strictly limited to the true extent of the environment. In the intervention of undernourishment as well as other insatiable hungers and dietary patterns influencing ailments, the concentration of Gaussian white noise is pivotal. The strategy requires stochastic perturbations (noise) and biological methods to enhance understanding of the scientific studies, which have critical repercussions for antibacterial drugs and genetic engineering. Several other intriguing discussions need to be researched further, such as the fractional nutrition model with Lévy noise and Poisson noise^44, 45^, which can generalize Brownian motion and include several important jump and impulsive random processes often found in neural and financial engineering models.

### Supplementary Information

Supplementary Information.

## Data Availability

The datasets used and/or analyzed during the current study available from the corresponding author on reasonable request.
