# Author Correction: Deterministic-stochastic analysis of fractional differential equations malnutrition model with random perturbations and crossover effects

**DOI:** 10.1038/s41598-023-42901-9

**Published:** 2023-09-19

**Authors:** Yu-Ming Chu, Saima Rashid, Shazia Karim, Aasma Khalid, S. K. Elagan

**Affiliations:** 1 Department of Mathematics, Faculty of Sciences, Huzhou University, Huzhou, China; 2 Department of Mathematics, Government College University, Faisalabad, 38000 Pakistan; 3 Department of Computer Science and Mathematics, Lebanese American University, Beirut, 1401 Lebanon; 4 Department of Basic Sciences and Humanities, UET Lahore, Faisalabad Campus, 54800 Pakistan; 5 Department of Mathematics, Government College for Women University, Faisalabad, Pakistan; 6 Department of Mathematics and Statistics, College of Science, Taif University, P. O. Box 11099, 21944 Taif, Saudi Arabia

Correction to: *Scientific Reports* 10.1038/s41598-023-41861-4, published online 08 September 2023

The original version of this Article contained an error in the Funding section.

“This research has no external funding.”

now reads:

“The researchers would like to acknowledge the Deanship of Scientific Research at Taif University for funding this work.”

The original Article has been corrected.

