# Supplementary figures and images for: Deterministic-stochastic analysis of fractional differential equations malnutrition model with random perturbations and crossover effects
Source: Sci Rep. 2023 Sep 8;13:14824. doi: 10.1038/s41598-023-41861-4 (PMC10491687; doi:10.1038/s41598-023-41861-4)

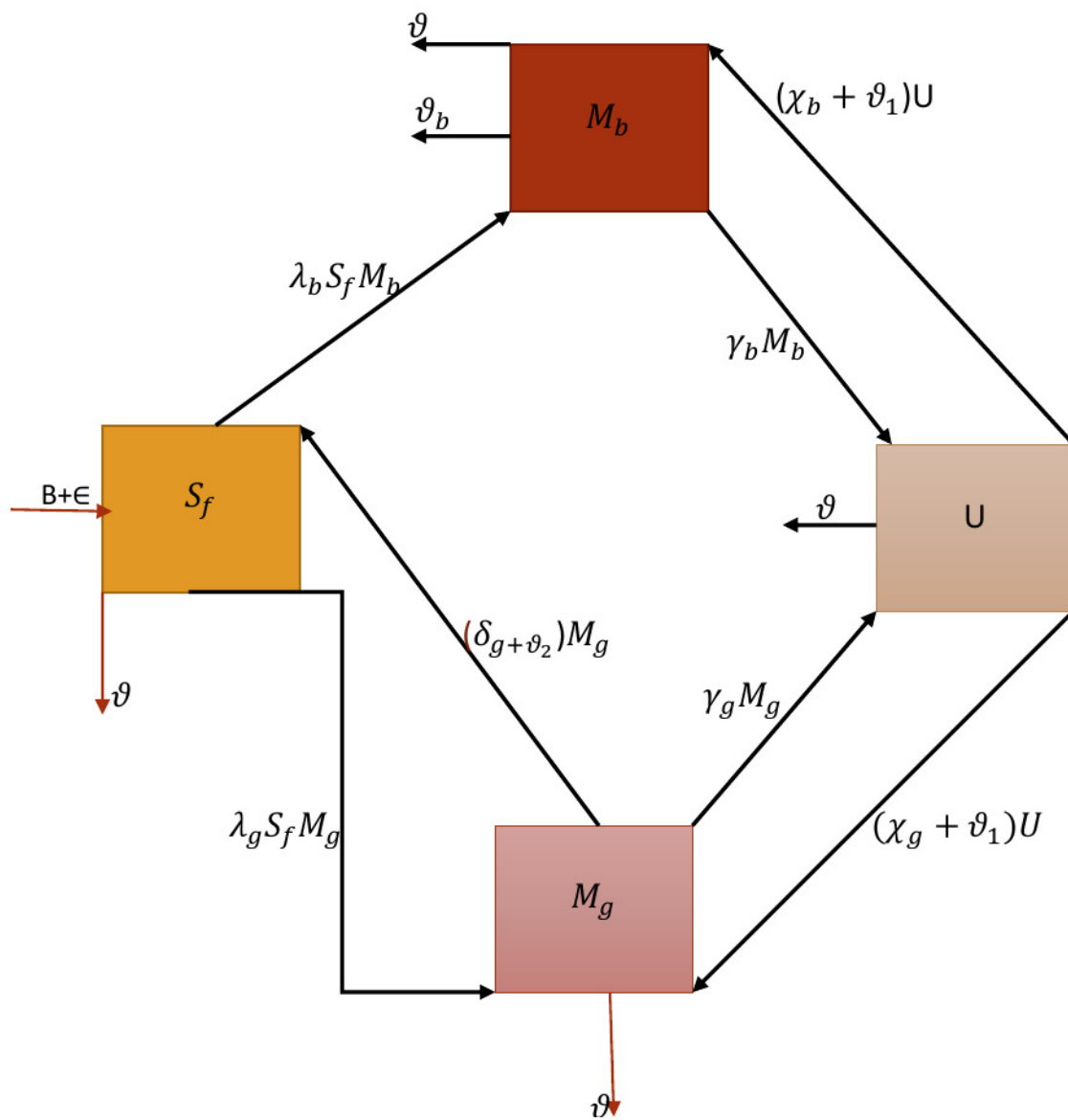

Supplement: Supplementary file 1 — Supplementary Information. [file 41598_2023_41861_MOESM1_ESM.zip › nutrution/Doc10-eps-converted-to.pdf]

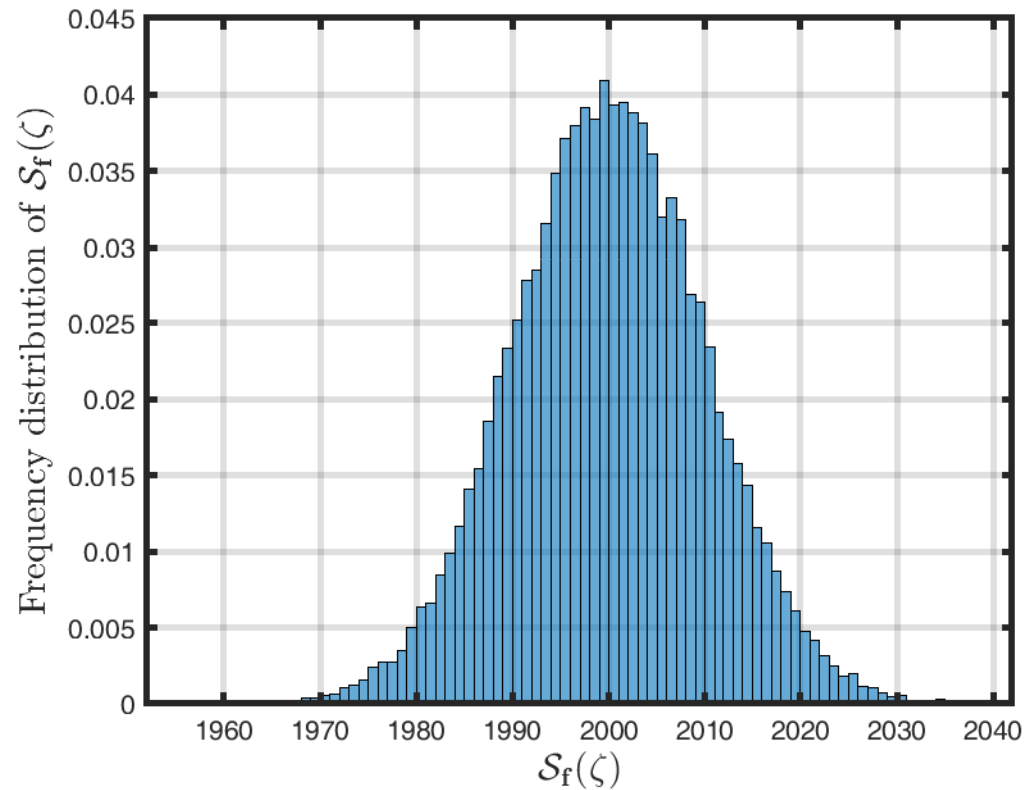

Supplement: Supplementary file 1 — Supplementary Information. [file 41598_2023_41861_MOESM1_ESM.zip › nutrution/Plot1aaaa-eps-converted-to.pdf]

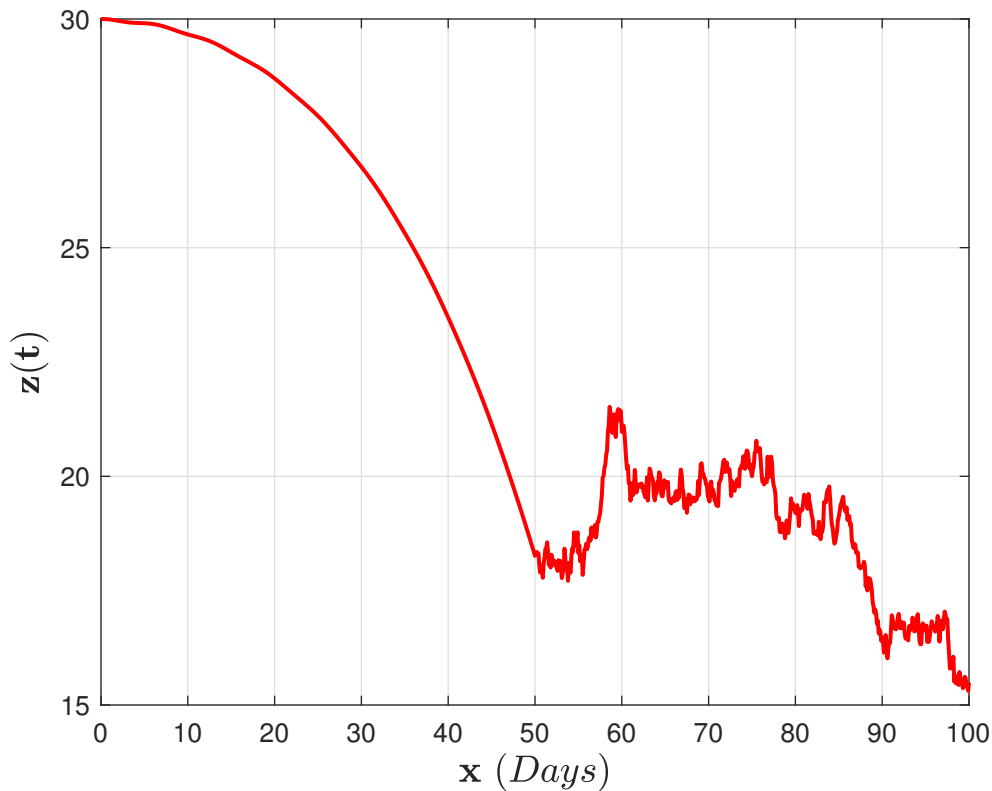

Supplement: Supplementary file 1 — Supplementary Information. [file 41598_2023_41861_MOESM1_ESM.zip › nutrution/Plot1aaa-eps-converted-to.pdf]

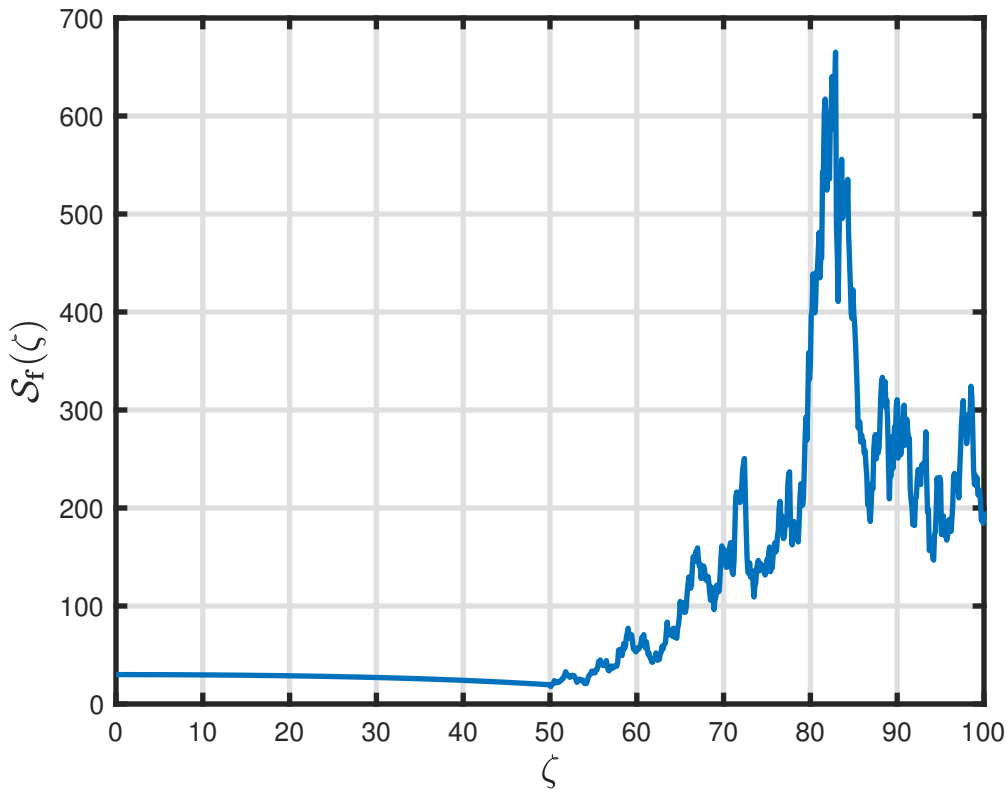

Supplement: Supplementary file 1 — Supplementary Information. [file 41598_2023_41861_MOESM1_ESM.zip › nutrution/Plot1aa-eps-converted-to.pdf]

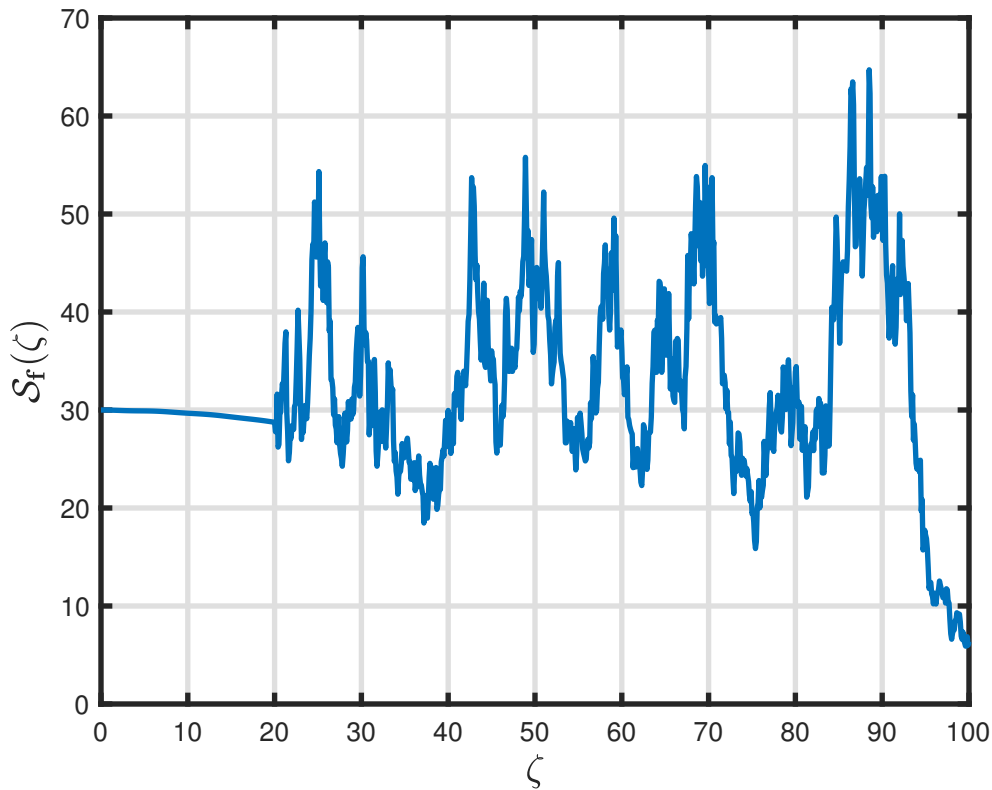

Supplement: Supplementary file 1 — Supplementary Information. [file 41598_2023_41861_MOESM1_ESM.zip › nutrution/Plot1a-eps-converted-to.pdf]

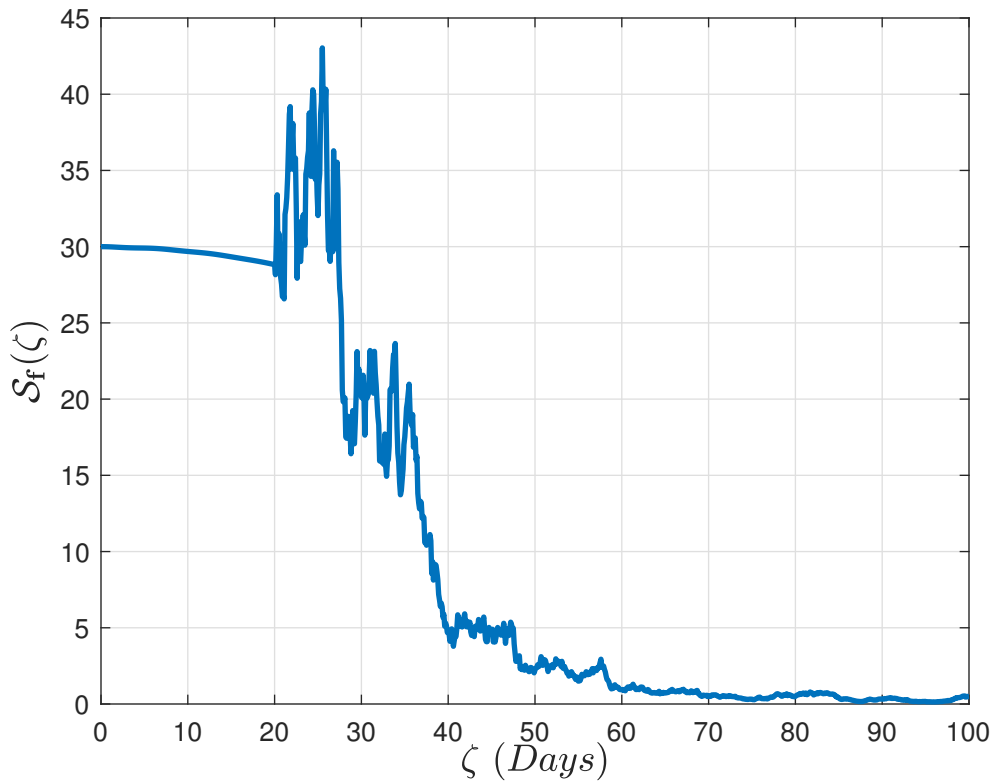

Supplement: Supplementary file 1 — Supplementary Information. [file 41598_2023_41861_MOESM1_ESM.zip › nutrution/Plot1-eps-converted-to.pdf]

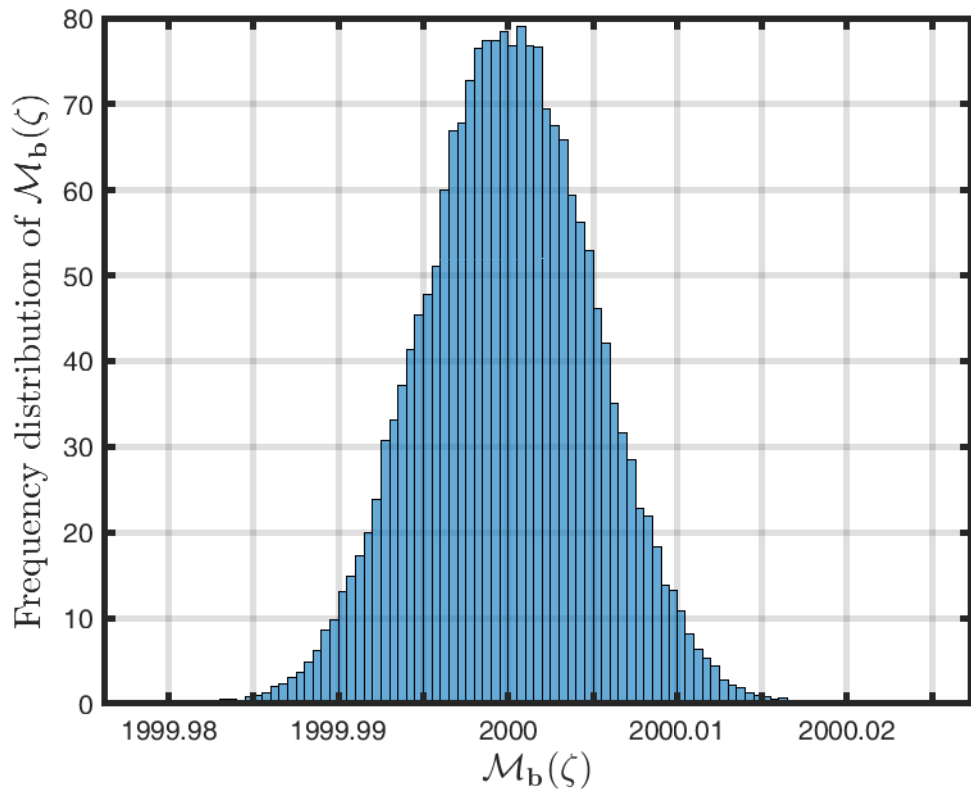

Supplement: Supplementary file 1 — Supplementary Information. [file 41598_2023_41861_MOESM1_ESM.zip › nutrution/Plot2aaaa-eps-converted-to.pdf]

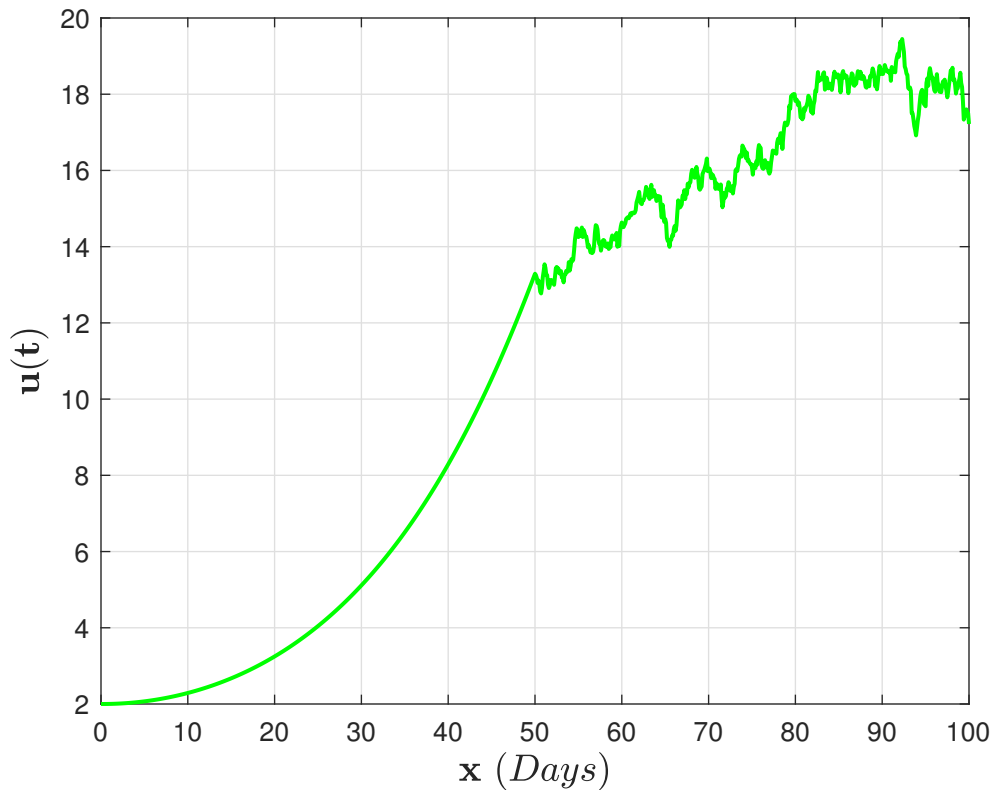

Supplement: Supplementary file 1 — Supplementary Information. [file 41598_2023_41861_MOESM1_ESM.zip › nutrution/Plot2aaa-eps-converted-to.pdf]

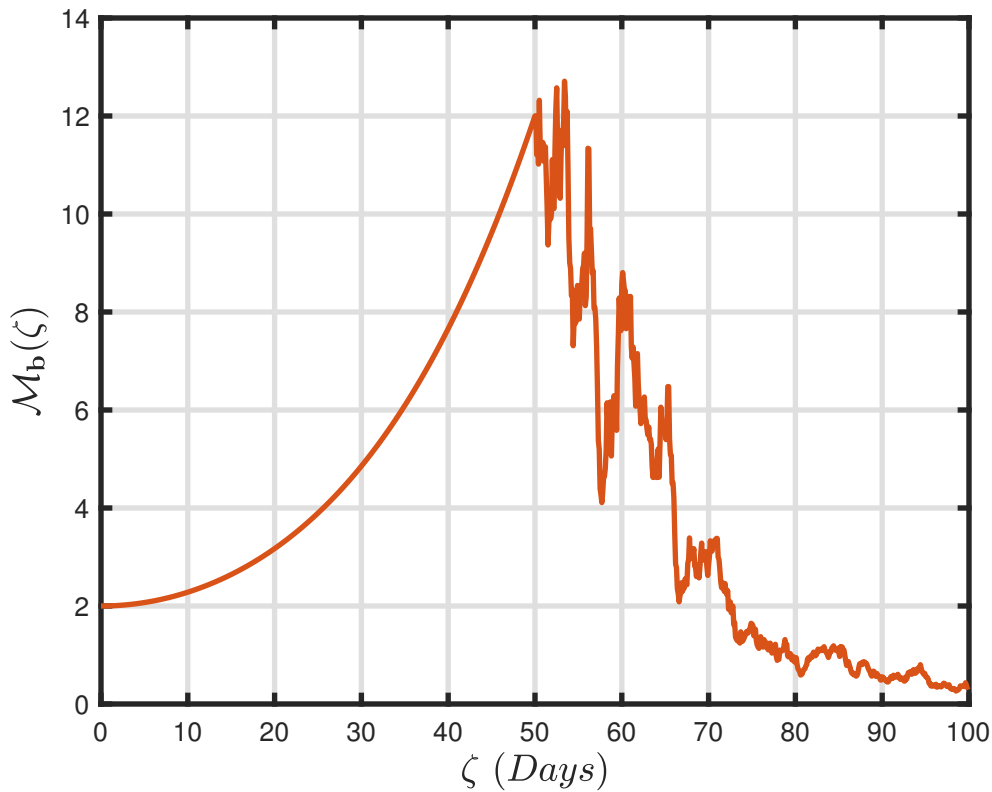

Supplement: Supplementary file 1 — Supplementary Information. [file 41598_2023_41861_MOESM1_ESM.zip › nutrution/Plot2aa-eps-converted-to.pdf]

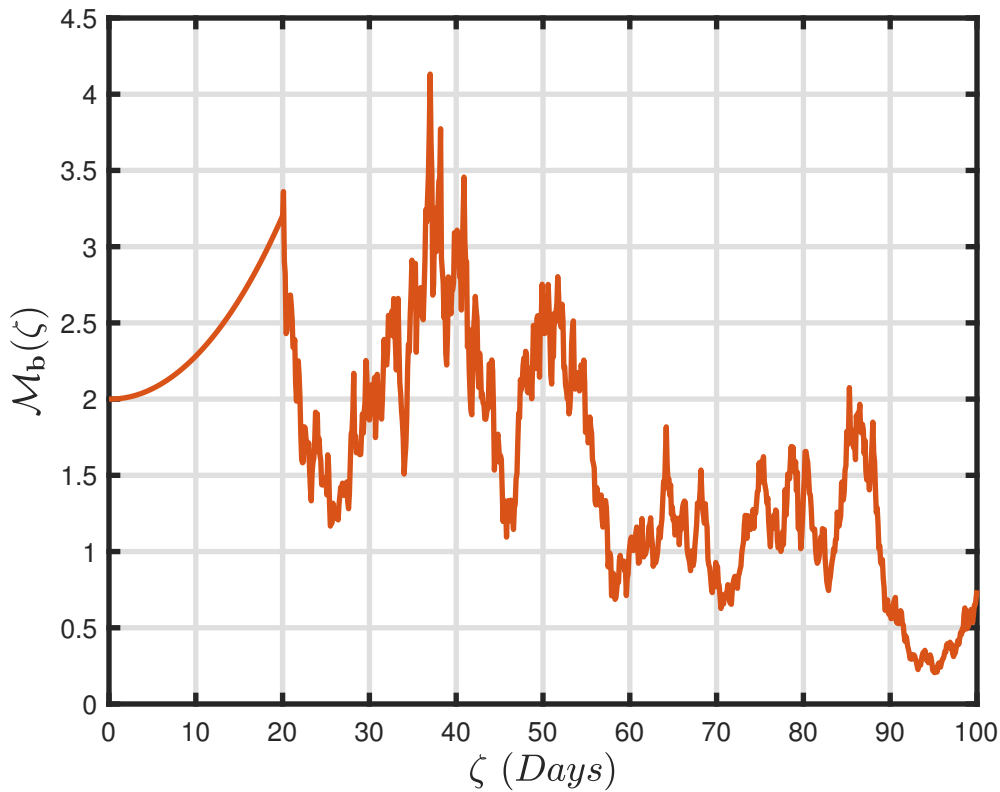

Supplement: Supplementary file 1 — Supplementary Information. [file 41598_2023_41861_MOESM1_ESM.zip › nutrution/Plot2a-eps-converted-to.pdf]

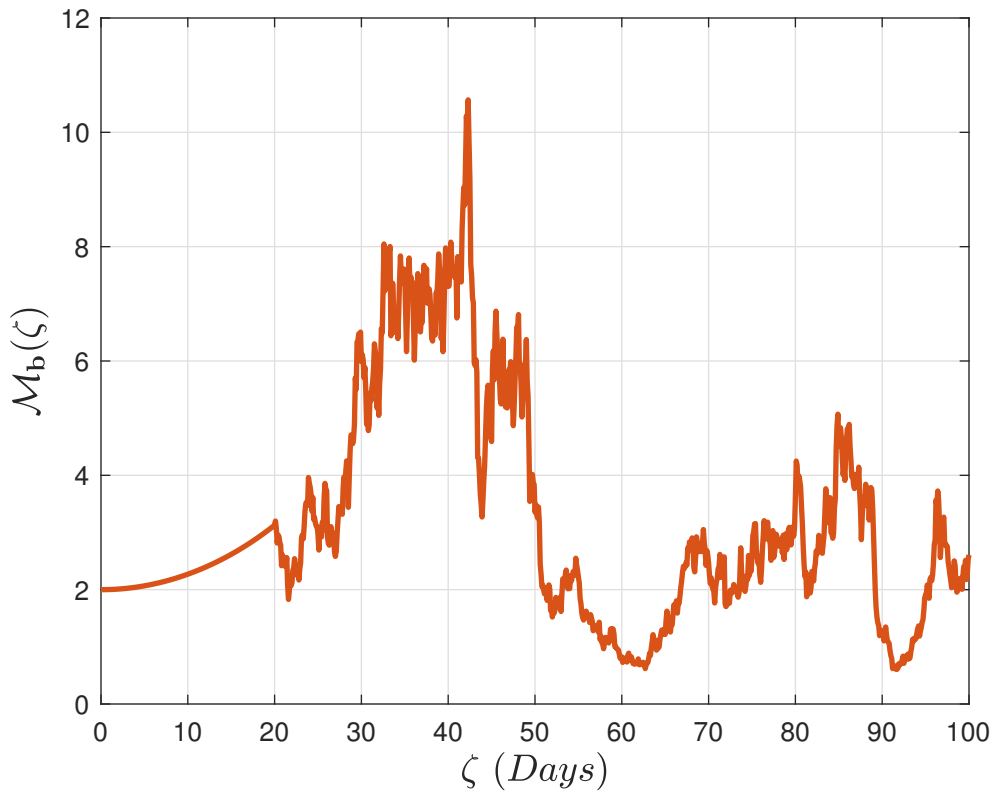

Supplement: Supplementary file 1 — Supplementary Information. [file 41598_2023_41861_MOESM1_ESM.zip › nutrution/Plot2-eps-converted-to.pdf]

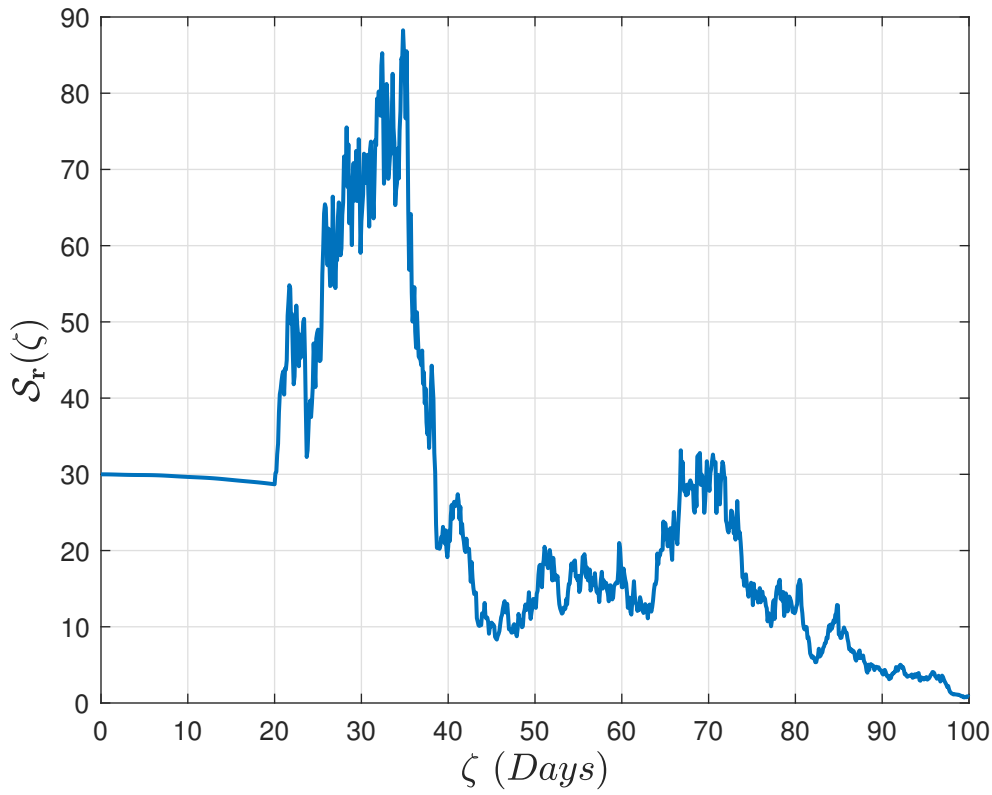

Supplement: Supplementary file 1 — Supplementary Information. [file 41598_2023_41861_MOESM1_ESM.zip › nutrution/Plot31-eps-converted-to.pdf]

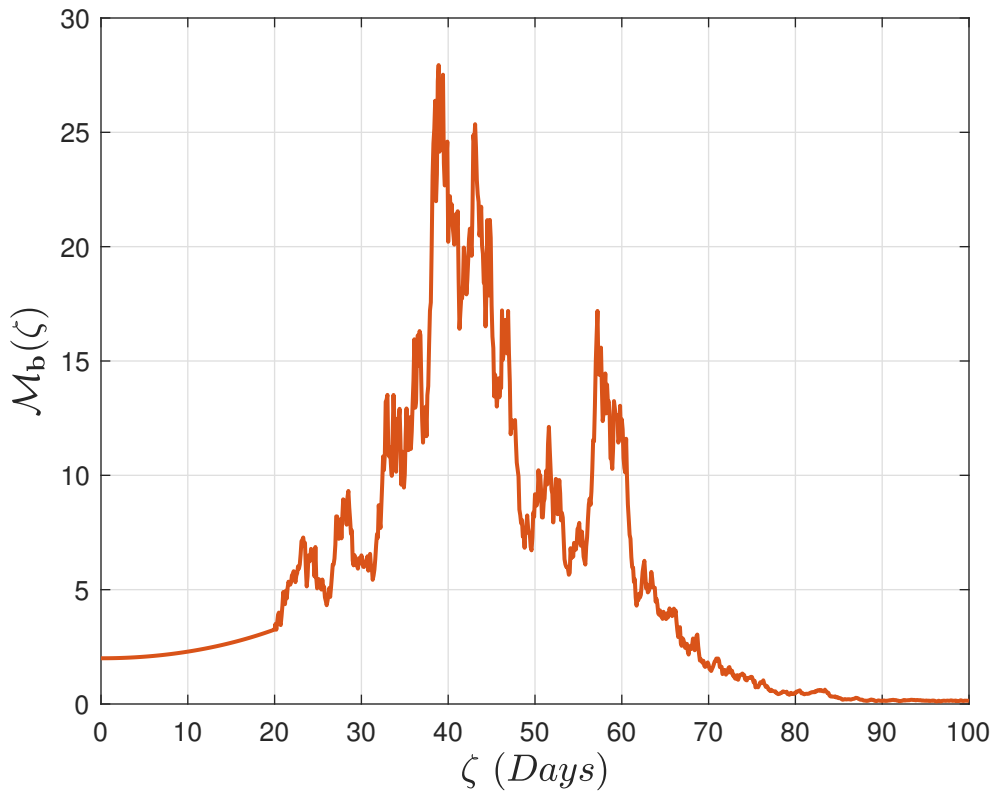

Supplement: Supplementary file 1 — Supplementary Information. [file 41598_2023_41861_MOESM1_ESM.zip › nutrution/Plot32-eps-converted-to.pdf]

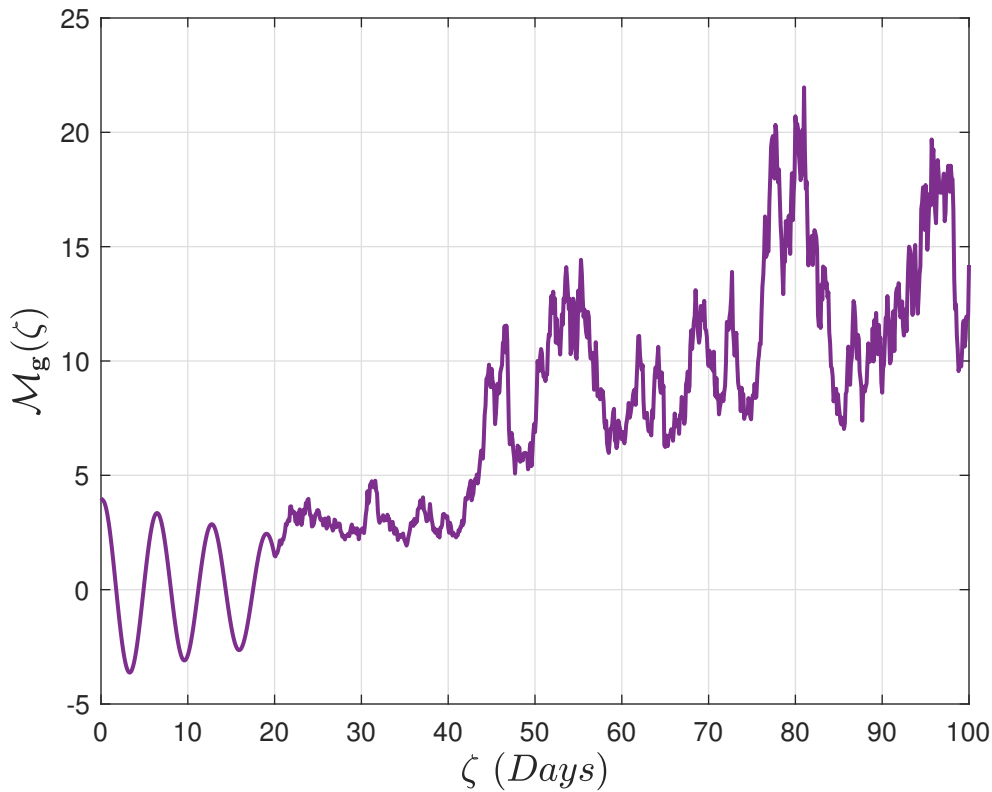

Supplement: Supplementary file 1 — Supplementary Information. [file 41598_2023_41861_MOESM1_ESM.zip › nutrution/Plot33-eps-converted-to.pdf]

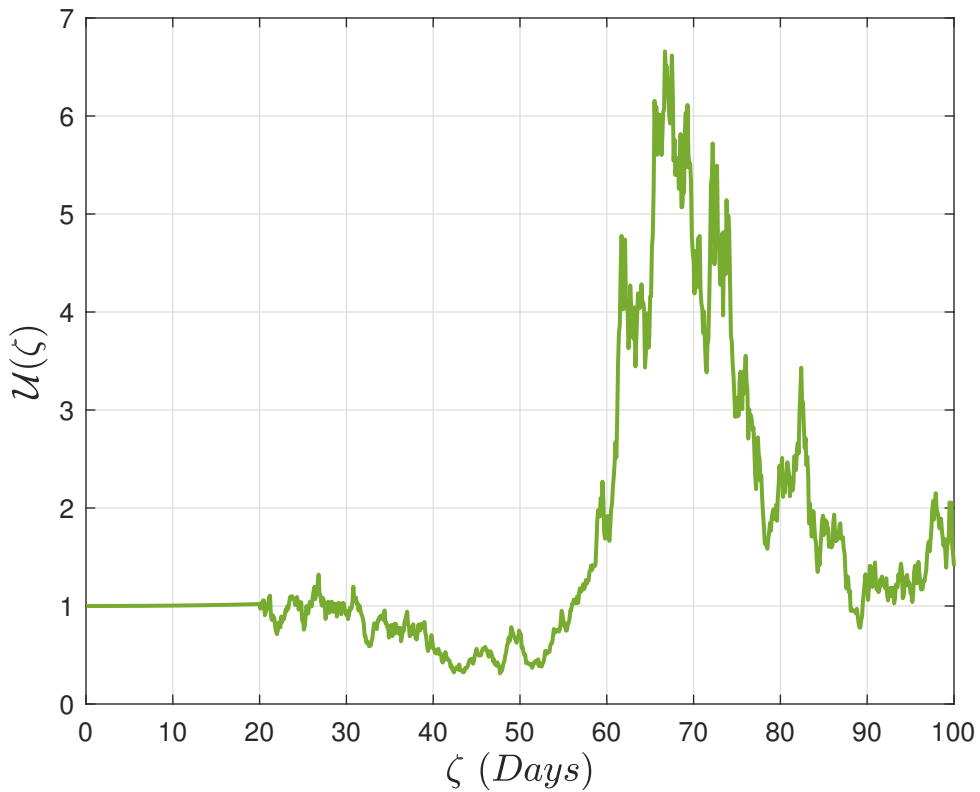

Supplement: Supplementary file 1 — Supplementary Information. [file 41598_2023_41861_MOESM1_ESM.zip › nutrution/Plot34-eps-converted-to.pdf]

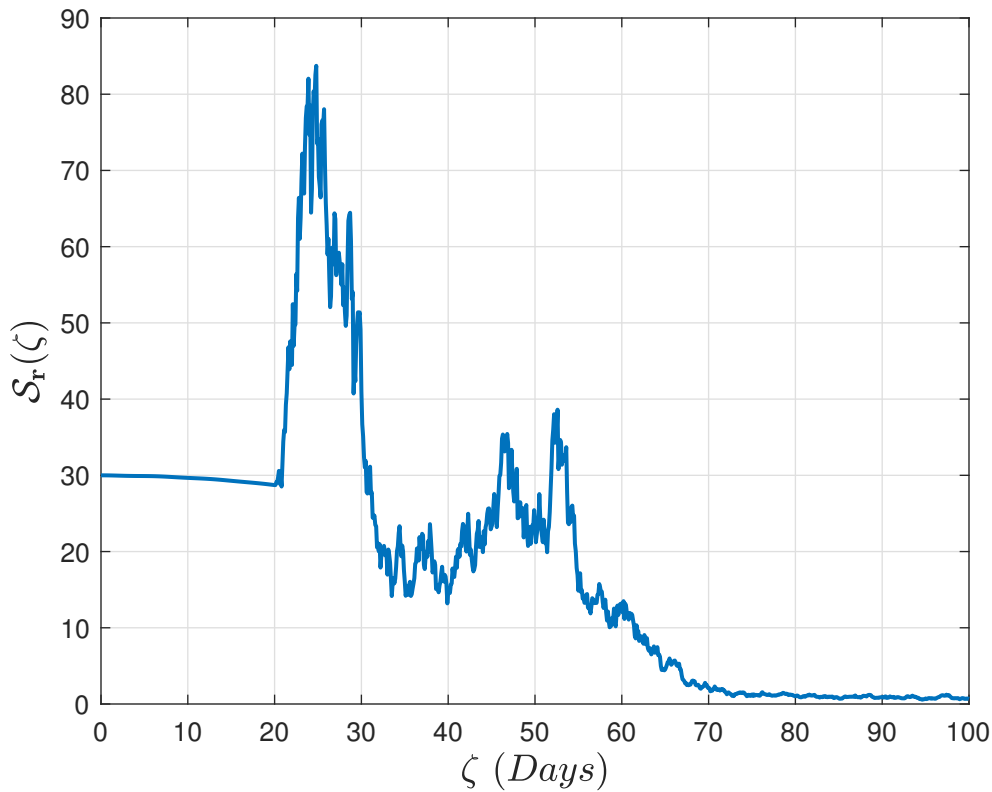

Supplement: Supplementary file 1 — Supplementary Information. [file 41598_2023_41861_MOESM1_ESM.zip › nutrution/Plot35-eps-converted-to.pdf]

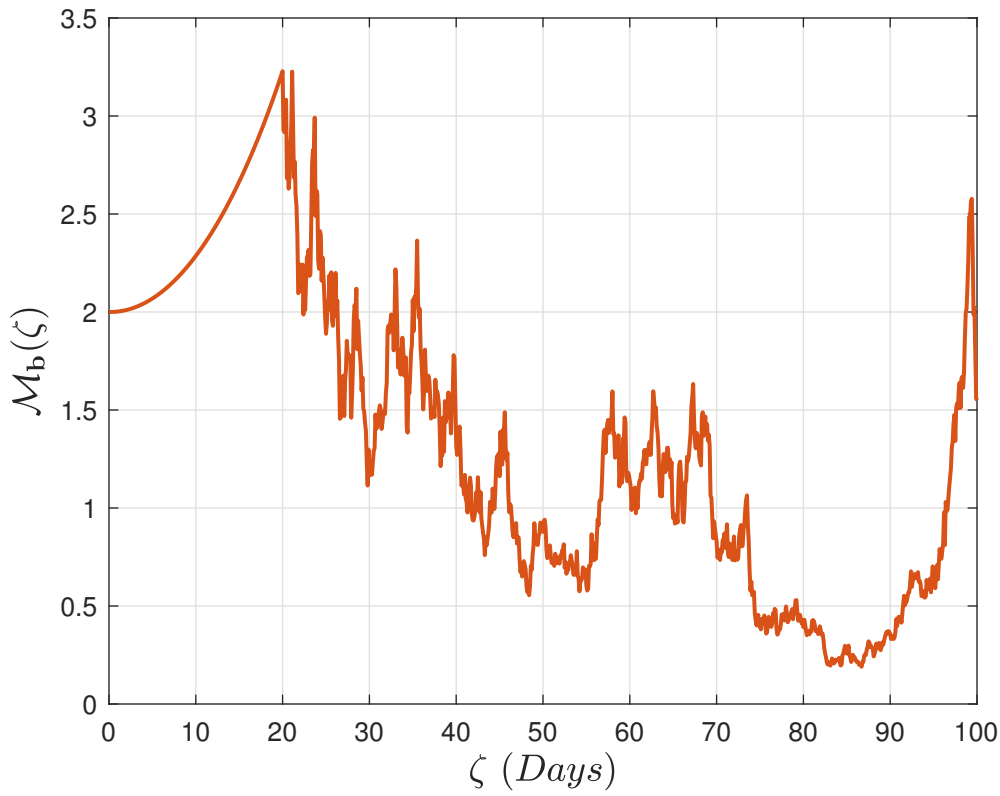

Supplement: Supplementary file 1 — Supplementary Information. [file 41598_2023_41861_MOESM1_ESM.zip › nutrution/Plot36-eps-converted-to.pdf]

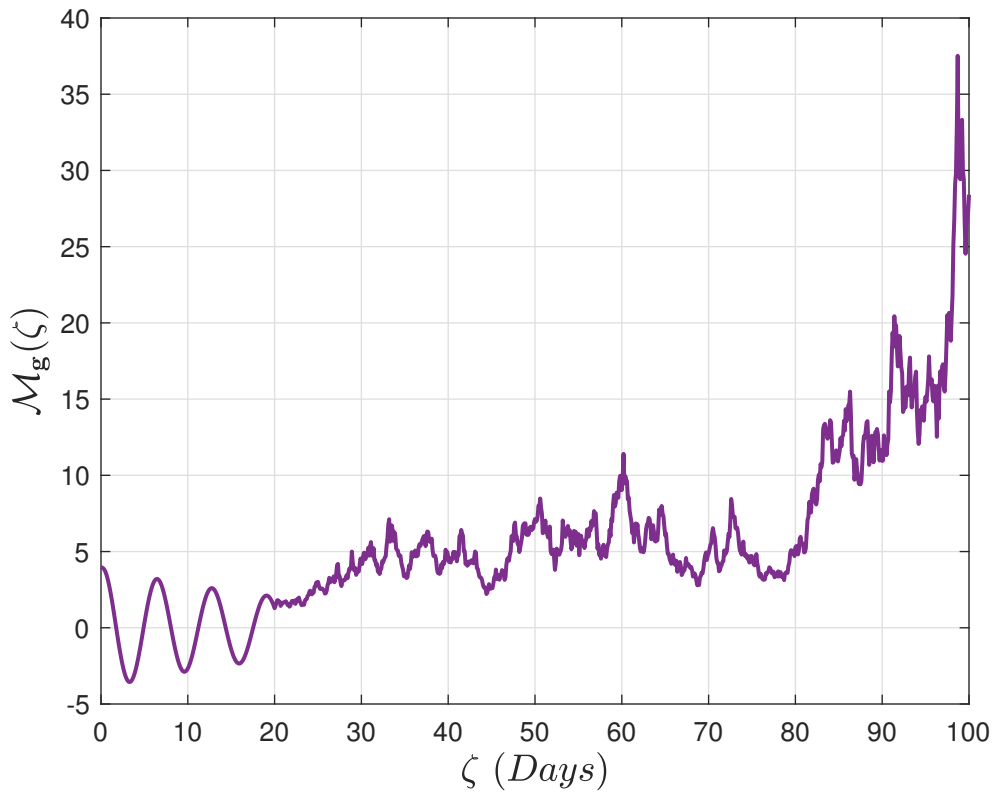

Supplement: Supplementary file 1 — Supplementary Information. [file 41598_2023_41861_MOESM1_ESM.zip › nutrution/Plot37-eps-converted-to.pdf]

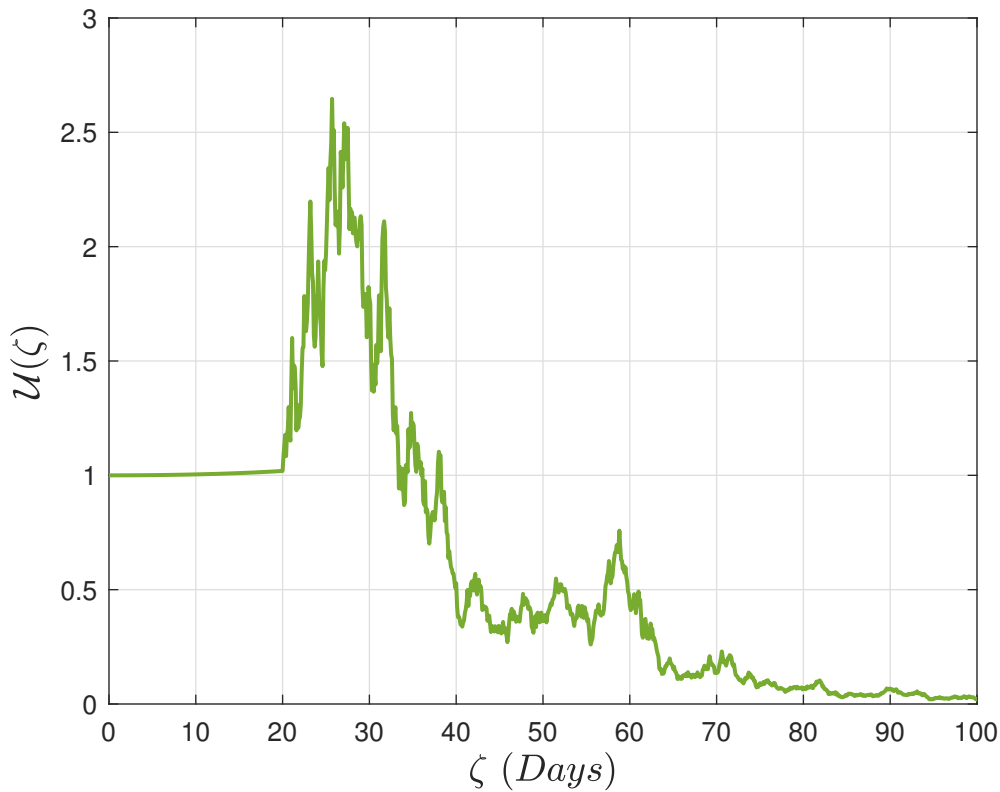

Supplement: Supplementary file 1 — Supplementary Information. [file 41598_2023_41861_MOESM1_ESM.zip › nutrution/Plot38-eps-converted-to.pdf]

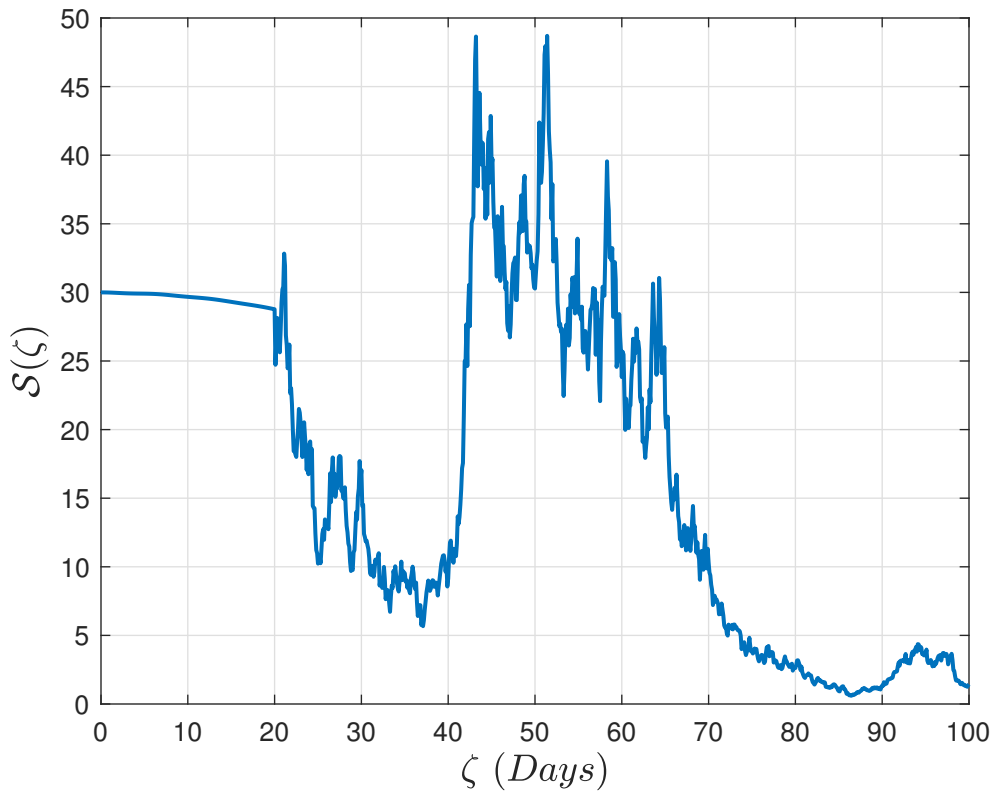

Supplement: Supplementary file 1 — Supplementary Information. [file 41598_2023_41861_MOESM1_ESM.zip › nutrution/Plot39-eps-converted-to.pdf]

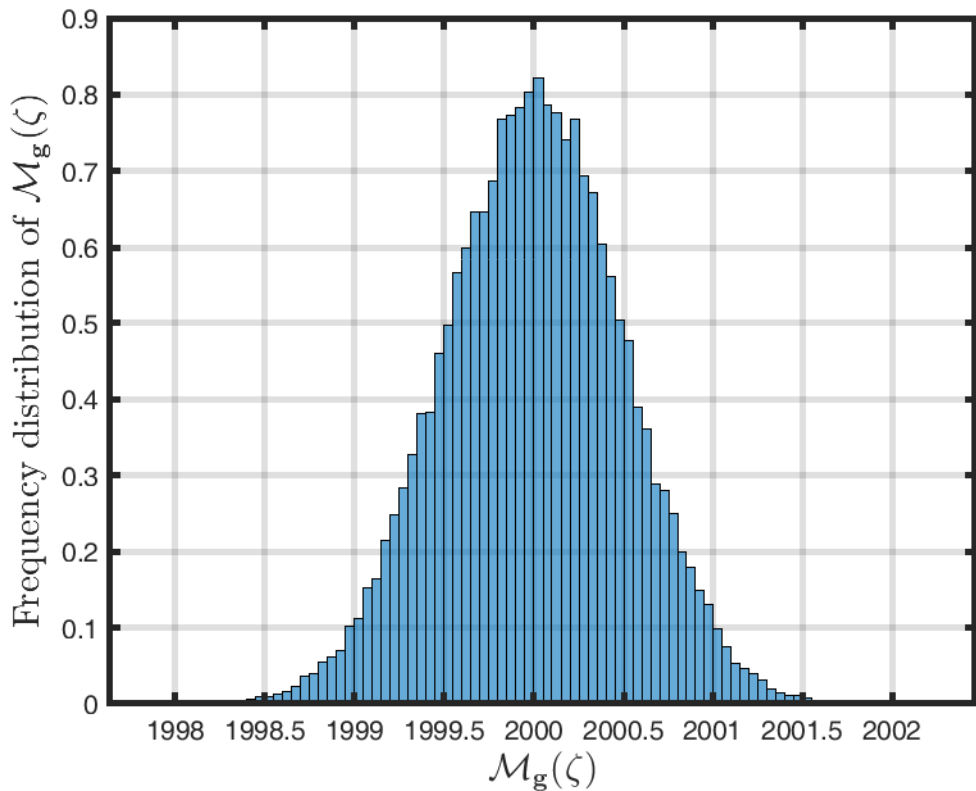

Supplement: Supplementary file 1 — Supplementary Information. [file 41598_2023_41861_MOESM1_ESM.zip › nutrution/Plot3aaaa-eps-converted-to.pdf]

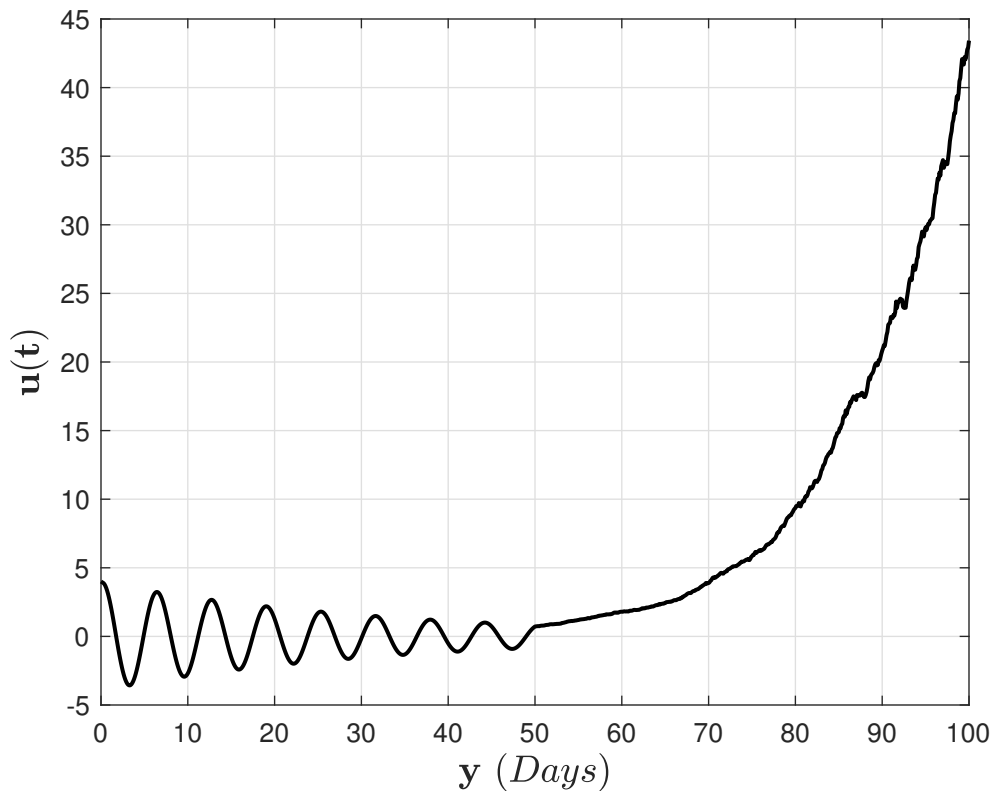

Supplement: Supplementary file 1 — Supplementary Information. [file 41598_2023_41861_MOESM1_ESM.zip › nutrution/Plot3aaa-eps-converted-to.pdf]

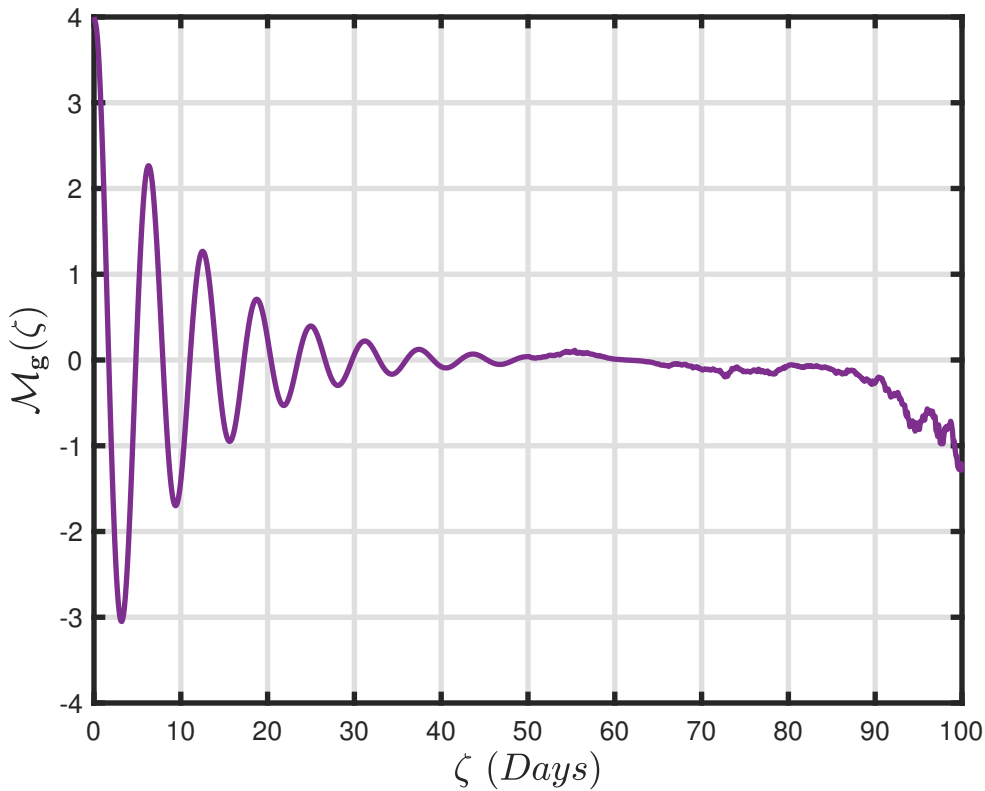

Supplement: Supplementary file 1 — Supplementary Information. [file 41598_2023_41861_MOESM1_ESM.zip › nutrution/Plot3aa-eps-converted-to.pdf]

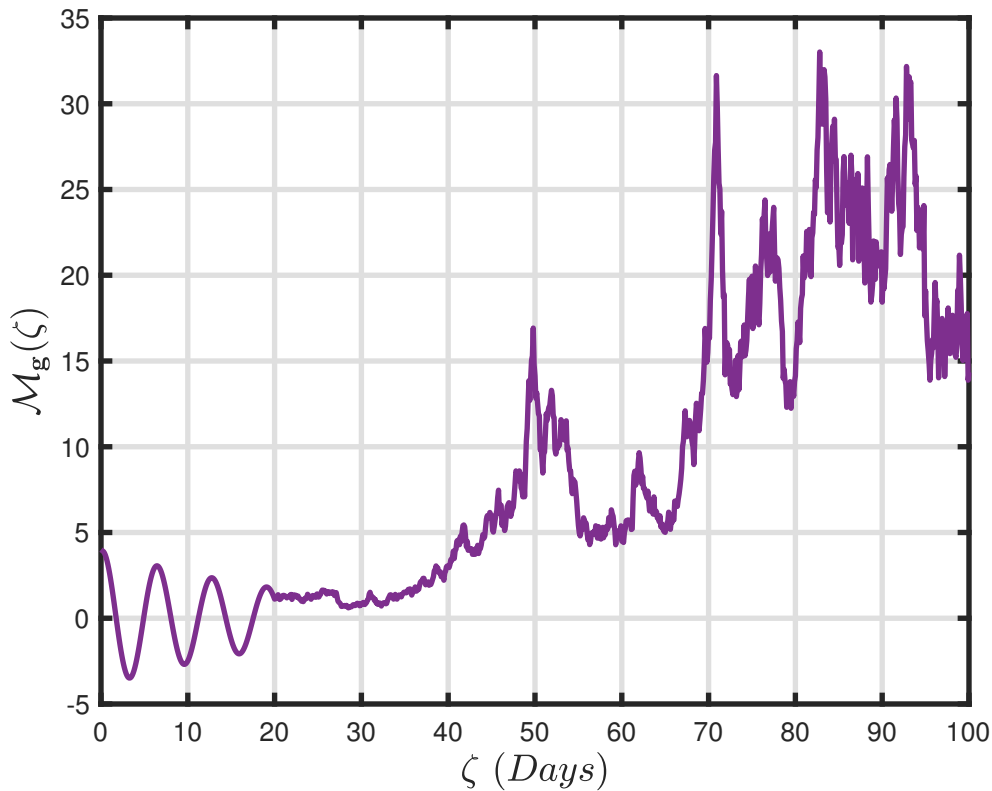

Supplement: Supplementary file 1 — Supplementary Information. [file 41598_2023_41861_MOESM1_ESM.zip › nutrution/Plot3a-eps-converted-to.pdf]

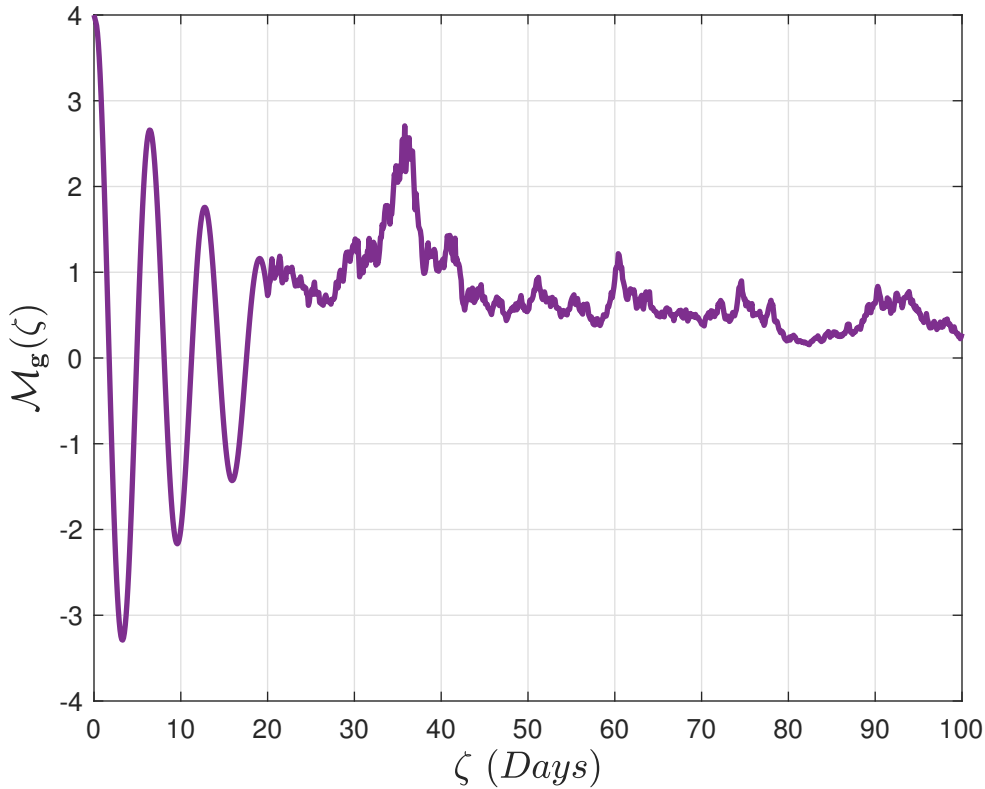

Supplement: Supplementary file 1 — Supplementary Information. [file 41598_2023_41861_MOESM1_ESM.zip › nutrution/Plot3-eps-converted-to.pdf]

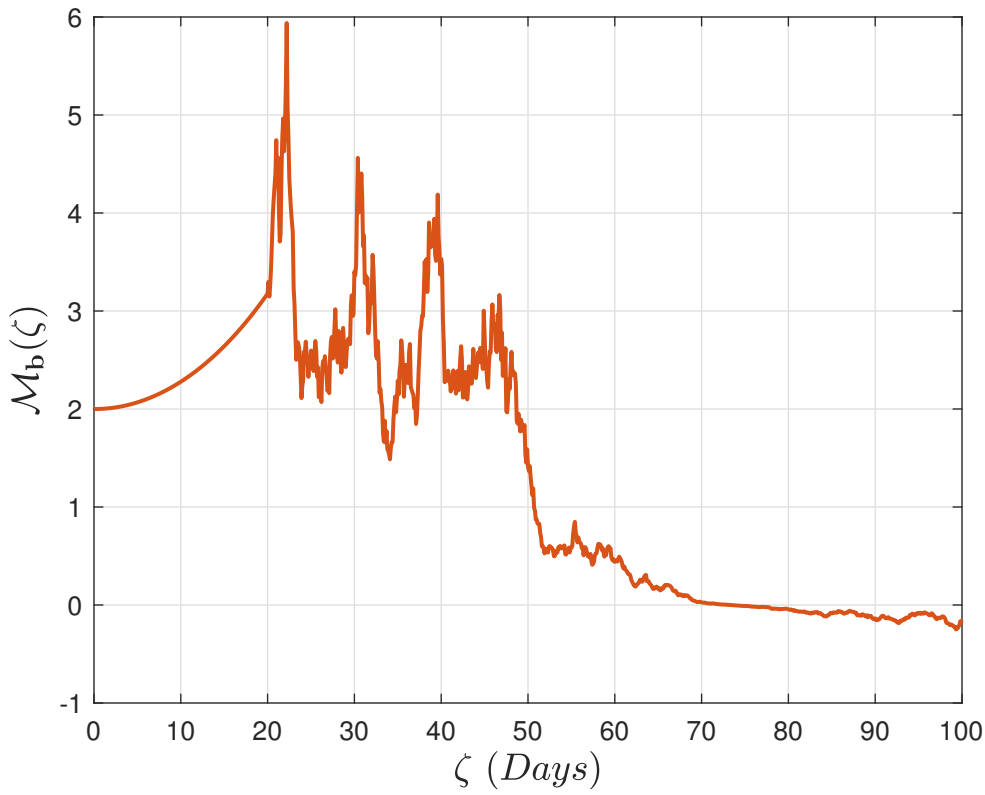

Supplement: Supplementary file 1 — Supplementary Information. [file 41598_2023_41861_MOESM1_ESM.zip › nutrution/Plot40-eps-converted-to.pdf]

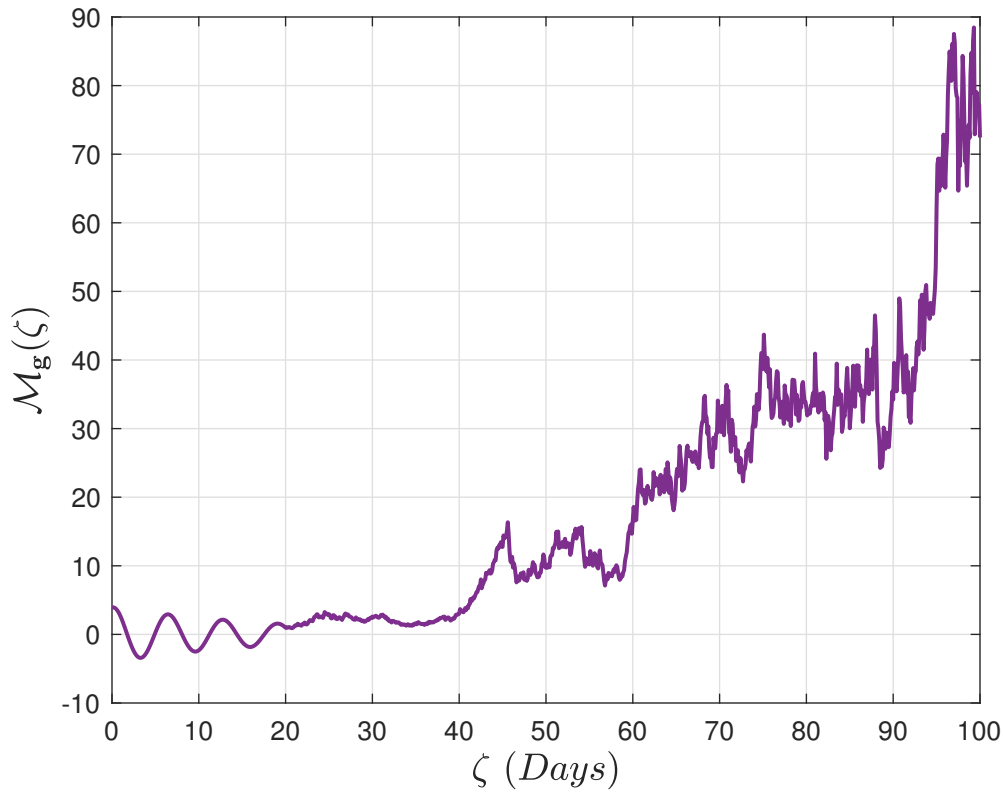

Supplement: Supplementary file 1 — Supplementary Information. [file 41598_2023_41861_MOESM1_ESM.zip › nutrution/Plot41-eps-converted-to.pdf]

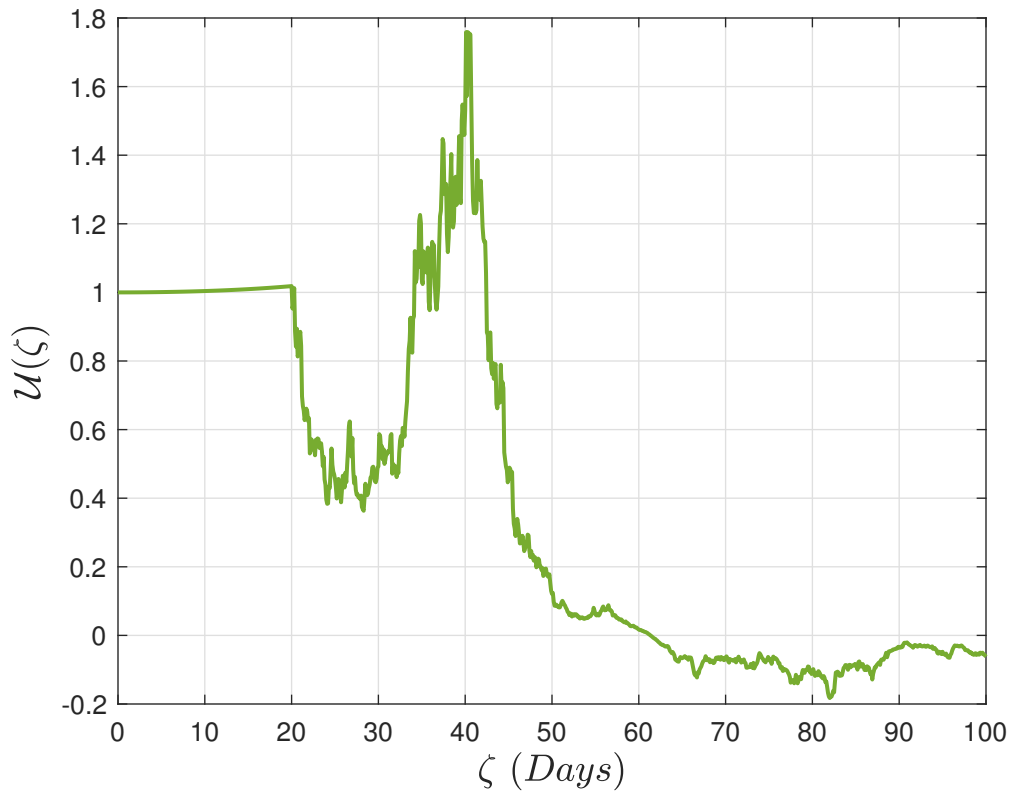

Supplement: Supplementary file 1 — Supplementary Information. [file 41598_2023_41861_MOESM1_ESM.zip › nutrution/Plot42-eps-converted-to.pdf]

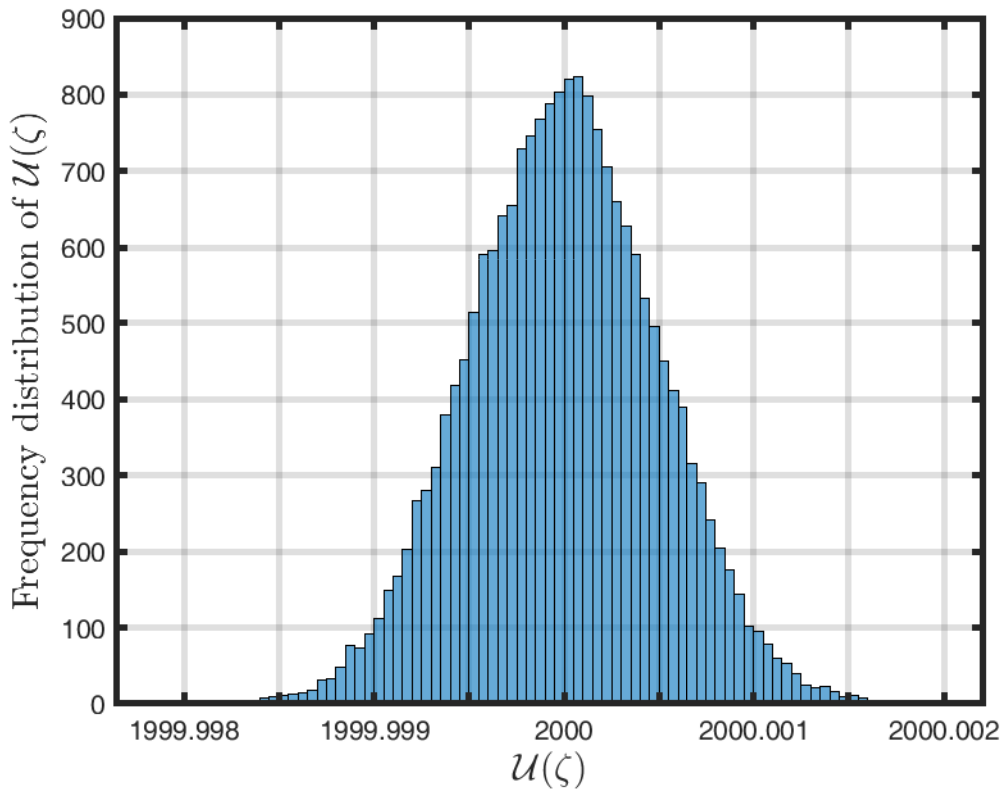

Supplement: Supplementary file 1 — Supplementary Information. [file 41598_2023_41861_MOESM1_ESM.zip › nutrution/Plot4aaaa-eps-converted-to.pdf]

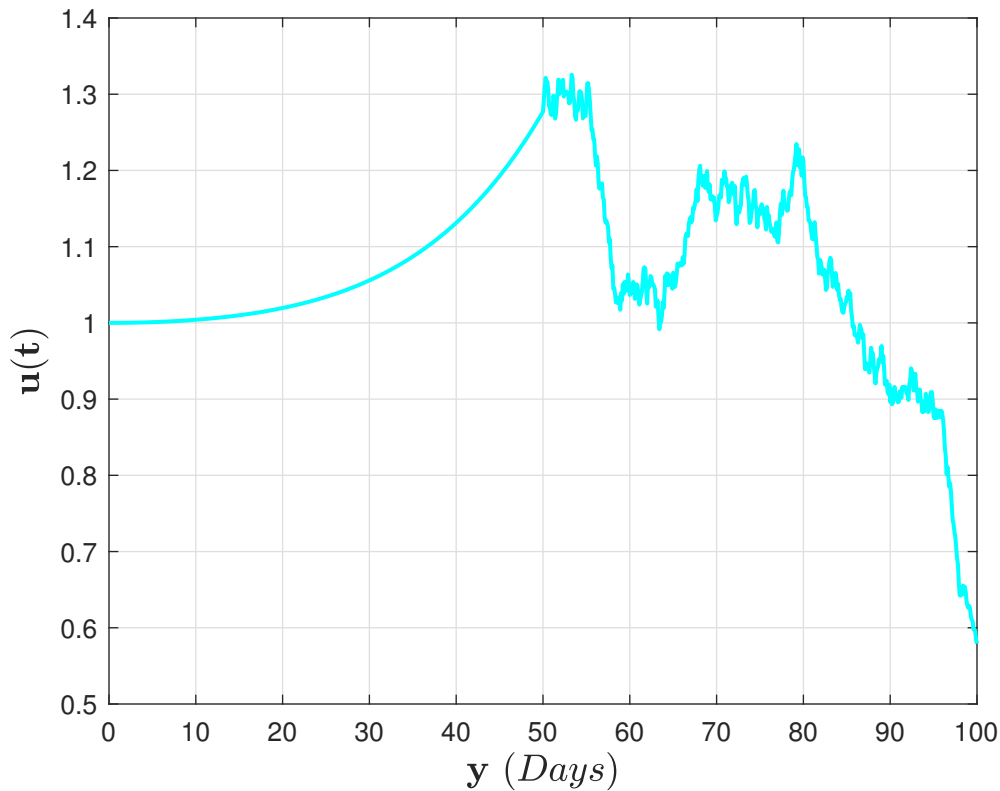

Supplement: Supplementary file 1 — Supplementary Information. [file 41598_2023_41861_MOESM1_ESM.zip › nutrution/Plot4aaa-eps-converted-to.pdf]

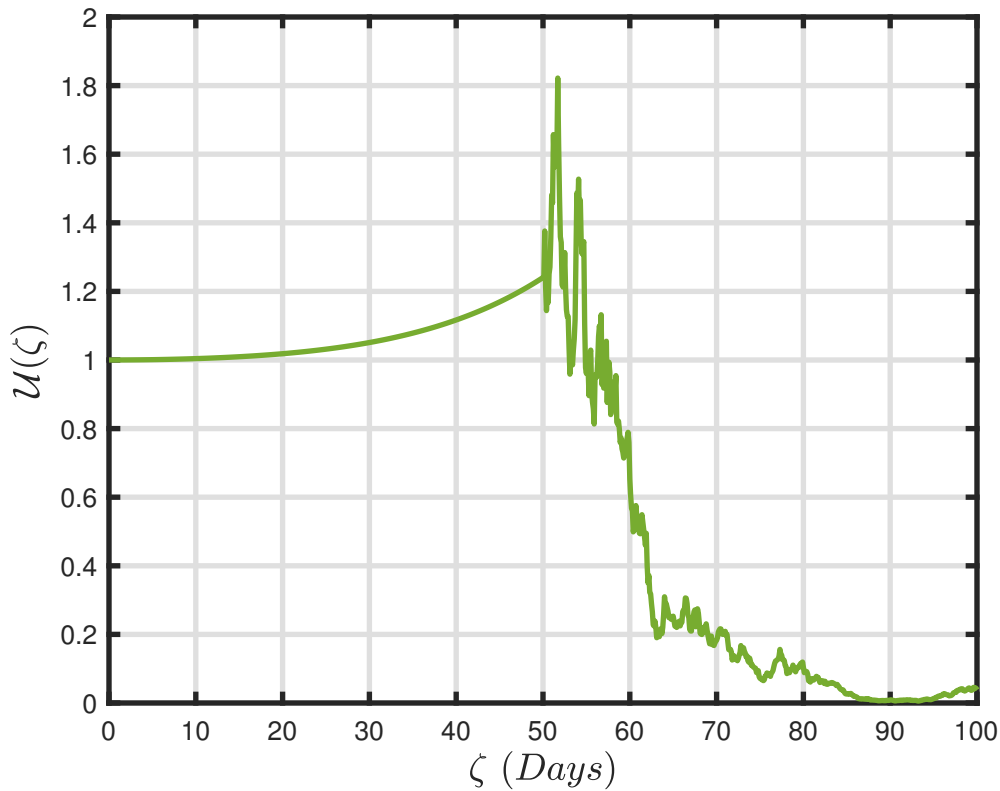

Supplement: Supplementary file 1 — Supplementary Information. [file 41598_2023_41861_MOESM1_ESM.zip › nutrution/Plot4aa-eps-converted-to.pdf]

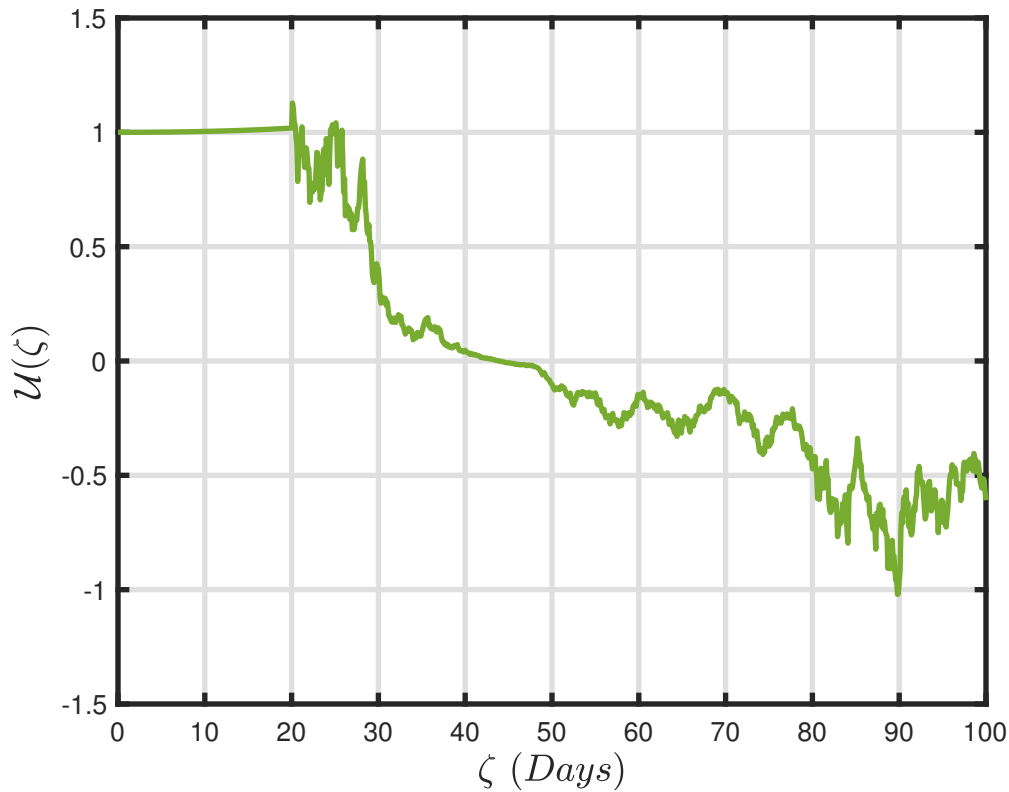

Supplement: Supplementary file 1 — Supplementary Information. [file 41598_2023_41861_MOESM1_ESM.zip › nutrution/Plot4a-eps-converted-to.pdf]

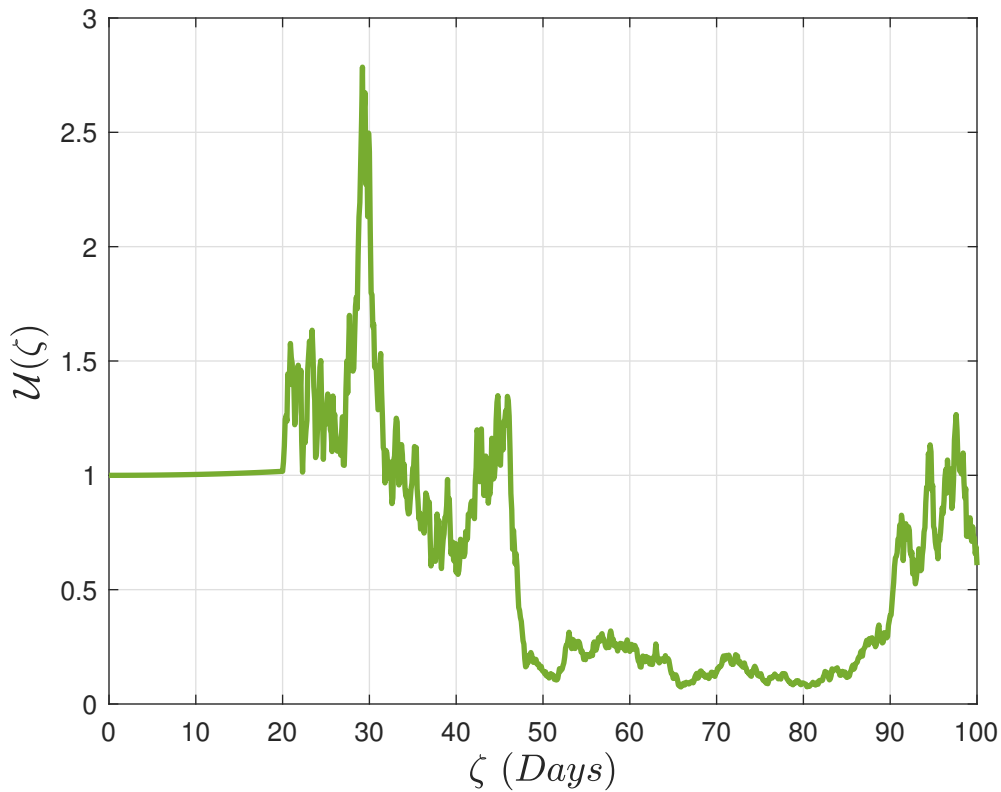

Supplement: Supplementary file 1 — Supplementary Information. [file 41598_2023_41861_MOESM1_ESM.zip › nutrution/Plot4-eps-converted-to.pdf]

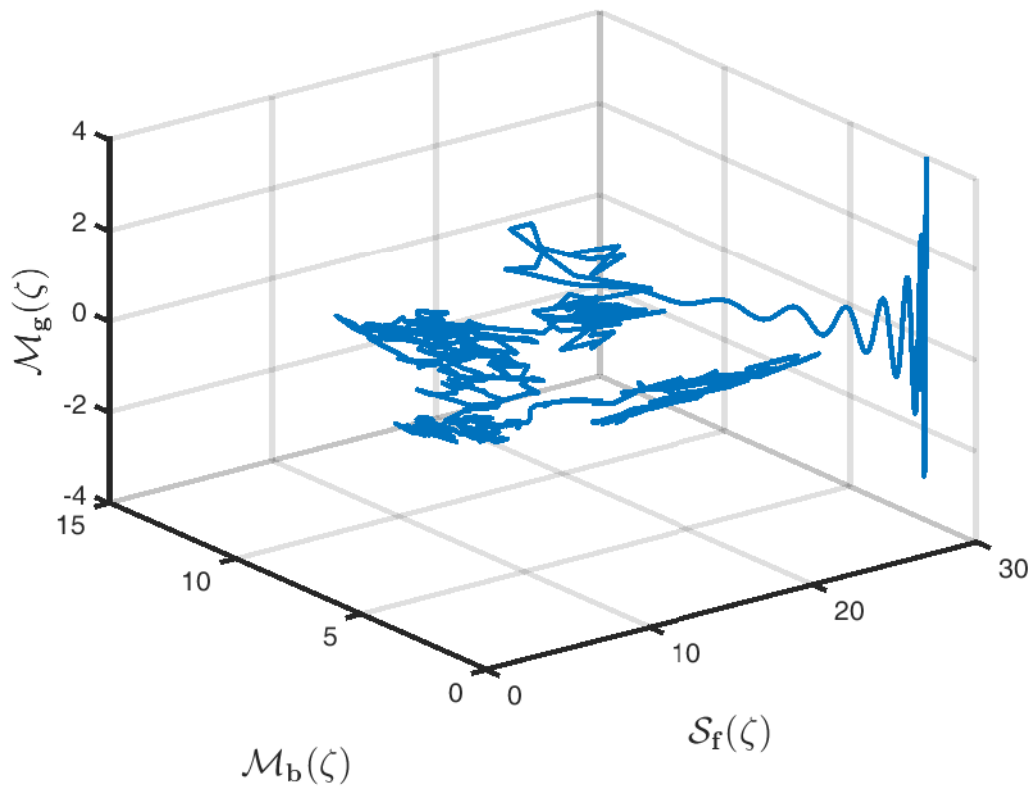

Supplement: Supplementary file 1 — Supplementary Information. [file 41598_2023_41861_MOESM1_ESM.zip › nutrution/Plot5aa-eps-converted-to.pdf]

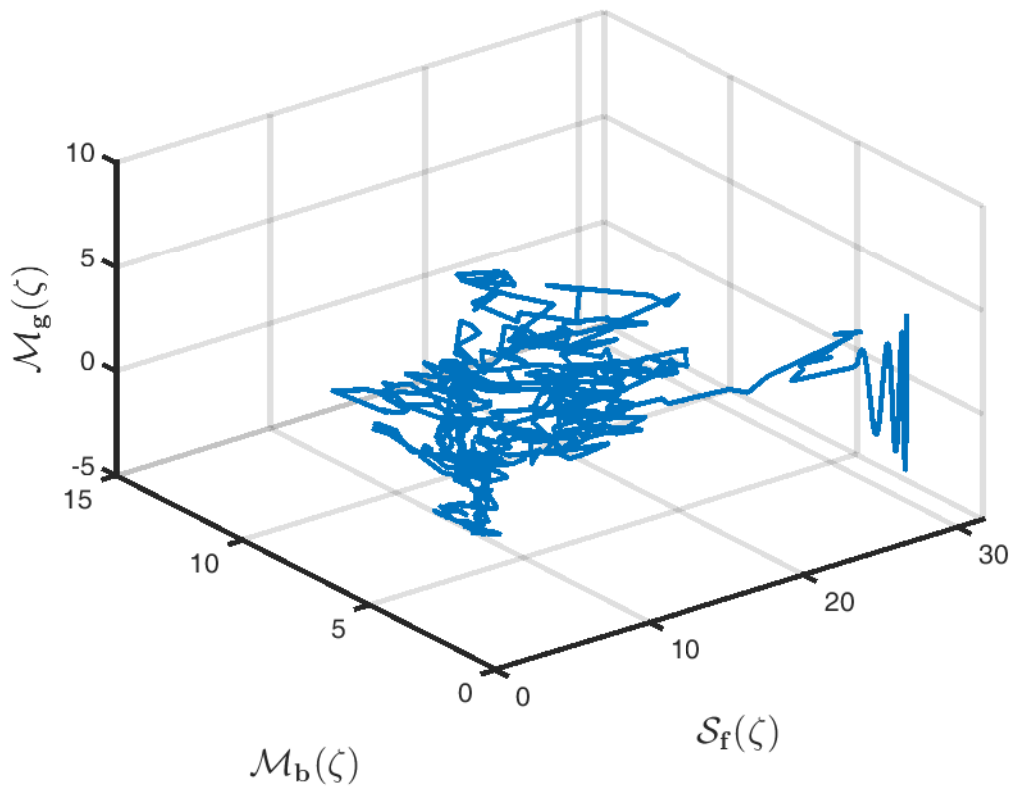

Supplement: Supplementary file 1 — Supplementary Information. [file 41598_2023_41861_MOESM1_ESM.zip › nutrution/Plot5a-eps-converted-to.pdf]

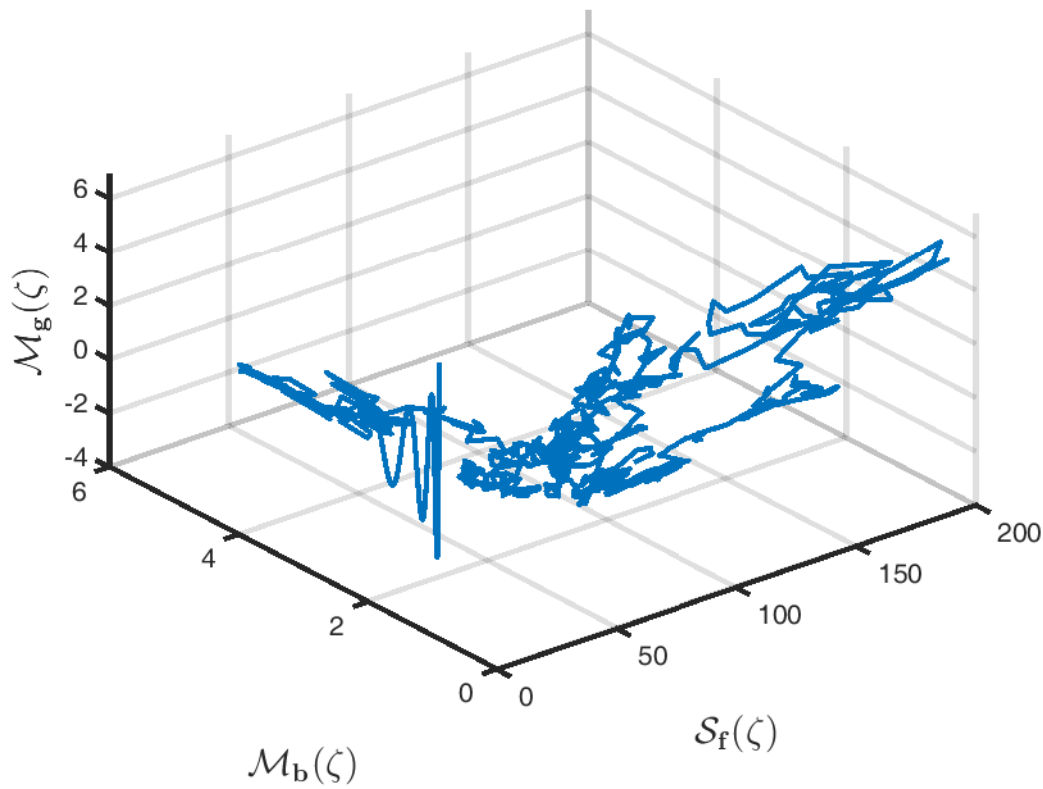

Supplement: Supplementary file 1 — Supplementary Information. [file 41598_2023_41861_MOESM1_ESM.zip › nutrution/Plot5-eps-converted-to.pdf]

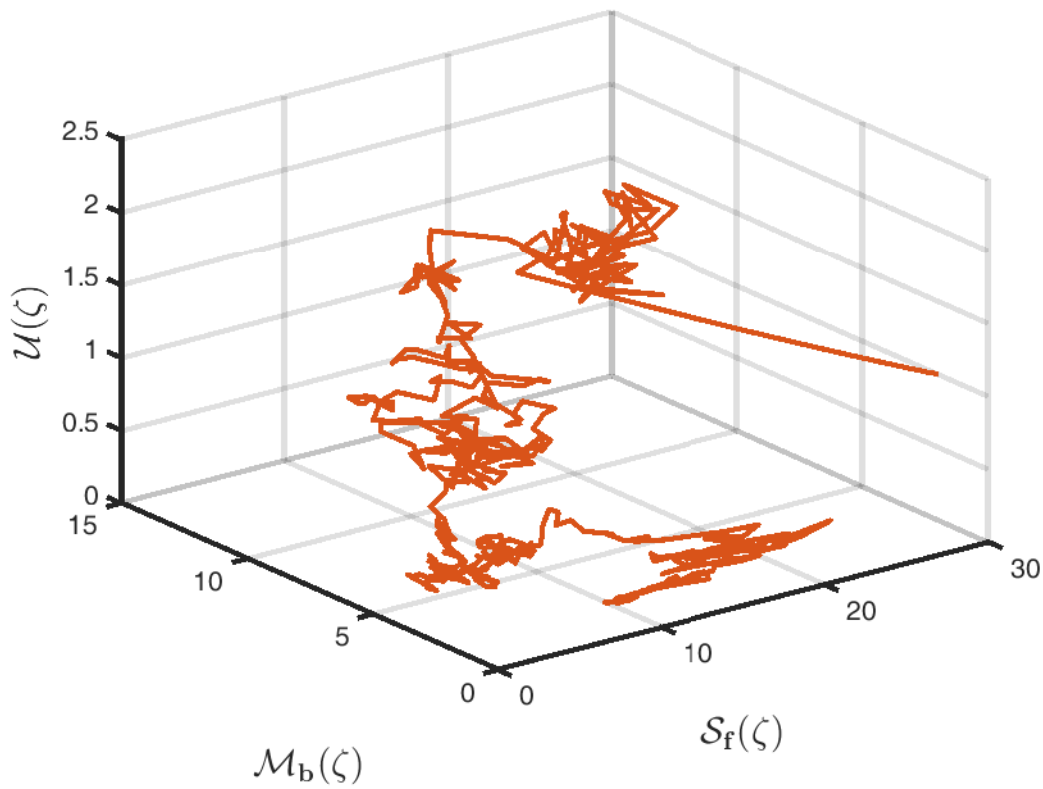

Supplement: Supplementary file 1 — Supplementary Information. [file 41598_2023_41861_MOESM1_ESM.zip › nutrution/Plot6aa-eps-converted-to.pdf]

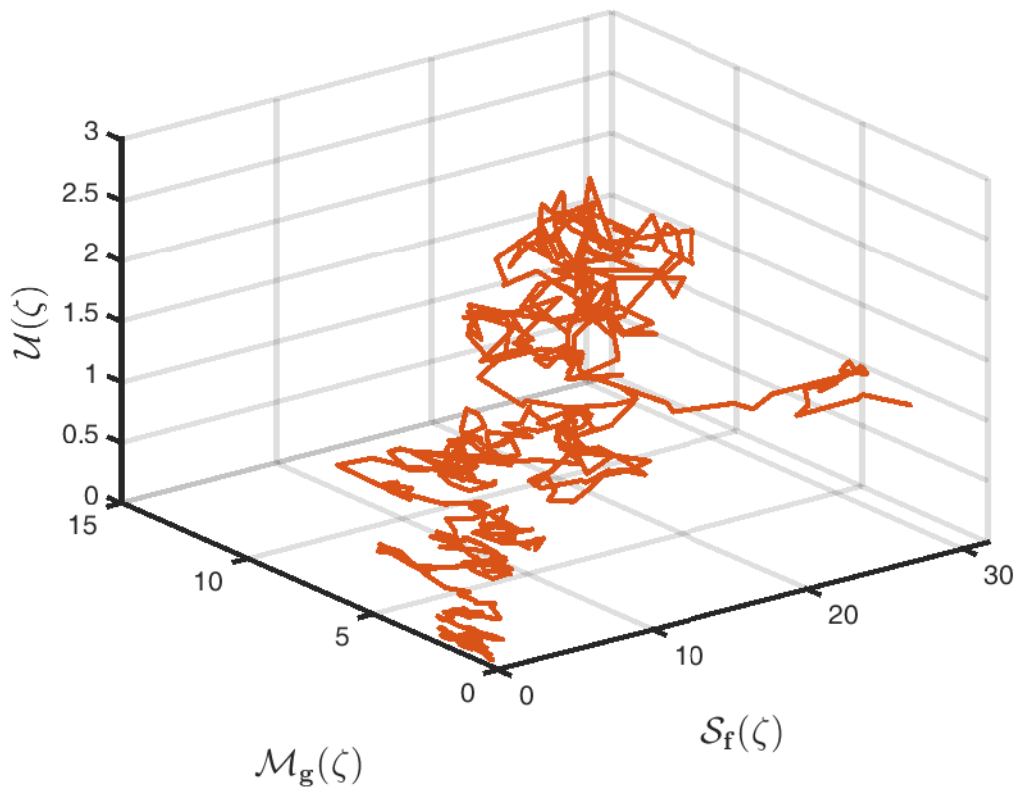

Supplement: Supplementary file 1 — Supplementary Information. [file 41598_2023_41861_MOESM1_ESM.zip › nutrution/Plot6a-eps-converted-to.pdf]

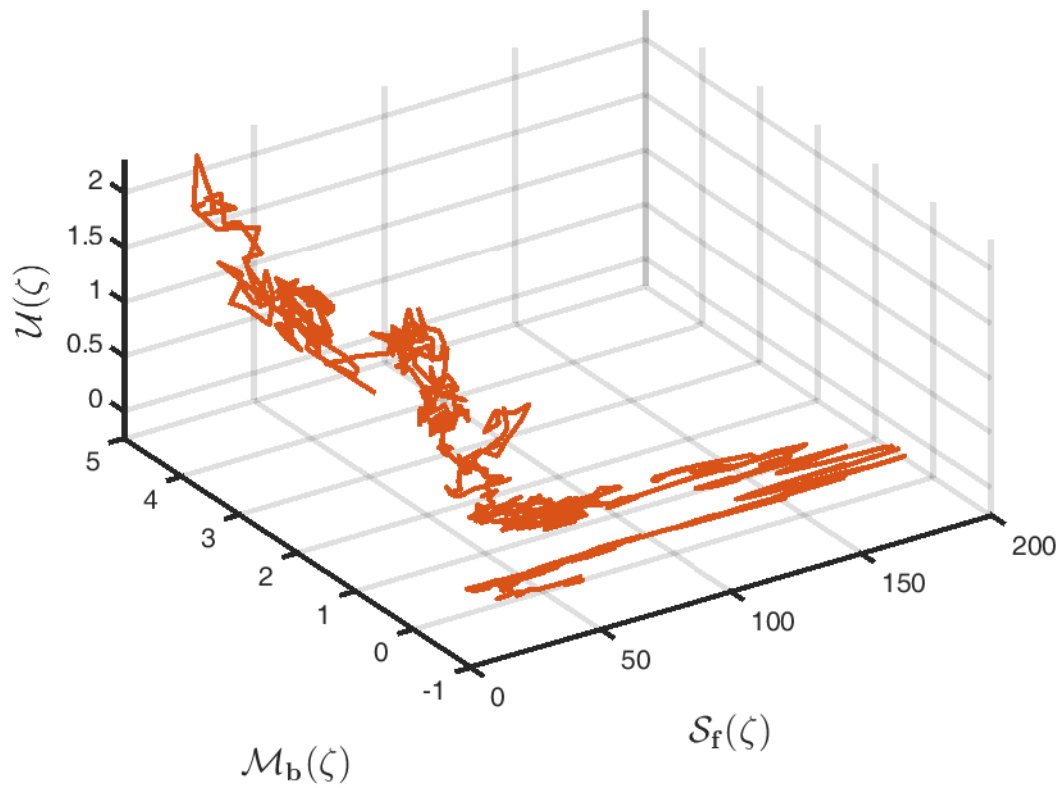

Supplement: Supplementary file 1 — Supplementary Information. [file 41598_2023_41861_MOESM1_ESM.zip › nutrution/Plot6-eps-converted-to.pdf]

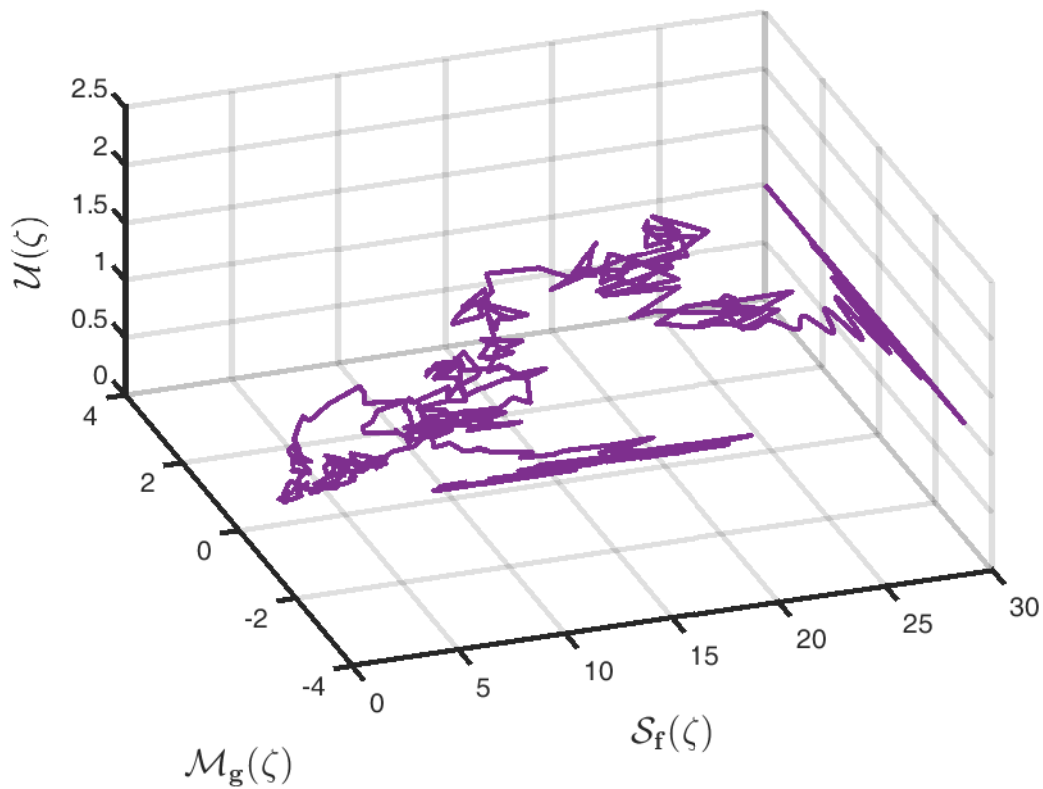

Supplement: Supplementary file 1 — Supplementary Information. [file 41598_2023_41861_MOESM1_ESM.zip › nutrution/Plot7aa-eps-converted-to.pdf]

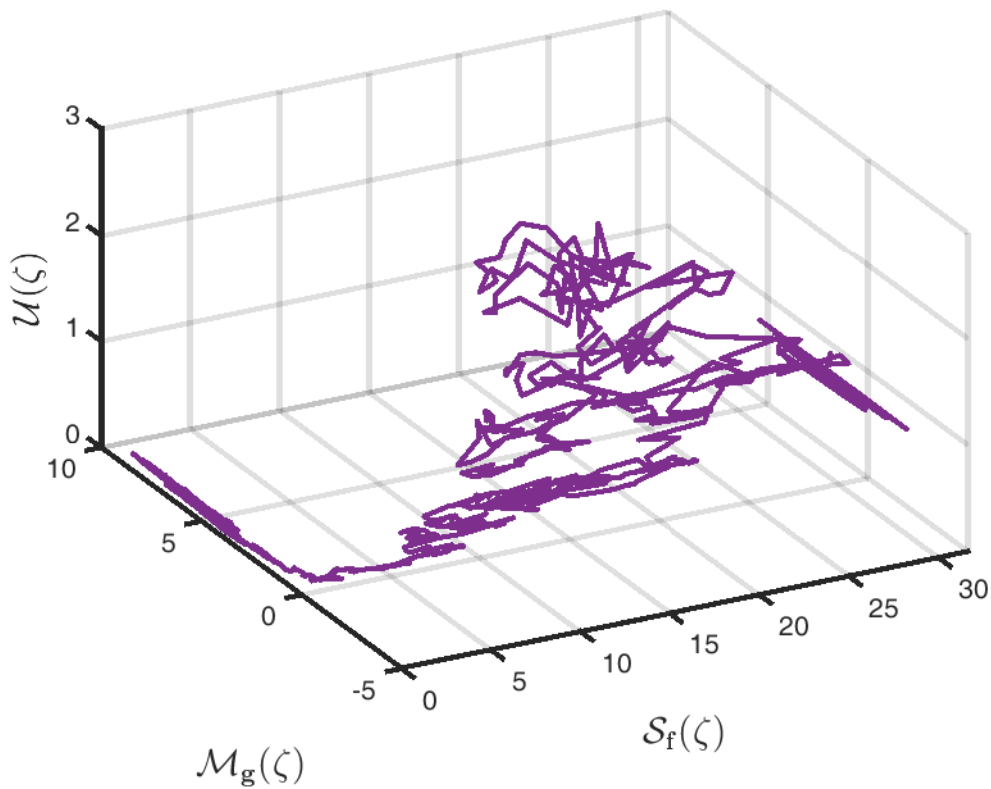

Supplement: Supplementary file 1 — Supplementary Information. [file 41598_2023_41861_MOESM1_ESM.zip › nutrution/Plot7a-eps-converted-to.pdf]

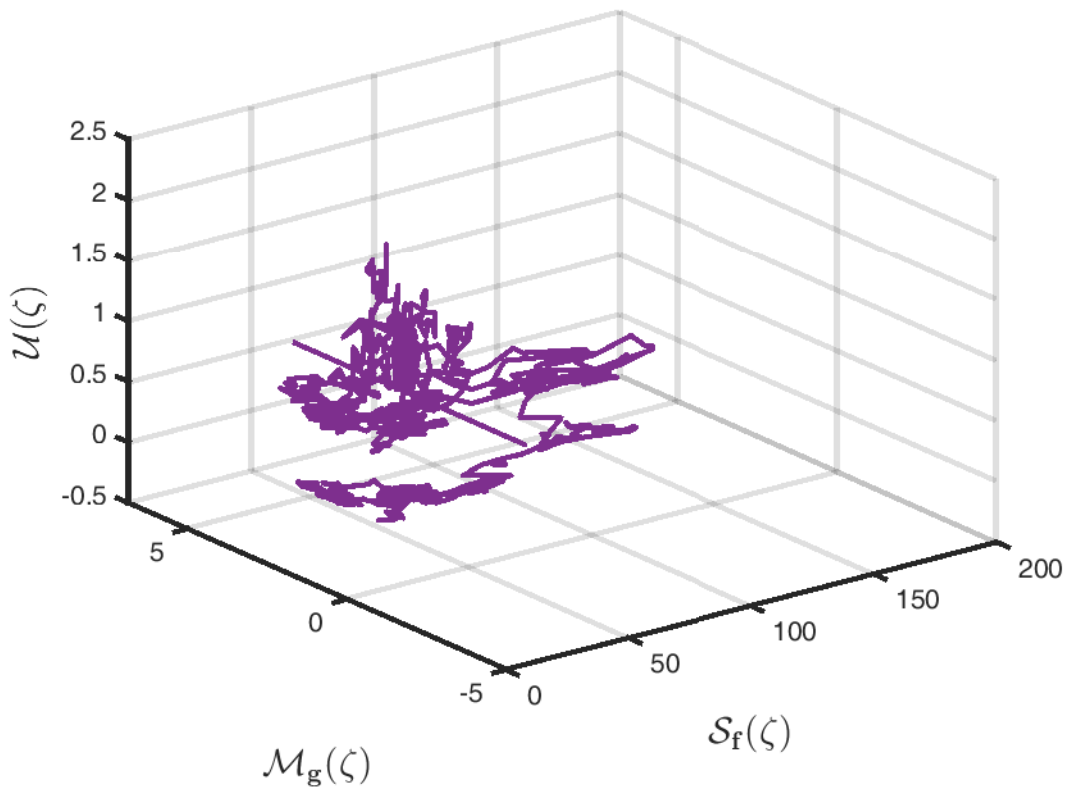

Supplement: Supplementary file 1 — Supplementary Information. [file 41598_2023_41861_MOESM1_ESM.zip › nutrution/Plot7-eps-converted-to.pdf]

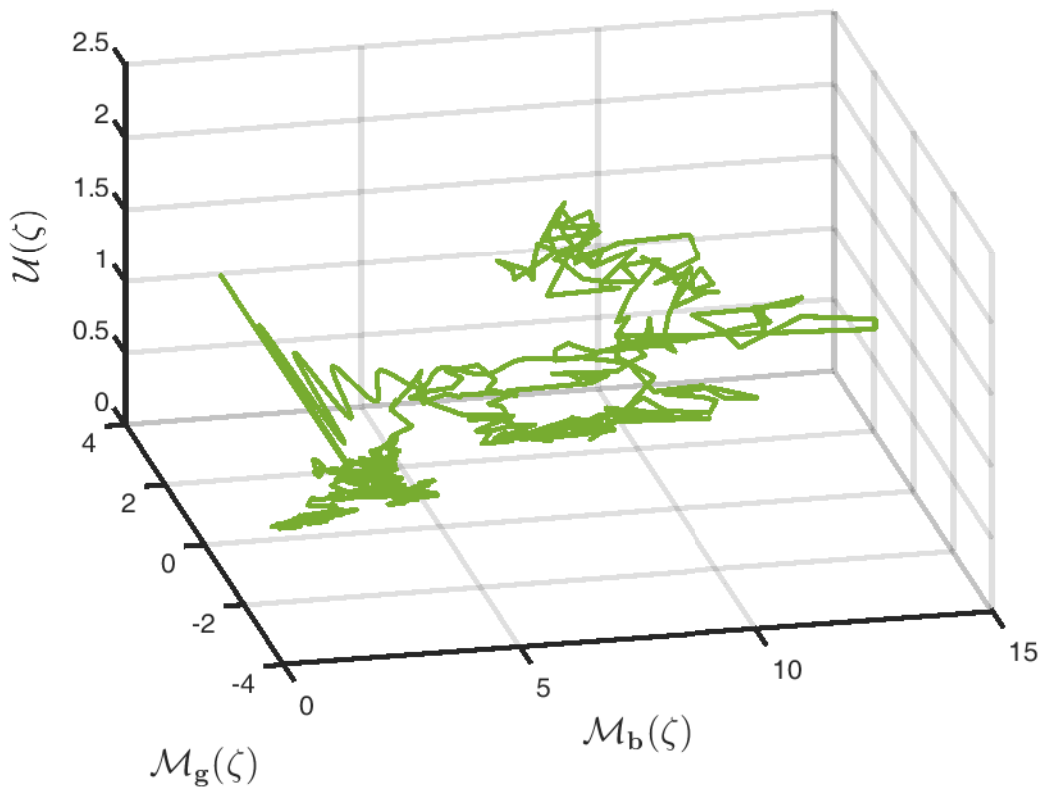

Supplement: Supplementary file 1 — Supplementary Information. [file 41598_2023_41861_MOESM1_ESM.zip › nutrution/Plot8aa-eps-converted-to.pdf]

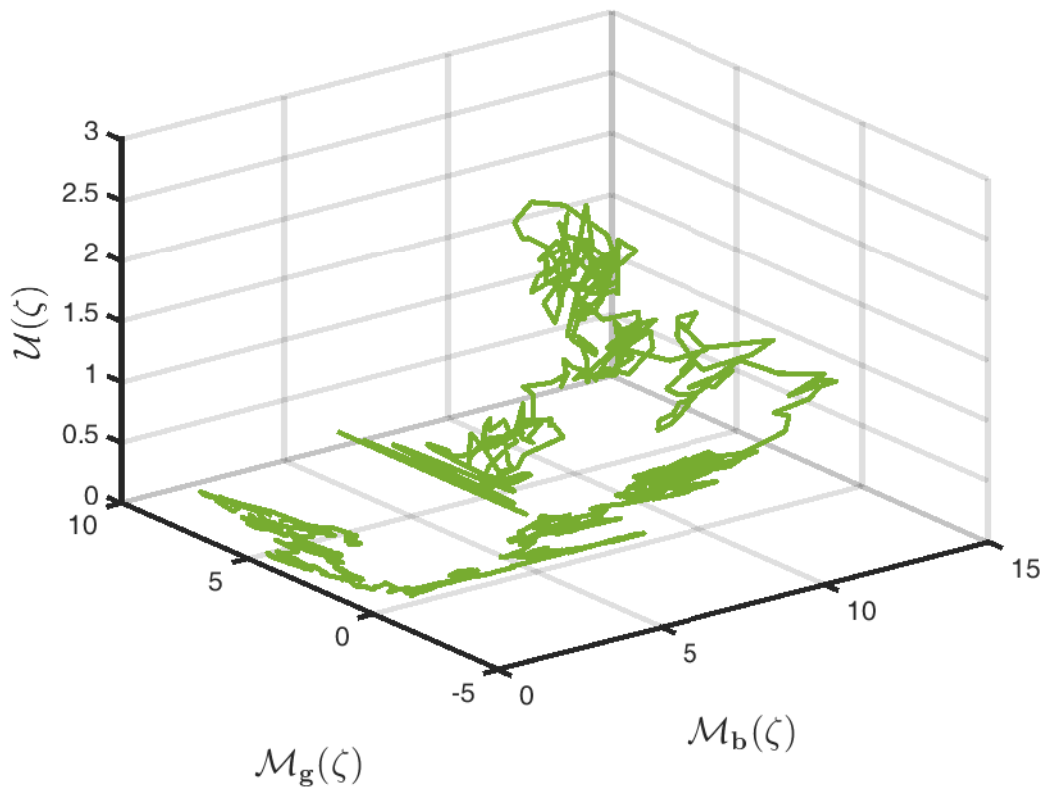

Supplement: Supplementary file 1 — Supplementary Information. [file 41598_2023_41861_MOESM1_ESM.zip › nutrution/Plot8a-eps-converted-to.pdf]

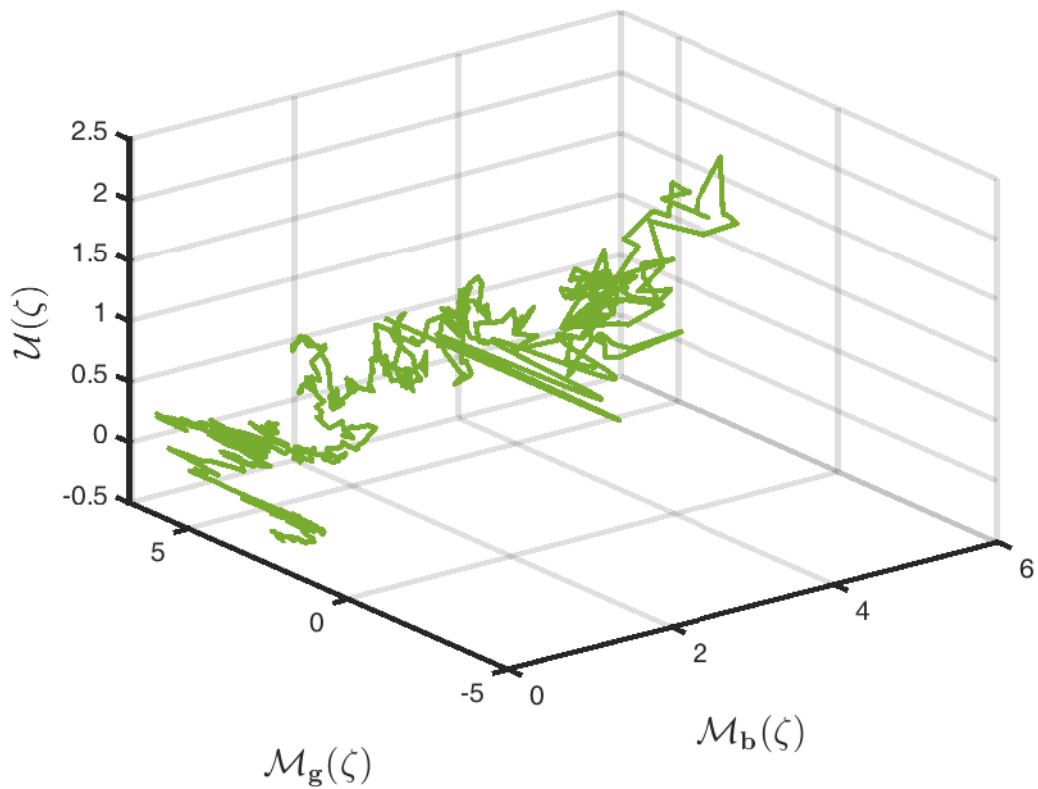

Supplement: Supplementary file 1 — Supplementary Information. [file 41598_2023_41861_MOESM1_ESM.zip › nutrution/Plot8-eps-converted-to.pdf]
